# Asymmetric Access to Structurally Complex 1‑Tetralols through Diels–Alder Cycloadditions of Photogenerated Hydroxy‑*o*‑quinodimethanes and Acrylates

**DOI:** 10.1021/acs.orglett.6c03349

**Published:** 2026-09-02

**Authors:** María González-Marcos, José Trujillo-Sierra, Enara Esteban, José M. Sansano, Ana Sirvent, María de Gracia Retamosa

**Affiliations:** Departamento de Química Orgánica/Institute of Organic Synthesis and Centro de Innovación en Química Avanzada (ORFEO−CINQA), Universidad de Alicante, 03080-Alicante, Spain

## Abstract

Photoenolization/Diels–Alder (PEDA) reactions provide a rapid approach to highly substituted 1-tetralols. Herein, we report a PEDA strategy using activated acrylate-based dienophiles for the synthesis of densely functionalized 1-tetralols with high diastereoselectivity. Isatin-derived acrylates enable access to spirocyclic architectures, while chiral auxiliary-based acrylates allow an asymmetric variant, providing enantioenriched 1-tetralol derivatives with efficient stereocontrol.

## Graphical abstract

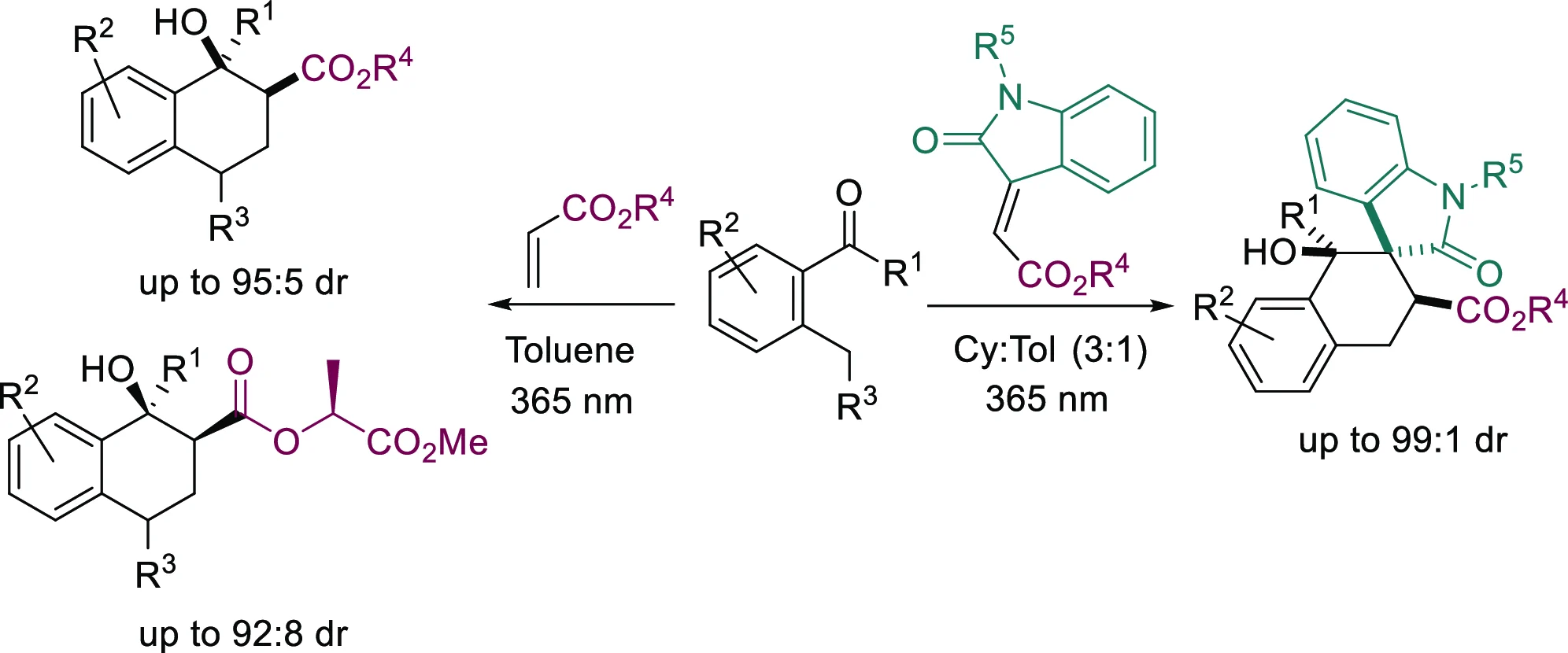

Chiral 1-tetralols are valuable highly rigid structures in medicinal chemistry as bioactive scaffolds, found in commercial drugs and natural compounds (Scheme 1A), and as synthetic intermediates for the preparation of other pharmacologically relevant tetralin derivatives.^1–3^

**Scheme 1:**
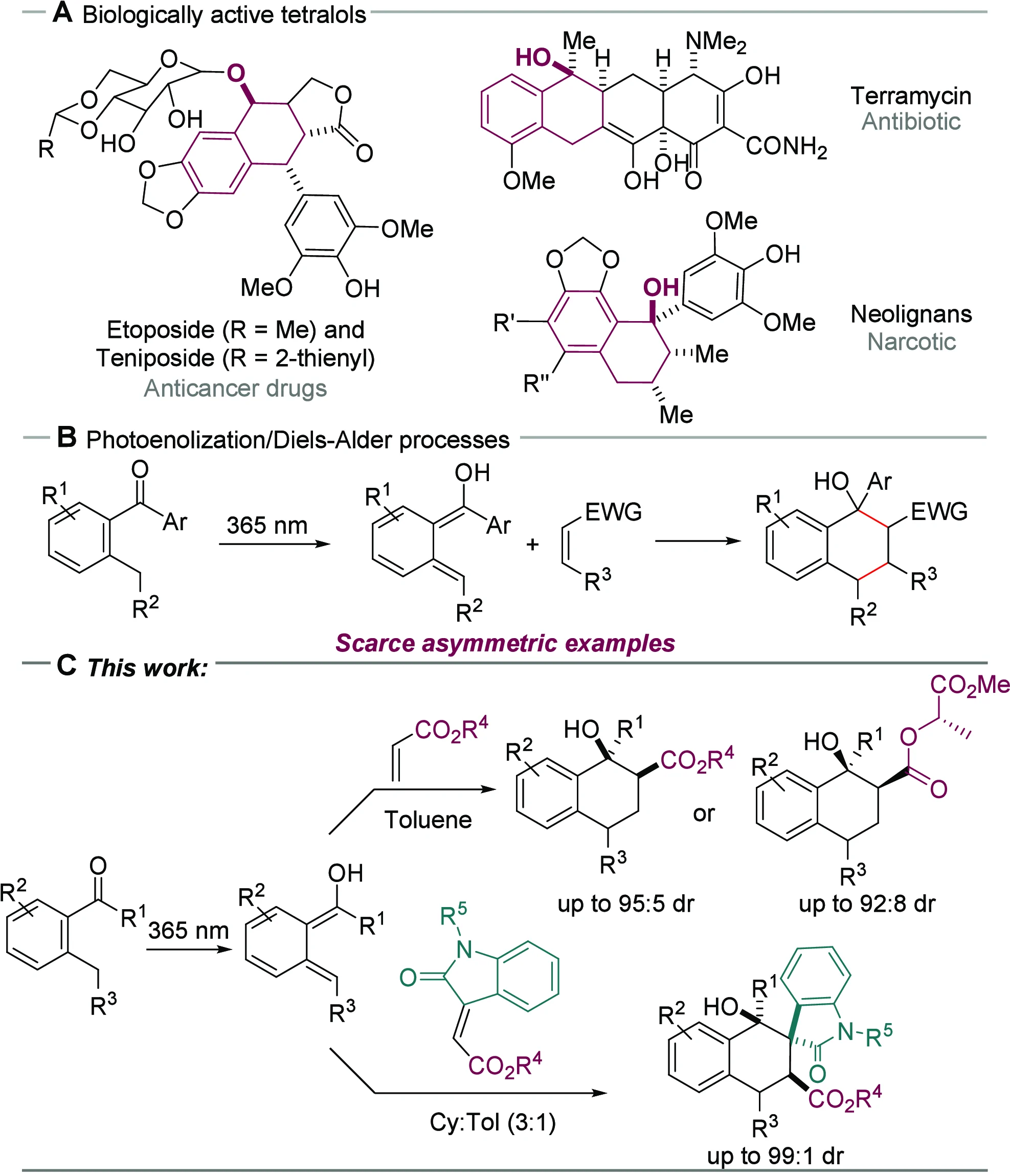
A) Chiral 1-Tetralols in Bioactive Molecules; B) Photoenolization/Diels-Alder (PEDA) Processes; C) This Work: Stereoselective Diels–Alder Reactions of Photogenerated Hydroxy-*o*-quinodimethanes and Acrylate Derivatives

Photoenolization/Diels–Alder (PEDA) processes, which combine the photochemical generation of transient *o*-quinodimethanes (photoenols) with a subsequent [4 + 2] cycloaddition to activated alkenes, have emerged as efficient and atom-economical methods for the direct assembly of highly substituted 1-tetralols (Scheme 1B).^2–9^

Despite significant advances in PEDA chemistry,^10–18^ asymmetric variants remain scarce and are generally limited in scope. Only a few examples of asymmetric PEDA reactions have been reported, including the use of stoichiometric chiral titanium complexes with dienophiles bearing a carbonyl group at the α-position.^10–14^ More recently, catalytic asymmetric variants have been described with *N*-substituted maleimides bearing bulky groups,^15^ chromones,^16^ and alkenylazaarenes,^17^ employing thiourea, chiral *N,N′*-dioxide/Sc^III^, and chiral phosphoric acid catalysts, respectively. In this context, chiral auxiliary-based strategies represent an attractive alternative for asymmetric induction but have received surprisingly little attention.^19,20^

Moreover, the use of highly substituted alkenes as dienophiles in PEDA reactions to access densely functionalized 1-tetralols bearing quaternary stereocenters remains largely unexplored,^6^ with examples involving the concomitant construction of spirocyclic architectures being virtually unknown.

To address these challenges, we envisioned that the use of activated acrylate-based dienophiles could provide a versatile platform for expanding the scope of PEDA reactions toward densely functionalized 1-tetralols. Among them, isatin-derived acrylates are particularly attractive because they enable the formation of spirocyclic 1-tetralols featuring a spiro quaternary carbon center. Building on these results, we developed an asymmetric variant using chiral auxiliary-functionalized acrylates as dienophiles, providing stereocontrolled access to densely substituted 1-tetralols (Scheme 1C).

The Diels–Alder cycloaddition of the hydroxy-*o*-quinodimethane generated in situ under UV light irradiation from 2-methylbenzophenone **1a** and methyl acrylate **2a** was chosen as model system for the optimization of the reaction conditions (Table 1).

**Table 1:**
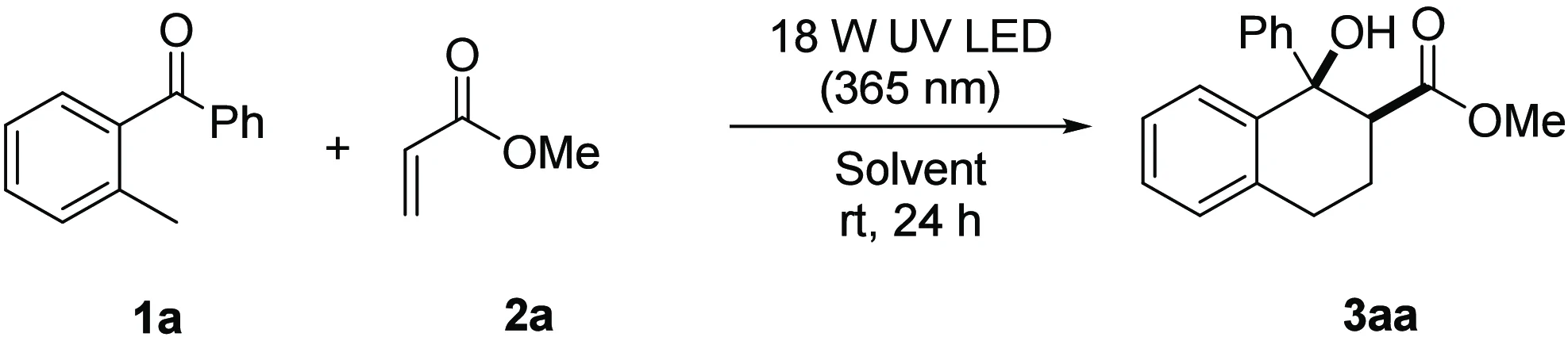
Optimization Reaction Conditions Using Methyl Acrylate **2a** as Dienophile^a^

Entry	Solvent	Conv. (%)^b^	dr^c^
1	Toluene	>95	93:7
2^d^	Toluene	n.r.	n.d.
3	EtOH	28	n.d.
4	THF	40	n.d.
5	DCM	<5	n.d.
6	MeCN	<5	n.d.
7	Cy:Tol 3:1	65	92:8
8^e^	Toluene	85	93:7
9^f^	Toluene	80	93:7
**10** ^g^	**Toluene**	**>95**	93:7

a Reactions were performed with phenyl­(*o*-tolyl)­methanone **1a** (0.3 mmol), methyl acrylate **2a** (0.1 mmol), in 0.1 M of solvent at room temperature under 365 nm UV light for 24 h.

b Conversions were determined by analysis of the ^1^H NMR spectra of the crude reaction mixtures.

c Diastereomeric ratios (dr) were determined by analysis of the ^1^H NMR spectra of the crude reaction mixtures, based on integration of the ester methyl resonances at 3.66 and 3.56 ppm.

d No light irradiation.

e Reaction performed at 0.2 M.

f Reaction performed at 0.05 M.

g Reaction performed with 0.2 mmol of phenyl­(*o*-tolyl)­methanone **1a**. n.r. = no reaction. n.d. = not determined.

An initial experiment conducted in toluene using 3 equiv of 2-methylbenzophenone **1a** proceeded with full conversion, affording product **3aa** with 93:7 diastereomeric ratio (Table 1, entry 1). As anticipated, control experiment in the absence of light under otherwise identical conditions resulted in a complete inhibition of the process (Table 1, entry 2). An extensive screening of solvents was then carried out but none provided better results than toluene in terms of conversion and diastereomeric ratio (Table 1, entries 3–7). Changes in the concentration were also explored (Table 1, entries 8 and 9), and 0.1 M was found to be the optimal value (cf. Table 1, entry 1). The amount of 2-methylbenzophenone **1a** could be reduced to 2 equiv. without compromising the conversion, reaction time or diastereoselectivity (Table 1, entry 10).

After determining the optimal conditions, we investigated their applicability to other activated alkenes (see Supporting Information). A range of substrates, including but-2-enoate and cinnamate derivatives, sulfinyl 1-azadienes, *N*-sulfinylacrylamides, and several methyleneoxindole derivatives, were examined. However, these substrates generally failed to afford the desired cycloadducts, with either decomposition of the starting materials or no reaction being observed under the reaction conditions. Interestingly, in the case of methyleneoxindole **4a**, we detected the formation of spiro compound **5aa**, featuring a 1-tetralol scaffold fused with an oxindole ring through a quaternary stereocenter, together with adduct **6a**, resulting from the [2 + 2] homodimerization of the activated alkene (Table 2). Encouraged by the possibility of accessing such highly substituted 1-tetralol adducts featuring a spiro quaternary carbon center, we sought to optimize the reaction conditions for methyleneoxindole **4a** in order to minimize the formation of byproduct **6a** (Table 2).

**Table 2:**
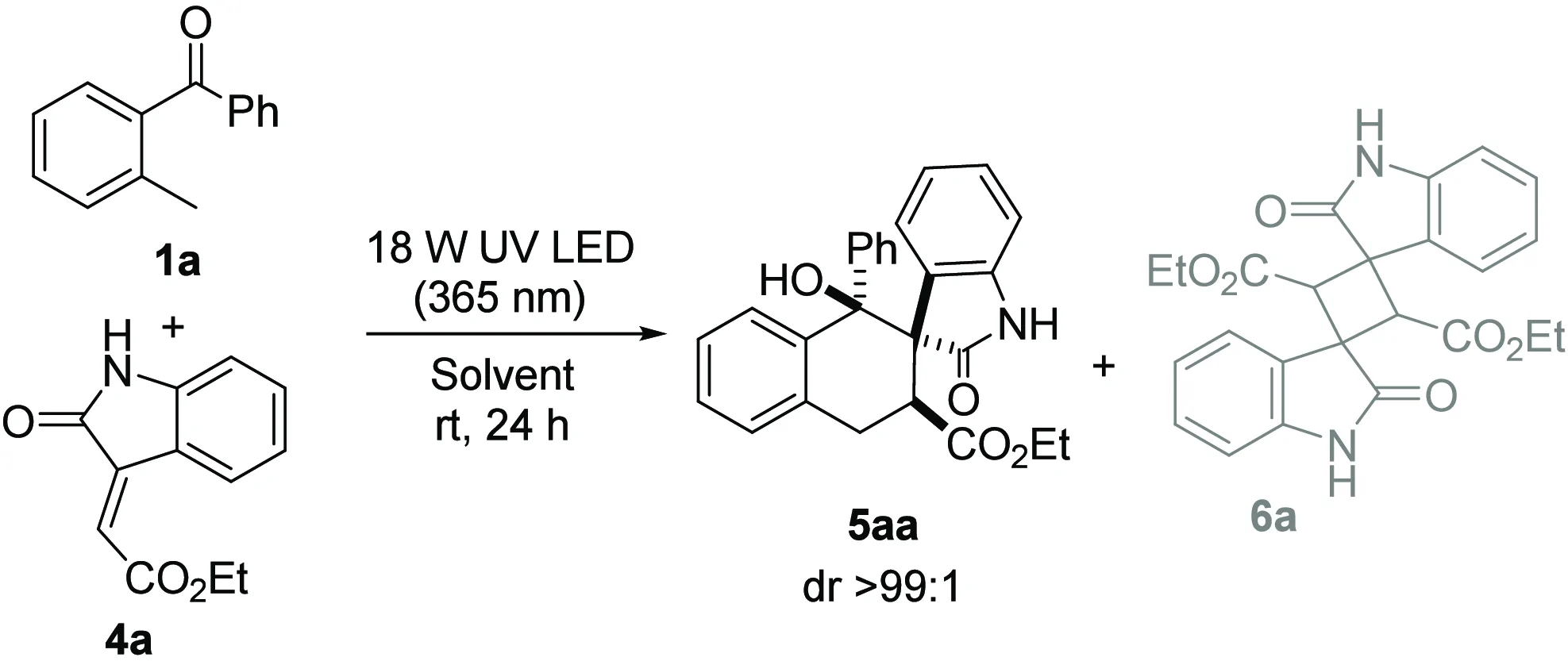
Optimization Reaction Conditions Using β-(Ethoxycarbonyl)­methyleneoxindole **4a** as Dienophile^a^

Entry	Solvent	Conv. (%)^b^	**5aa/6a** ^c^
1	Toluene	>95	40:60
2^d^	Toluene	n.r.	n.d.
3	EtOH	>95	20:80
4	THF	20	60:40
5	DCM	38	10:90
6	MeCN	>95	45:55
7	Cy:Tol 3:1	>95	80:20
8^e^	Cy:Tol 3:1	>95	75:25
**9** ^f^	**Cy:Tol**3:1	**>95**	80:20
10^g^	Cy:Tol 3:1	80	75:25

a Reactions were performed with phenyl­(*o*-tolyl)­methanone **1a** (0.3 mmol), methyl acrylate **4a** (0.1 mmol), in 0.1 M of solvent at room temperature under 365 nm UV light for 24 h.

b Conversions were determined by analysis of the ^1^H NMR spectra of the crude reaction mixtures.

c **5aa:6a** ratios were determined by analysis of the ^1^H NMR spectra of the crude reaction mixtures.

d No light irradiation.

e Reaction performed with 0.15 mmol of phenyl­(*o*-tolyl)­methanone.

f Reaction performed with 0.1 mmol of phenyl­(*o*-tolyl)­methanone.

g Reaction performed with 0.1 mmol of phenyl­(*o*-tolyl)­methanone **1a** and 0.2 mmol of methyleneoxindole **4a**. n.r. = no reaction. n.d. = not determined.

The reaction of 2-methylbenzophenone **1a** and methyleneoxindole **4a** took place with full conversion, and the desired product **5aa** was generated with complete diastereoselectivity; however, significant formation of the homodimerization product **6a** was observed (60% yield, Table 2, entry 1). As previously observed, a control experiment performed in the absence of light under otherwise identical conditions resulted in complete inhibition of the reaction (Table 2, entry 2). A broad solvent screening was subsequently carried out in an attempt to suppress the formation of this undesired byproduct (Table 2, entries 3–7). Although most solvents led to lower conversions and substantial amounts of **6a**, the use of a cyclohexane/toluene mixture (3:1) enabled the formation of the desired product in quantitative conversion, with an improved **5aa/6a** ratio of 80:20 (Table 2, entry 7). The effect of varying the amount of reagents was also investigated (Table 2, entries 8–10), providing similar results in most cases. Notably, the use of equimolar amounts of both reactants was possible without affecting the conversion or the **5aa/6a** ratio (Table 2, entry 9).

After determining the optimal reaction conditions, we investigated the scope of the transformation with a variety of acrylates **2** and methyleneoxindoles **4** (Scheme 2). Methyl, isopropyl, and benzyl acrylates were first evaluated, affording the desired products **3aa-ac** in moderate to good yields and with high diastereoselectivities. Notably, the reaction was readily scaled up from vial-scale reactions to a 2 mmol scale using a larger round-bottom flask while maintaining the same 365 nm irradiation setup, affording cycloadduct **3aa** in an improved isolated yield of 84% (see Supporting Information). This successful scale-up highlights the robustness of the photochemical protocol and its potential for application on a larger scale. In contrast, when *o*-tolylaldehyde was employed as the photoenol precursor in combination with methyl acrylate, the desired product **3ba** was obtained in moderate yield but with high diastereoselectivity. Subsequently, the scope of the methyleneoxindole component **4** was investigated by varying the *N*-substituent, ester group, and aromatic ring. This study revealed a broad tolerance toward structural modifications, with all substrates providing the corresponding spirocyclic 1-tetralol products with high to complete diastereoselectivities, albeit in moderate yields in most cases. Moreover, the introduction of a cyano group instead of the ester functionality was feasible, as exemplified by 2-(2-oxoindolin-3-ylidene)­acetonitrile **4d**, which delivered the desired spirocyclic adduct **5ad** in 53% yield and with complete diastereoselectivity. The relative configuration of compound **5ad** was confirmed by X-ray diffraction analysis and the configuration of the rest of the compounds was assigned analogously, assuming that they are formed through the same mechanistic pathway.

**Scheme 2:**
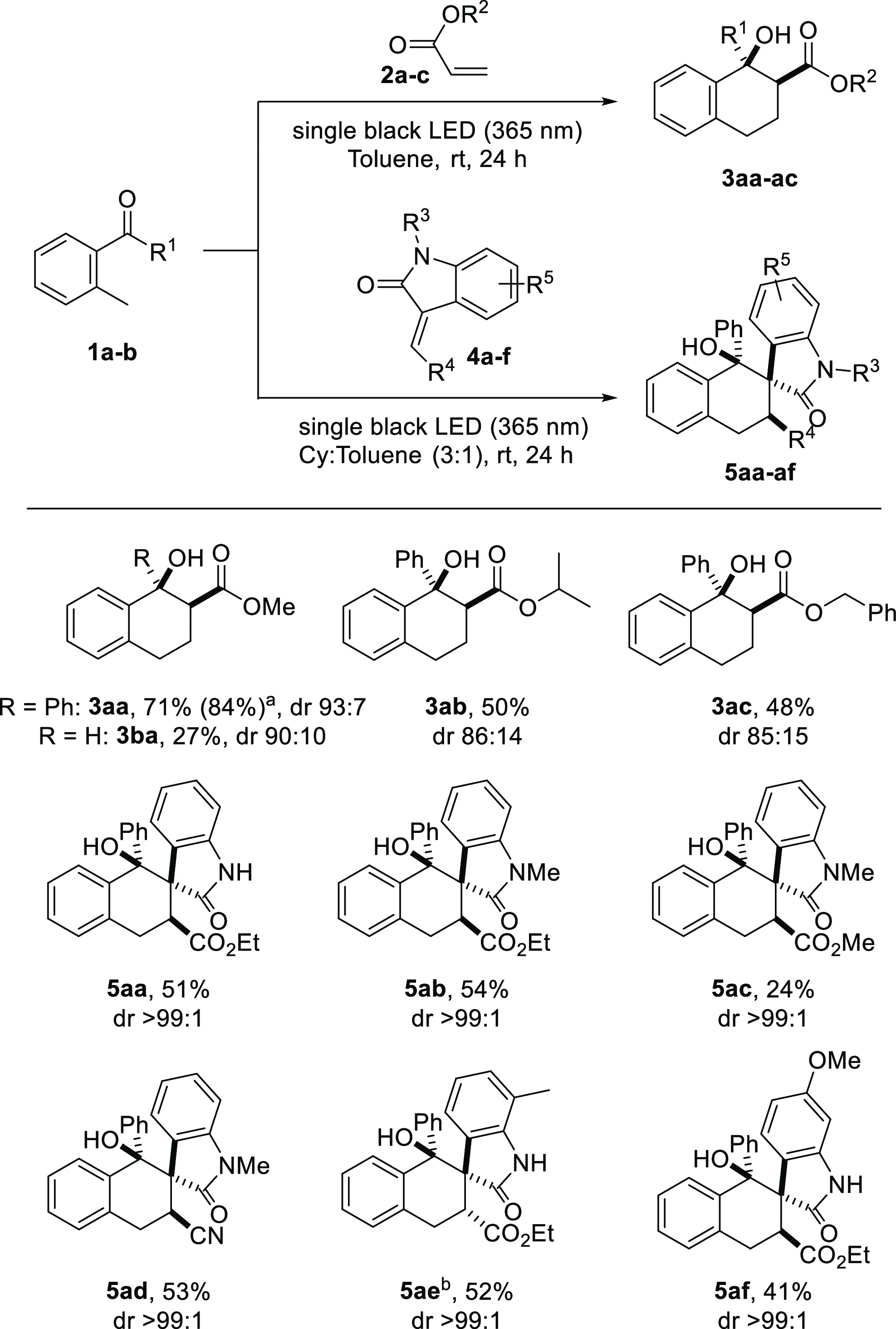
Scope of the Reaction with Different Acrylates **2** and Methyleneoxindoles **4**

a Reaction was performed on a 2 mmol scale.
b Performed with (*Z*)-methyleneoxindole **4e**.

We then examined the use of a series of chiral thiourea-based organocatalysts to achieve an enantioselective version of the reaction using either methyl acrylate **2a** or methyleneoxindole **4a**. However, these studies were unsuccessful. In the case of methyl acrylate **2a**, conversions below 30% were obtained, whereas the use of methyleneoxindole **4a** resulted in increased formation of the homocoupling byproduct **6a** (see Supporting Information).

To access enantiomerically pure products, we therefore adopted a chiral auxiliary strategy by incorporating (*S*)-methyl lactate into the ester moiety of both the acrylate and methyleneoxindole substrates. Importantly, the chiral auxiliary can be readily removed to afford the corresponding carboxylic acid without epimerization, following a methodology previously optimized in our group for densely substituted pyrrolidines bearing the same auxiliary, using trimethyltin hydroxide.^21^ Given the low cost of the auxiliary, its recovery was not considered necessary. The reaction conditions were subsequently reoptimized for both substrate classes (see Supporting Information). In both cases, toluene proved to be the optimal solvent, providing the highest levels of diastereoselectivity. Notably, the introduction of the pre-existing stereogenic center of the (*S*)-methyl lactate chiral auxiliary results in four possible diastereoisomers of the **3** adducts. Of these, only two were formed preferentially, while the two remaining diastereoisomers were each formed in less than 5%.

Under these optimized conditions, the synthetic scope of the reaction was investigated using a range of substituted 2-methylbenzophenones **2** as photoenol precursors (Scheme 3). A broad tolerance toward both structural and electronic variation of the benzophenone substrate was observed, providing access to the corresponding tetrahydronaphthalenol derivatives **3ad–gd** with high diastereoselectivities in most cases and moderate to good yields. Substituents with different electronic properties on the enolizable aromatic ring were well tolerated, affording the corresponding products **3ad–gd** without significant loss of efficiency. In addition, a prochiral center at the *ortho*-benzylic position of the photoenol precursor was compatible with the transformation, delivering the desired adduct with an 82:18 diastereomeric ratio. Likewise, the use of *o*-tolylaldehyde **1b** afforded the corresponding product **3bd** with a 90:10 diastereomeric ratio. Unfortunately, when methyleneoxindole **4g** was employed, only moderate diastereoselectivity was achieved together with a low yield, owing to the competing homocoupling of **4g**. The absolute configuration of compound **3ad** was confirmed by X-ray diffraction analysis and the configuration of the rest of the compounds was assigned analogously, assuming that they are formed through the same mechanistic pathway.

**Scheme 3:**
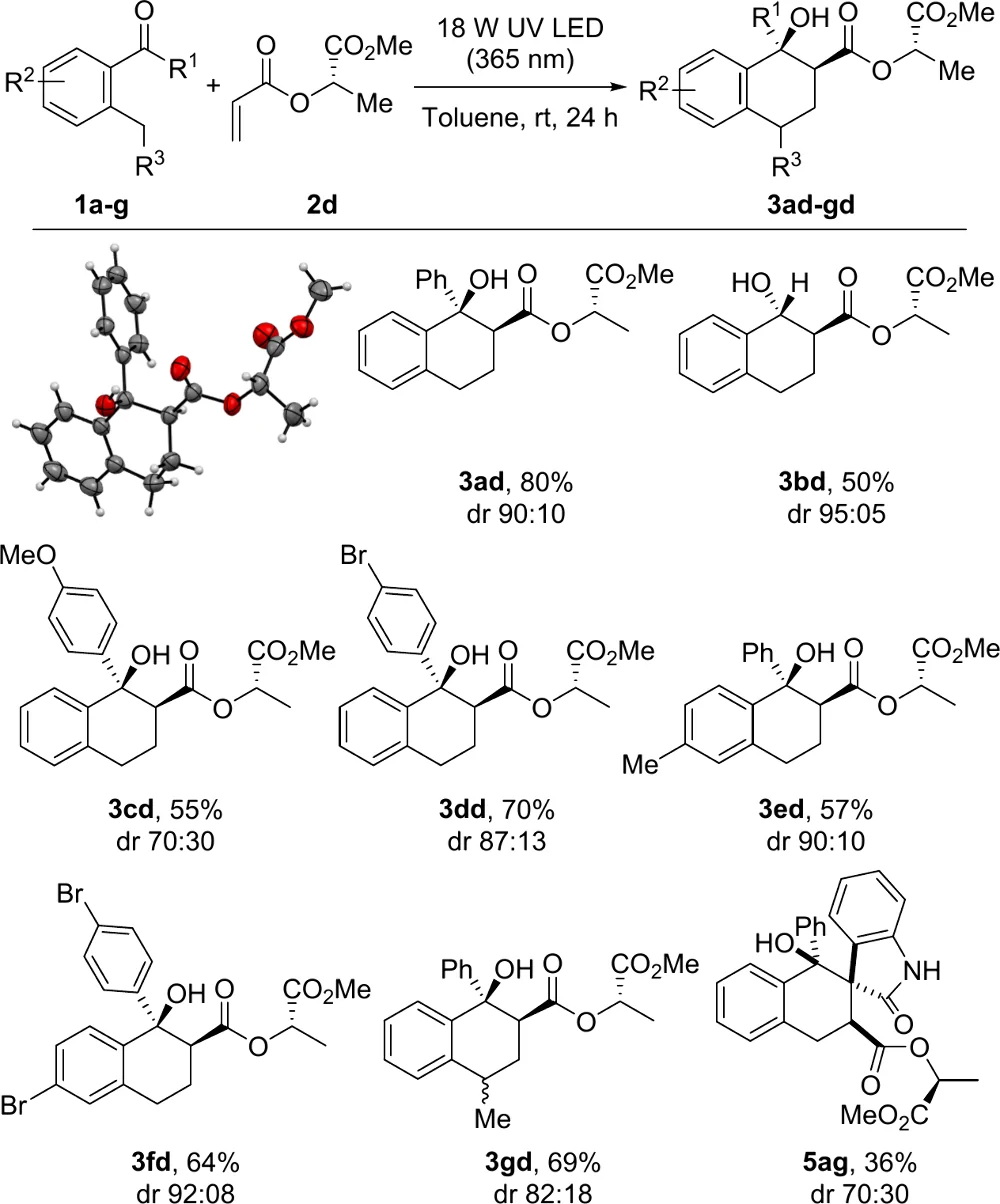
Scope of the Diastereoselective Reaction Using (*S*)-Methyl Lactate-Derived Acrylate **2d** and Methyleneoxindole **4g**

It is worth noting that the diastereomeric ratios reported throughout Schemes 2 and 3 were determined from the crude reaction mixtures. Upon purification, some products were isolated as single diastereomers (>99:1 dr), whereas in other cases the dr showed only a slight increase or decrease (see Supporting Information).

The origin of the observed diastereoselectivity was investigated through computational analysis. The calculated transition states indicate that the *exo* approach is favored over the *endo* alternative owing to a stabilizing hydrogen-bonding interaction between the two reaction partners in the [4 + 2] cycloaddition transition state (Figure 1). Accordingly, TS1 was found to be lower in energy than TS2. The higher energy of TS2 is attributed to increased steric repulsion between the ester moiety of the chiral auxiliary and the benzo-fused framework, thereby accounting for the preferential formation of the observed diastereomer.

**Figure 1:**
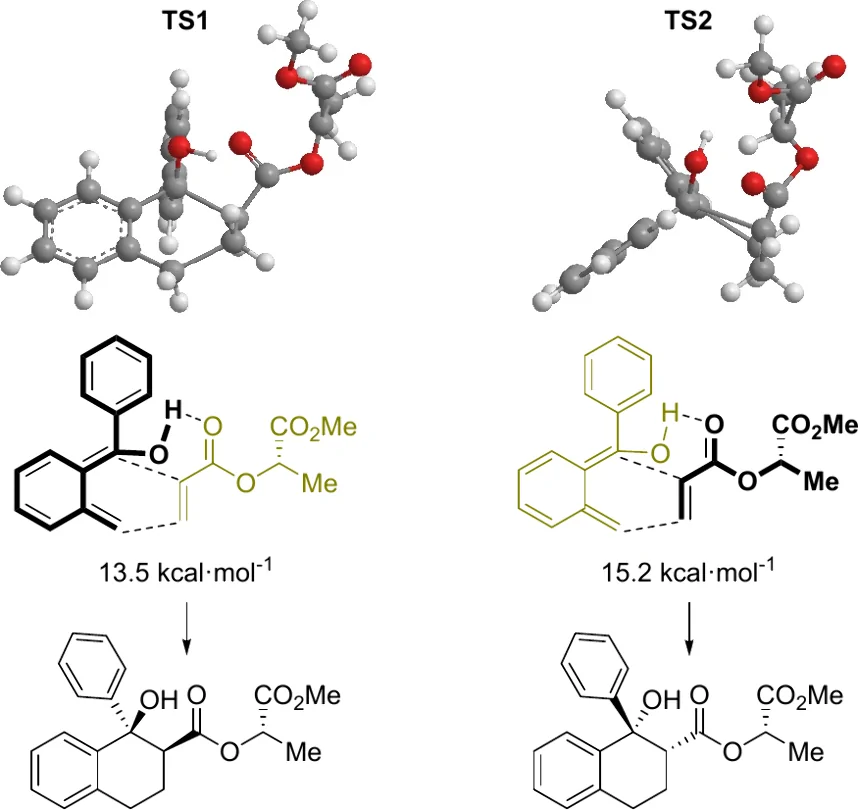
Proposed transition-state models accounting for the observed diastereoselectivity. DFT calculations for optimization of saddle points were performed in terms of the ONIOM method implemented in the Gaussian16 suite of programs. Atoms in the higher layer were represented in ball&stick. Transparent ball&stick atoms represent the ones included in the lower layer. In the high-level layer, the electron correlation was taken into account by using the hybrid simple functional B3LYP3.

To demonstrate the importance of hydrogen-bonding interactions in the observed diastereoselectivity, the reaction was performed in EtOH, which was employed to disrupt the hydrogen-bonding interaction between the hydroxyl group of the photoenol and the carbonyl group of the ester moiety in **2d** (Scheme 4). Under these conditions, the desired product was formed only in low proportion, with a significantly reduced diastereoselectivity (dr = 56:44) (see the Supporting Information).

**Scheme 4:**
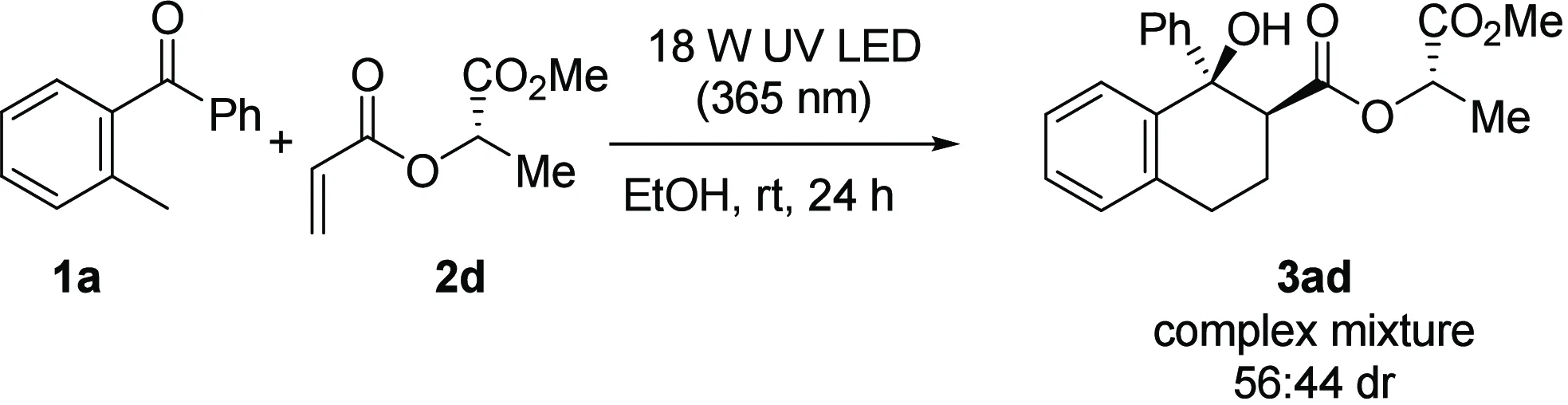
Control Experiment in EtOH to Assess the Role of Hydrogen Bonding in Diastereoselectivity

In conclusion, we have developed a stereoselective PEDA strategy for the synthesis of highly substituted 1-tetralols using activated acrylate-based dienophiles. This approach enables the efficient construction of densely functionalized frameworks, including spirocyclic oxindole-fused tetralols bearing quaternary stereocenters. Furthermore, the incorporation of a chiral auxiliary allowed the development of an asymmetric variant, providing enantioenriched 1-tetralol derivatives with effective stereocontrol. Although asymmetric PEDA reactions remain scarce, these results broaden their synthetic scope and highlight the potential of activated acrylate dienophiles for the stereoselective synthesis of complex tetralin architectures.

## Supplementary Material



## Data Availability

The data underlying this study are available in the published article and its Supporting Information.

## Notes

Experimental details, characterization data, computational methods, X-ray crystal data, and NMR spectra (PDF)
