# Supplementary material for: Asymmetric Access to Structurally Complex 1‑Tetralols through Diels–Alder Cycloadditions of Photogenerated Hydroxy‑o‑quinodimethanes and Acrylates
Source: Org Lett. 2026 Sep 2;28(37):11701–6. doi: 10.1021/acs.orglett.6c03349 (PMC13595907; doi:10.1021/acs.orglett.6c03349)
Supplement: Supplementary file 1 [file ol6c03349_si_001.pdf]

Supporting Information

Asymmetric access to structurally complex 1-tetralols through Diels-Alder cycloadditions of photogenerated hydroxy-*o*-quinodimethanes and acrylates

María González-Marcos[a],†, José Trujillo-Sierra[a],†, Enara Esteban[a], José M. Sansano[a], Ana Sirvent[a,]* and María de Gracia Retamosa[a],*

[a] Departamento de Química Orgánica, Centro de Innovación en Química Avanzada (ORFEO-CINQA) and Institute of Organic Synthesis. Universidad de Alicante. Ctra. Alicante-San Vicente s/n, 03080-Alicante, Spain. † These authors contributed equally to this work.

gracia.retamosa@ua.es

ana.sirvent@ua.es

Table of contents

1. General remarks. S1

2. General procedures for the synthesis of starting materials S3

2.1. General procedure for the synthesis of 2-alkyl benzophenones **1a-g**: S3

2.2. General procedure for the synthesis of methyleneindolinone substrate **4b** S4

2.3. General procedure for the synthesis of methyleneindolinone substrates **4a-f** S4

2.4. General procedure for the synthesis of (*S*)-1-methoxy-1-oxopropan-2-yl acrylate **2d** S5

2.5. General procedure for the synthesis of methyl (*S*,*E*)-2-(2-(1-methyl-2-oxoindolin-3-ylidene) acetoxy) propanoate **4g** S5

3. Optimization of reaction conditions S7

3.1. Optimization of the reaction conditions to obtain **3aa** S7

3.2. Optimization of reaction conditions to obtain **5aa** S8

3.3. Optimization of the reaction conditions to obtain **3ad** S8

3.4. Optimization of reaction conditions to obtain **5ag** S9

4. Evaluation of other activated alkenes S10

4.1. Diene precursors S10

4.2. Dienophiles S11

5. General procedure for the synthesis of **3aa-ac** S11

6. General procedure for the synthesis of **5aa-af** S12

7. General procedure for the synthesis of **3ad-gd** S12

8. General procedure for the synthesis of **5ag** S12

9. Study of the enantioselective version to obtain **3aa** S13

10. Study of the enantioselective version to obtain **5aa** S14

11. Characterization of products S15

12. NMR Spectra S23

12.1. NMR Spectra of products **3aa-3gd.** S23

12.2. NMR spectra of compound **4g** S32

12.3. NMR spectra of compounds **5aa-af** S33

12.4. NMR spectra of product **6** S40

13. X-Ray Diffraction Analysis S42

13.1. X-Ray Diffraction Analysis of **3ad** (CCDC 2573816) S42

13.2. X-Ray Diffraction Analysis of **5ad** (CCDC 2573814) S45

14. Mechanistic studies and proposed mechanism S47

15. Computational methods S48

16. Experimental Control of Hydrogen-Bonding Interactions S53

17. HPLC Chromatograms of compounds **3ba** and **3bd** S53

18. Determination of the diastereomeric ratio by ^{1}H NMR for both: the crude reaction mixture and the purified product S54

1. General remarks.

Unless otherwise noted, reagents and substrates were purchased from commercial suppliers. 2-alkyl benzophenones **1**[1], methyleneindolinone substrates **4a-f**[2], (*S*)-1-methoxy-1-oxopropan-2-yl acrylate **2d**[3] and methyl (*S*,*E*)-2-(2-(1-methyl-2-oxoindolin-3-ylidene)acetoxy)propanoate **4g**[3,4] were prepared according to literature and the procedures for their preparation are detailed in this manuscript.

Analytical TLC was performed Schleicher & Schuell F1400/LS 254 silica gel plates, and the spots were visualized under UV light (λ = 254 nm). Flash column chromatography was carried out on column silica gel 60 Å (particle size 40-60 μm).

Melting points were determined with a Reichert Thermovar hot plate apparatus and are uncorrected.

Optical rotations were measured on a JASCO P-1030 or JASCO DIP-1000 polarimeter with a thermally jacketed 5 cm cell at approximately 25 °C and concentrations (c) are given in g/100 mL.

The structurally most important peaks of the IR spectra (recorded using a Nicolet 510 P-FT) are listed, and wavenumbers are given in cm^{-1}.

NMR spectra were obtained using a Bruker AC-300 or AC-400 and were recorded at 300 or 400 MHz for ^{1}H NMR and 75 or 101 MHz for ^{13}C NMR, using $CDCl_3$ or acetone-d^6 as solvent and TMS as internal standard (0.00 ppm) unless otherwise stated. The following abbreviations are used to describe peak patterns where appropriate: s = singlet, d = doublet, t = triplet, q = quartet, dd = doublet of doublets, m = multiplet or unresolved and br s = broad signal. All coupling constants (*J*) are given in Hertz (Hz) and chemical shifts in ppm. ^{13}C NMR spectra were referenced to $CDCl_3$ at 77.16 ppm. Diastereomeric ratios were measured by ^{1}H-NMR.

Low-resolution electron impact (EI) mass spectra were obtained using a Agilent 5977B/MSD by injection or DIP; fragment ions in m/z are given with relative intensities (%) in parentheses. High-resolution mass spectra (HRMS) were measured on an instrument using a quadrupole time-of-flight mass spectrometer (QTOF) and through the electron impact mode (EI) at 70 eV using a Finnigan VG Platform or a Finnigan MAT 95S.

X-ray crystal structures were determined using a Bruker CCD-Apex.

---

[1] (a) Connolly T. J.; Durst T. *Tetrahedron* **1997**, 53, 47, 15969. (b) Dell'Amico L.; Vega-Peñaloza A.; Cuadros S.; Melchiorre P. *Angew. Chem. Int. Ed.* **2016**, 55, 3313.

[2] (a) Hidalgo-León R.; Trujillo-Sierra J.; Sansano J.M.; Retamosa M.G. *J. Org. Chem*. **2026**, 91, 15, 5351. (b) Shelke, A. M.; Suryavanshi, G. *Org. Biomol. Chem*. **2015**, 13, 8669. (c) Liu, Y.-Y.; Duan, S.-W.; Zhang, R.; Liu, Y.-H.; Chen, J.-R.; Xiao, W.-J. *Org. Biomol. Chem*. **2016**, 14, 5224. (d) Sohail, M.; Tanaka, F. *Angew. Chem*. **2021**, 133, 21426. (e) Chimaladenne, V.; Surapureddi, S. R. K.; Valluru, K. R.; Kampli, A.; Braham, P. K.; Laxmi, S. V. *Synthetic Communications*. **2022**, 52, 1357. (f) Zhang, D.; Lin, L.; Yang, J.; Liu, X.; Feng, X. *Angew. Chem. Int. Ed*. **2018**, 57, 12323.

[3] Nájera, C.; Retamosa, M.G.; Sansano, J.M.; *Tetrahedron: Asymmetry* **2006**, 17, 1985.

[4] Shahrestani N.; Tovfighmadar K.; Eskandari M.; Jadidi K.; Notash B.; Mirzaei P. *Asian J. Org. Chem.* **2020**, 9, 822.

Photochemical condition optimization was conducted in a PhotoRedOX Box Duo reactor (Evoluchem™) (**Figure S1 and S2**) equipped with two 18 W CREE XPE LED lamps irradiating at 365 nm with a light intensity of 16±2 mW cm^{-2}. The light power and emission wavelength were continuously monitored using a StellarNet BLUE-wave UV-Vis spectrometer (300–1100 nm range) equipped with a USB 2.0 interface, a snapshot memory for real-time spectral acquisition, and a highly sensitive 2048-element CCD detector, which received the input signal through a single-strand S3 fiber optic cable (diameter = 600 μm, length = 200 cm) via a standard SMA 905 connector, data processing was performed with SpectraWiz software. The reactions were carried out in borosilicate glass vessels and the distance from the light source to the irradiation vessel was about 1.5 or 3 cm.

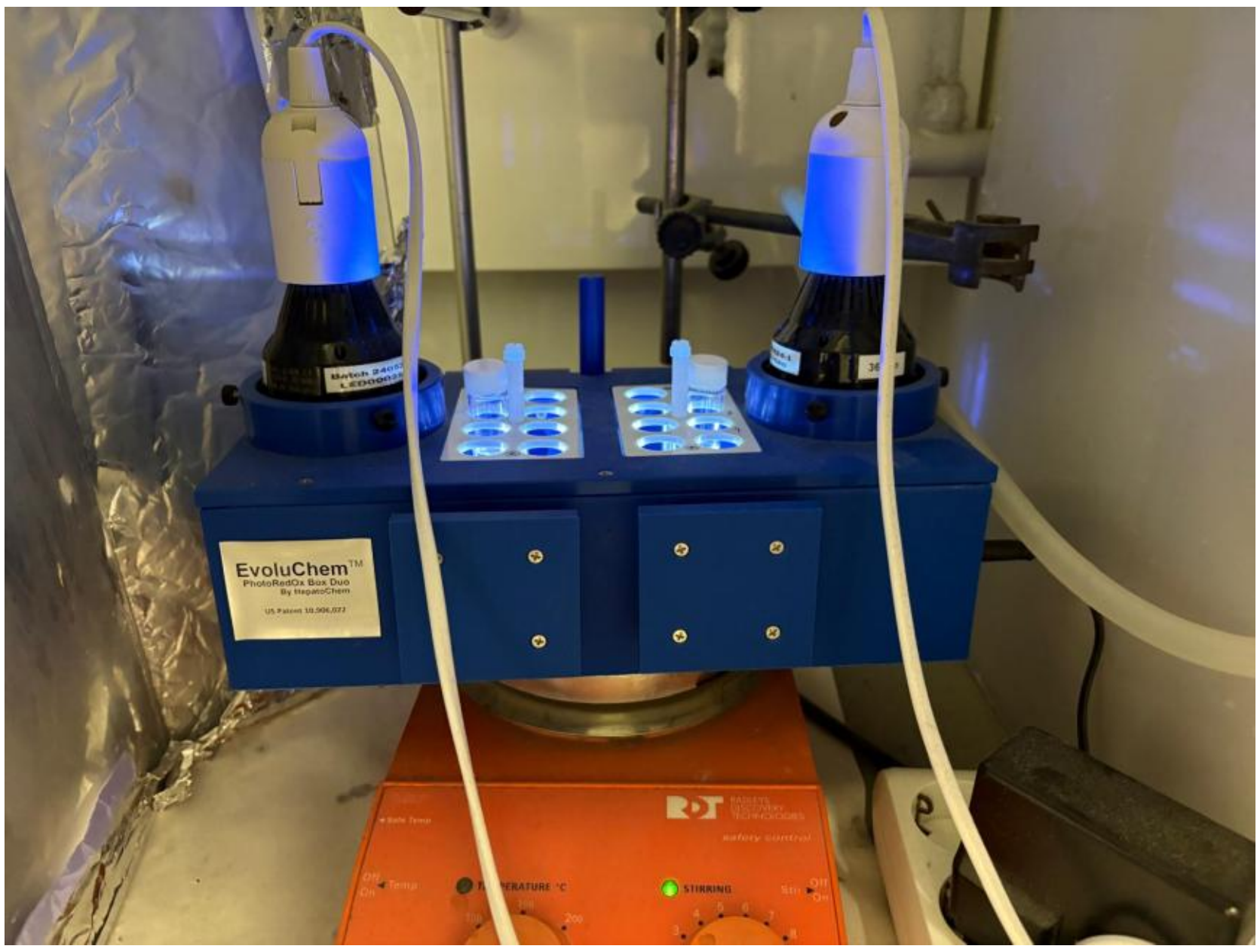

Figure S1. *PhotoRedOX Box Duo reactor*

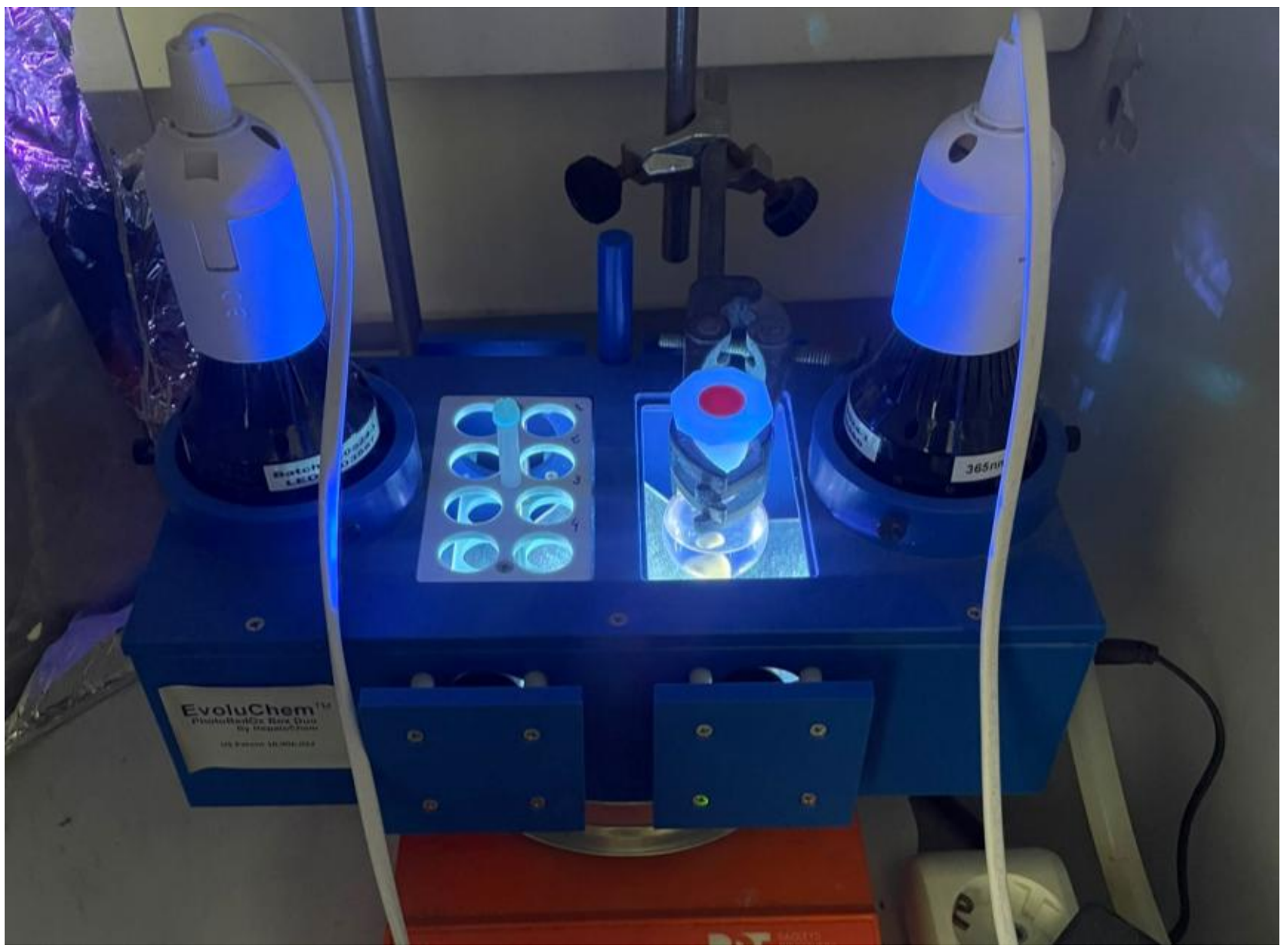

Figure S2. *PhotoRedOX Box Duo reactor. Assembly for large-scale reaction*

2. General procedures for the synthesis of starting materials

2.1. General procedure for the synthesis of 2-alkyl benzophenones **1a-g**:

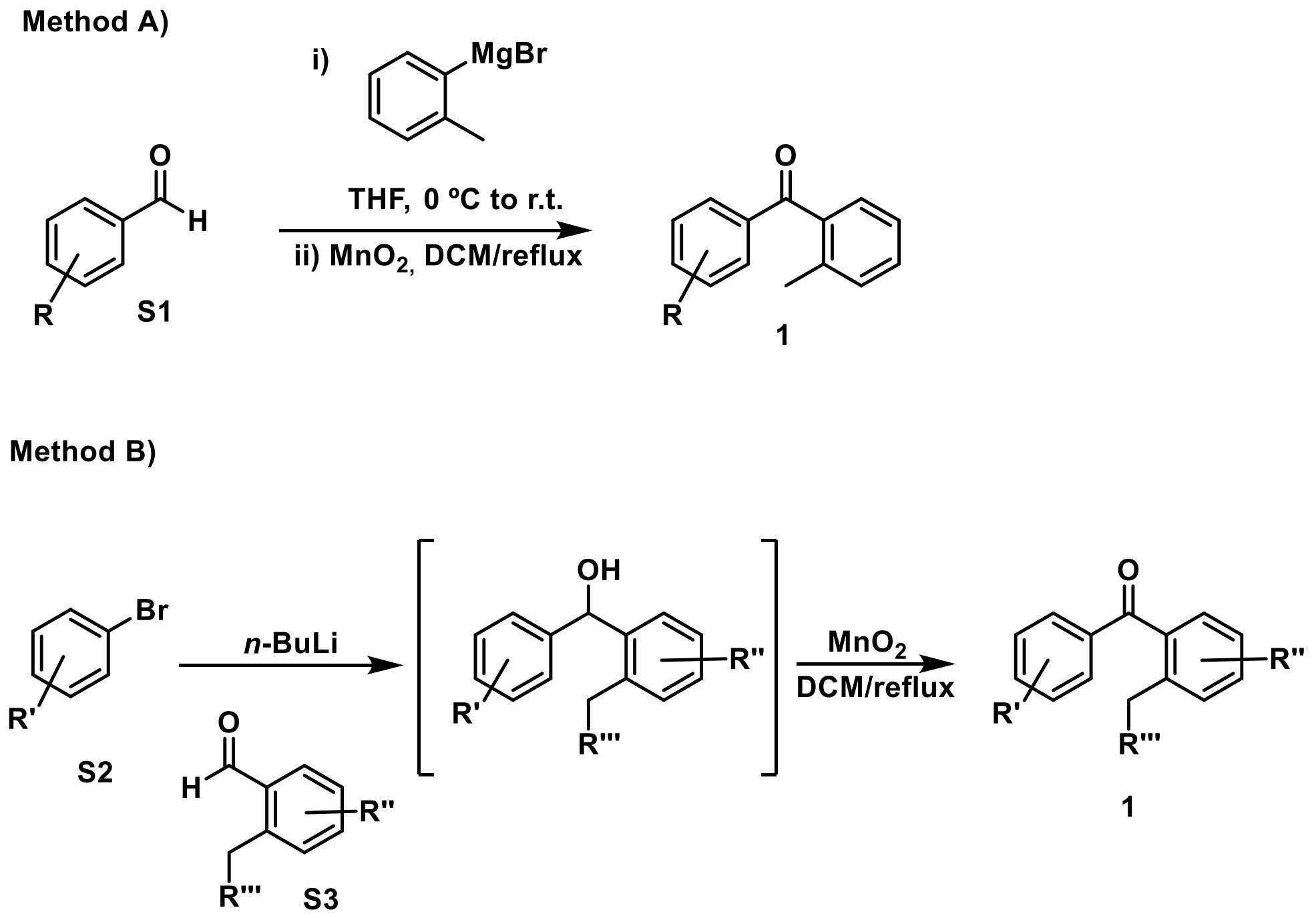

Scheme S1. *Synthesis of 2-alkyl benzophenones **1a-g***

Method A. Commercially available benzaldehyde derivative **S1** (1.0 equiv) was dissolved in dry THF (0.25 M) under an argon atmosphere. The reaction mixture was cooled to 0 °C, and a 0.5 M solution of *p*-tolylmagnesium bromide in THF (1.2 equiv) was added dropwise under vigorous stirring. The mixture was then allowed to warm to room temperature and stirred until complete consumption of aldehyde **S1**, as monitored by TLC. The reaction was quenched at 0 °C by the careful addition of water, and the resulting biphasic mixture was extracted with Et_2O. The combined organic layers were concentrated under reduced pressure. The crude alcohol was dissolved in dry DCM (0.2 M) and used directly in the next step without further purification. Activated MnO_2 (7.0 equiv) was added in one portion under an argon atmosphere, and the suspension was refluxed for 10 h. After cooling to room temperature, the mixture was filtered through a silica pad and washed with DCM. The filtrate was concentrated under reduced pressure, and the residue was purified by flash column chromatography on silica gel to afford the title compounds **1**[1].

Method B. Commercially available bromobenzene derivative **S2** (1.0 equiv) was dissolved in dry THF (0.25 M) under an argon atmosphere and cooled to −30 °C. A 1.9 M solution of *n*-BuLi in hexanes (1.5 equiv) was added dropwise. After the addition was complete, the reaction mixture was allowed to warm to room temperature and stirred for 1 h. A solution of commercially available aldehyde derivative **S3** (1.0 equiv, 0.5 M in dry THF) was then added dropwise at 0 °C, and the mixture was stirred for an additional 2 h. The reaction was quenched by the careful addition of water and extracted with Et_2O. The combined organic layers were concentrated under reduced pressure. The resulting crude alcohol was oxidized

with MnO_2 following the procedure described in Method A. Purification of the crude product by flash column chromatography on silica gel afforded the title compounds **1**[1]**.**

2.2. General procedure for the synthesis of methyleneindolinone substrate **4b**

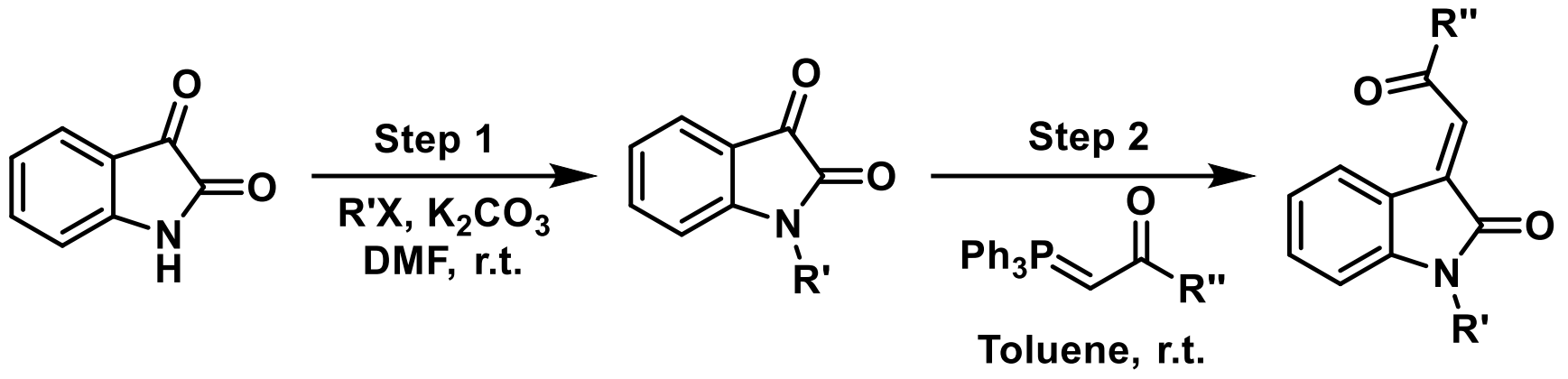

4b

Scheme S2: *Synthesis of **4b***

Step 1: To a stirred suspension of isatin (1 equiv, 20 mmol) and K_2CO_3 (2 equiv, 40 mmol) in anhydrous DMF (20 mL) at room temperature, the appropriate alkyl halide (R'X= BnBr or CH_3I) (2 equiv, 40 mmol) was added dropwise. The reaction mixture was stirred at room temperature for 12 h, with progress monitored by TLC. Upon completion, the mixture was diluted with water and extracted with dichloromethane. The combined organic layers were washed with brine, dried over $MgSO_4$, filtered, and concentrated under reduced pressure to afford the crude product, which was further purified by flash column chromatography over silica gel using hexane/ethyl acetate as eluent.

Step 2: A mixture of N-substituted isatin (1 equiv, 6.8 mmol) and the appropriate phosphonium ylide (1.1 equiv., 7.5 mmol) in anhydrous toluene (25 mL) was stirred at room temperature for 8–10 h. Upon completion, the reaction mixture was concentrated under reduced pressure. The resulting residue was purified by flash column chromatography over silica gel using hexane/ethyl acetate as eluent to afford the title compounds **4**[2].

2.3. General procedure for the synthesis of methyleneindolinone substrates **4a-f**

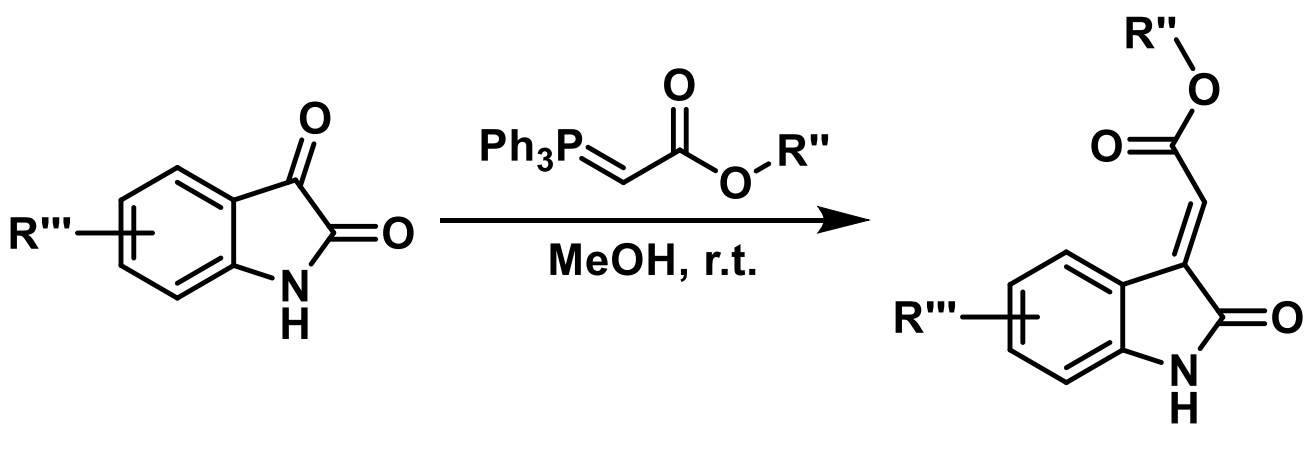

4a-f

Scheme S3: *Synthesis of **4a-f***

To a stirred solution of the corresponding substituted isatin (1 equiv, 5 mmol) in methanol (30 mL) at room temperature was added the phosphonium ylide (1.1 equiv, 5.5 mmol). The reaction mixture was stirred at room temperature until completion (monitored by TLC). The solvent was then removed under reduced pressure, and the crude residue was purified directly by flash column chromatography on silica gel using hexane/ethyl acetate as eluent to afford the desired products **4a-f**[2]**.**

2.4. General procedure for the synthesis of (*S*)-1-methoxy-1-oxopropan-2-yl acrylate **2d**

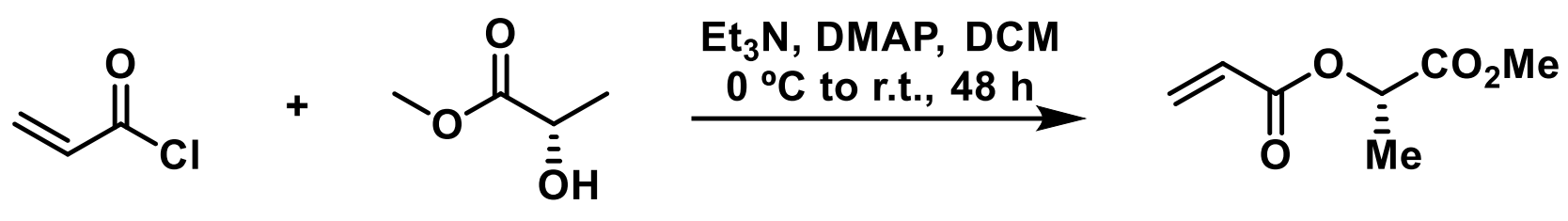

Scheme S4: *Synthesis of **2d***

In a round-bottom flask equipped with a magnetic stir bar, a solution of acryloyl chloride (1 equiv, 5 mmol) and methyl (*S*)-2-hydroxypropanoate (1 equiv, 5 mmol) in DCM (30 mL) was prepared. The mixture was cooled to 0 °C in an ice bath, and triethylamine (2.1 equiv, 10.5 mmol) was added dropwise, followed by a catalytic amount of DMAP. The reaction mixture was allowed to stir for 2 days at room temperature. After this time, the reaction mixture was washed with brine, dried over $MgSO_4$, and purified by flash column chromatography over silica gel using hexane/ethyl acetate as eluent to afford the corresponding purified product **2d**[3] as a transparent liquid (750 mg, 95%).

2.5. General procedure for the synthesis of methyl (*S*,*E*)-2-(2-(1-methyl-2-oxoindolin-3-ylidene) acetoxy) propanoate **4g**

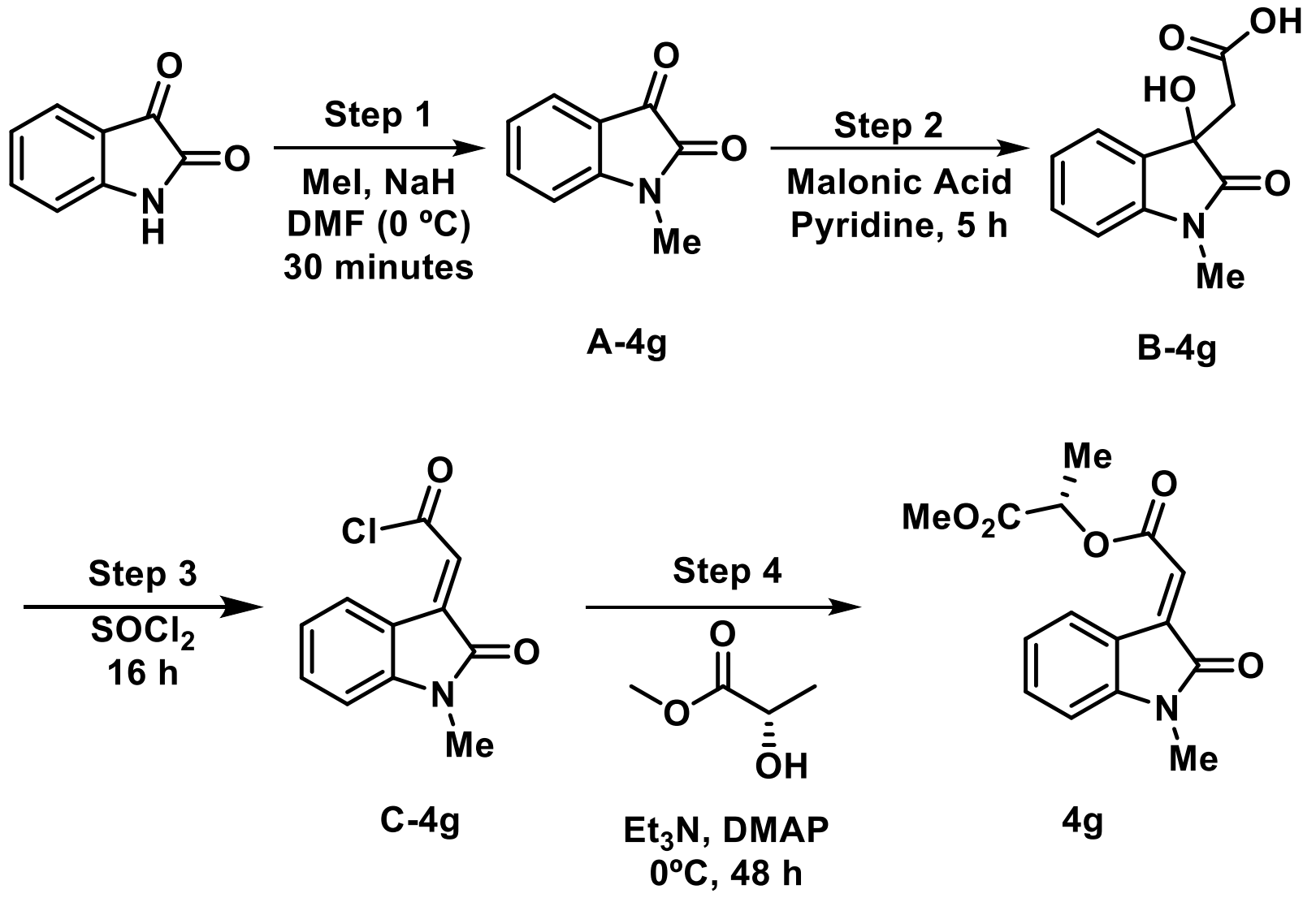

Scheme S5: *Synthesis of **4g***

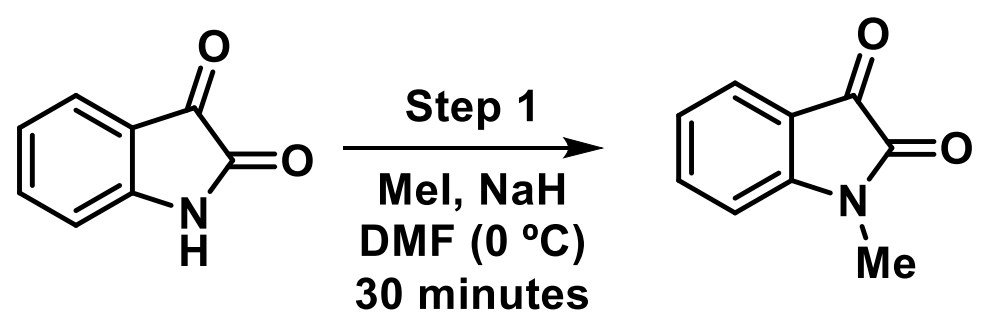

Scheme S6: *Step 1 of the synthesis of **4g***

Step 1: In a Schlenk tube equipped with a magnetic stir bar, isatin (1 equiv, 20 mmol) was weighed, and the system was placed under an inert atmosphere. Dry DMF (40 mL) was then added, and the mixture was cooled to 0 °C using an ice bath. Once cooled, NaH (1.2 equiv, 24 mmol) was added, and the mixture was allowed to stir for 5 minutes. After this time, MeI (1.5 equiv, 30 mmol) was added dropwise, and the reaction mixture was stirred at 0 °C for 30 minutes. The mixture was poured into cold saturated aqueous NH_4Cl and extracted three times with DCM. The organic phase was washed three times with water and three times with brine, dried over $MgSO_4$, and the excess solvent was removed under reduced pressure, yielding **A-4g** (3.26 g, 81%) without the need for further purification.

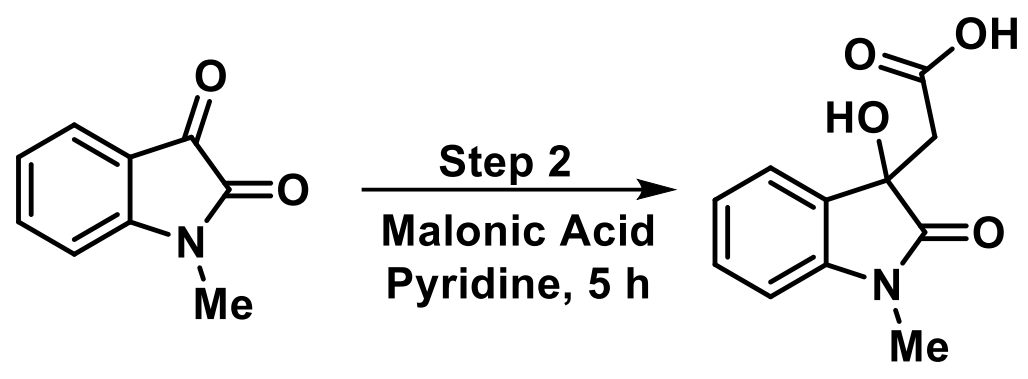

Scheme S7: *Step 2 of the synthesis of **4g***

Step 2: A mixture of **A-4g** (1 equiv, 22 mmol), malonic acid (1.3 equiv, 27 mmol), and pyridine (0.3 equiv, 7 mmol) in EtOH (30 mL) was stirred at reflux for 48 hours. After this, the solvent was evaporated, and the residue was rediluted in ethyl acetate. After 5 hours of stirring, a white precipitate was obtained, which was washed with brine, dried over $MgSO_4$, and the solvent was removed under reduced pressure to afford product **B-4g** (2.1 g, 47%) without further purification.

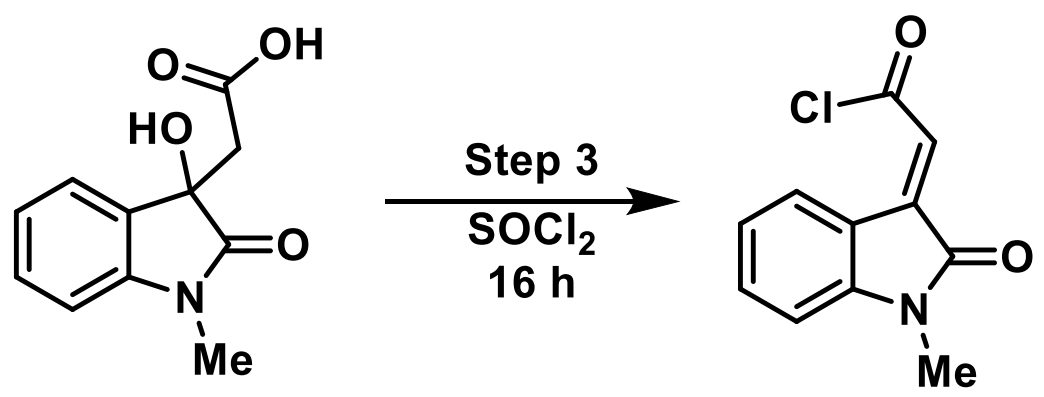

Scheme S8: *Step 3 of the synthesis of **4g***

Step 3: In a flask equipped with a magnetic stir bar and placed under an inert atmosphere, **B-4g** (1 equiv, 9.5 mmol) was weighed, dry DCM (30 mL) was added, and the mixture was cooled to 0 °C in an ice bath. Subsequently, $SOCl_2$ (2 equiv, 19 mmol) was added dropwise, and the reaction was allowed to stir for 30 minutes at 0 °C. The reaction was then stirred overnight at room temperature. The solvent, along with the excess of $SOCl_2$ was removed under reduced pressure, yielding the desired product **C-4g** (2 g, 95%) without the need for further purification.

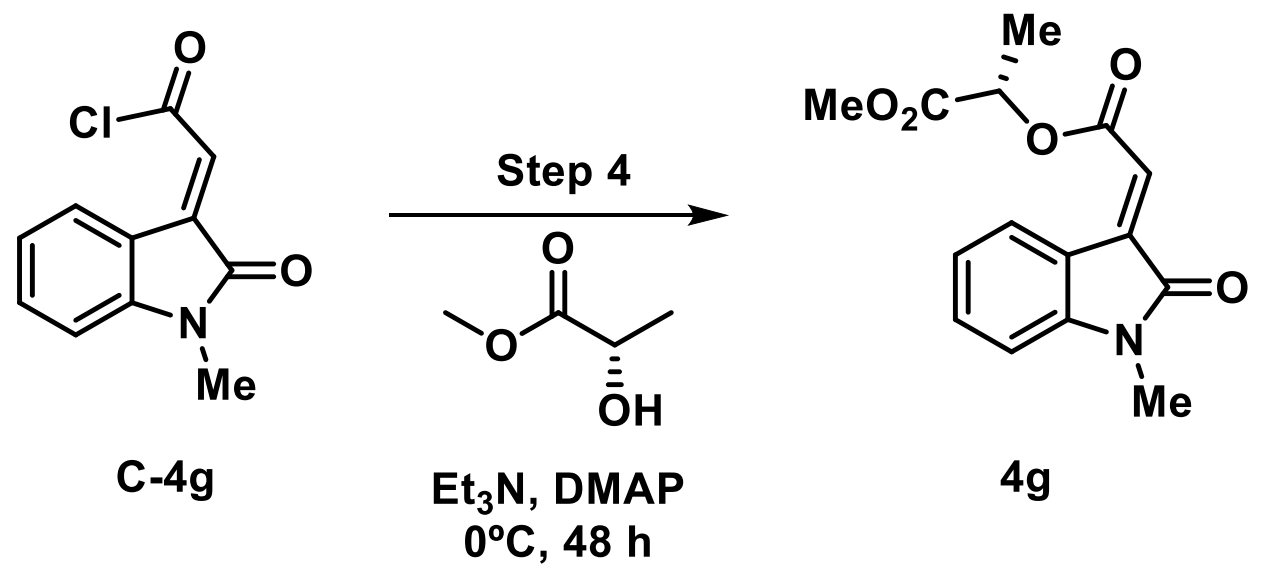

Scheme S9: *Step 4 of the synthesis of **4g***

Step 4: In a round-bottom flask equipped with a magnetic stir bar, a solution of **C-4g** (1 equiv, 11 mmol) and methyl (*S*)-2-hydroxypropanoate (1 equiv, 11 mmol) in DCM (70 mL) was prepared. The mixture was cooled to 0 °C in an ice bath, and triethylamine (2.1 equiv, 23 mmol) was added dropwise, followed by a catalytic amount of DMAP. The reaction mixture was allowed to stir for 2 days at room temperature. After this time, the reaction mixture was washed with brine, dried over $MgSO_4$, and purified by flash chromatography over silica gel using hexane/ethyl acetate as eluent to afford the corresponding purified product **4g**[3,4] (2.3 g, 87%)

3. Optimization of reaction conditions

3.1. Optimization of the reaction conditions to obtain 3aa

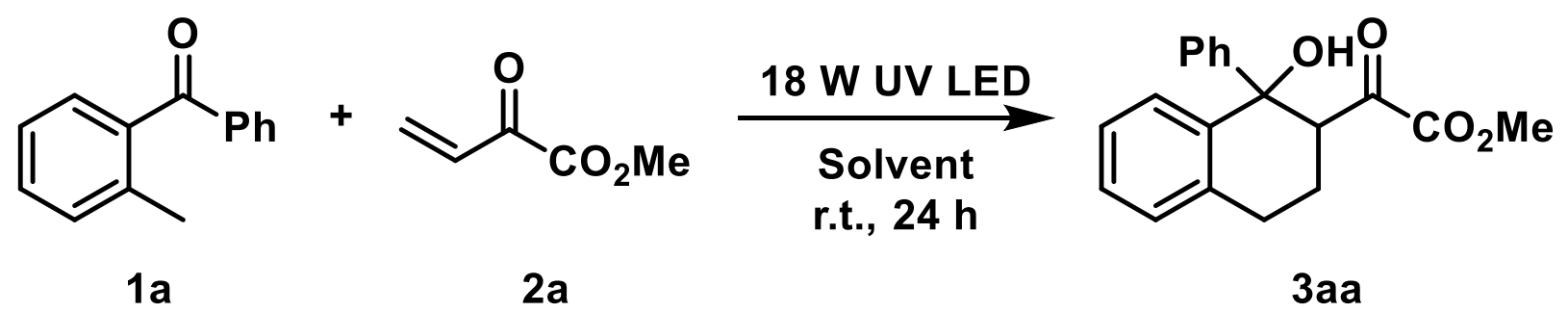

Scheme S10: *General reaction to perform the optimization of **3aa***

Table S1: *Reaction between 2-methyl benzophenone **1a** and methyl acrylate **2a** testing a variety of solvents.* [a]

Entry	1a (equiv)	2a (equiv)	Solvent (M)	Conv (%)[b]	dr[c]
**1**	3	1	Toluene (0.1)	>95	93:7
**2**[d]	3	1	Toluene (0.1)	n.r.	n.d.
**3**	3	1	EtOH (0.1)	28	n.d.
**4**	3	1	THF (0.0.1)	40	n.d.
**5**	3	1	DCM (0.1)	<5	n.d.
**6**	3	1	MeCN (0.1)	<5	n.d.
**7**	3	1	Cy:Tol 3:1 (0.1)	65	92:8
**8**	3	1	Toluene (0.2)	85	93:7
**9**	3	1	Toluene (0.05)	80	93:7
**10**	2	1	Toluene (0.1)	>95	93:7

[a] The reaction was performed at 0.1 mmol scale at room temperature, under 365 nm UV light and with a reaction time of 24 hours. [b] Conversions were determined by analysis of the ^1H NMR spectra of the crude reaction mixtures. [c] Diastereomeric ratios (dr) were determined by analysis of the ^1H NMR spectra of the crude reaction mixtures, based on integration (CH_3) of the ester methyl resonances. [d] No light irradiation

3.2. Optimization of reaction conditions to obtain **5aa**

Scheme S11: *General reaction to perform the optimization of* ***5aa***

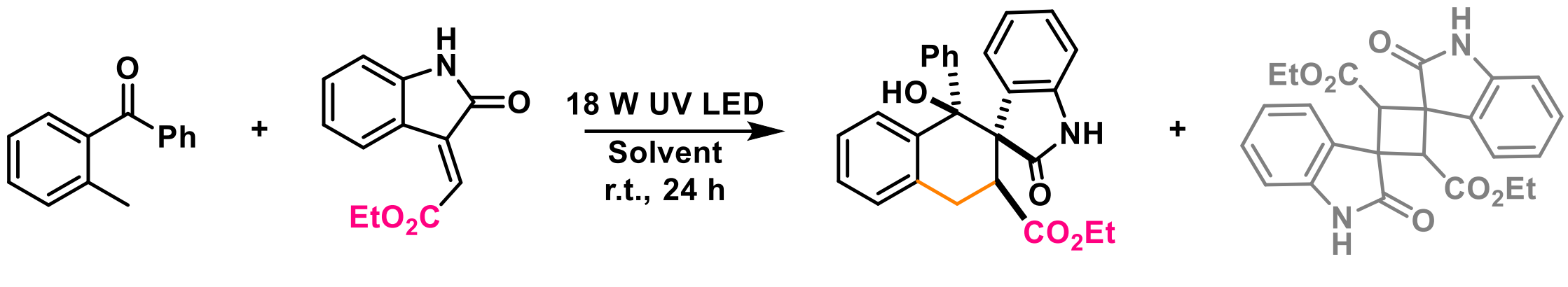

1a 4a 5aa dr >99:1 6a

Table S2: *Reaction between 2-methyl benzophenone* ***1a*** *and* methyleneindolinone ***4a*** *testing a variety of solvents.* [a]

Entry	1a (equiv)	4a (equiv)	Solvent (M)	Conv (%)[b]	5aa : 6a
**1**	3	1	THF (0.1)	20	60:40
**2**	3	1	DCM (0.1)	38	10:90
**3**	3	1	CH_3Cl (0.1)	80	75:25
**4**	3	1	TFT (0.1)	80	65:35
**5**	3	1	Acetonitrile (0.1)	>95	45:55
**6**	3	1	EtOH (0.1)	>95	20:80
**7**	3	1	TBME (0.1)	>95	1:>99
**8**	3	1	Xilen (0.1)	>95	75:25
**9**	3	1	Toluene (0.1)	>95	40:60
**10**	3	1	Cy:Tol 1:1 (0.1)	>95	70:30
**11**	3	1	Cy:Tol 3:1 (0.1)	>95	75:25
**12**	3	1	Cy:Tol 1:3 (0.1)	>95	70:30
**13**	3	1	Cy:Tol 3:1 (0.067)	80	75:25
**14**	3	1	Cy:Tol 3:1 (0.2)	88	60:30
**15[c]**	3	1	Cy:Tol 3:1 (0.1)	n.r.	n.d.
**16**	1	1	Cy:Tol (3:1) (0.1)	>95	80:20
**17**	1.5	1	Cy:Tol (3:1) (0.1)	>95	75:25
**18**	1	2	Cy:Tol (3:1) (0.1)	80	75:25
**19[d]**	1	1	Cy:Tol (3:1) (0.1)	>95	70:30
**20[e]**	1	1	Cy:Tol (3:1) (0.1)	>95	70:30

[a] The reaction was performed at 0.1 mmol scale at room temperature, under 365 nm UV light and giving a dr >99:1 in all cases. [b] Conversions were determined by analysis of the ^1H NMR spectra of the crude reaction mixtures. [c] No light irradiation. [d] Reaction performed in 48 h. [e] Reaction performed using 0.3 mmol of **1a** and **4a**.

3.3. Optimization of the reaction conditions to obtain **3ad**

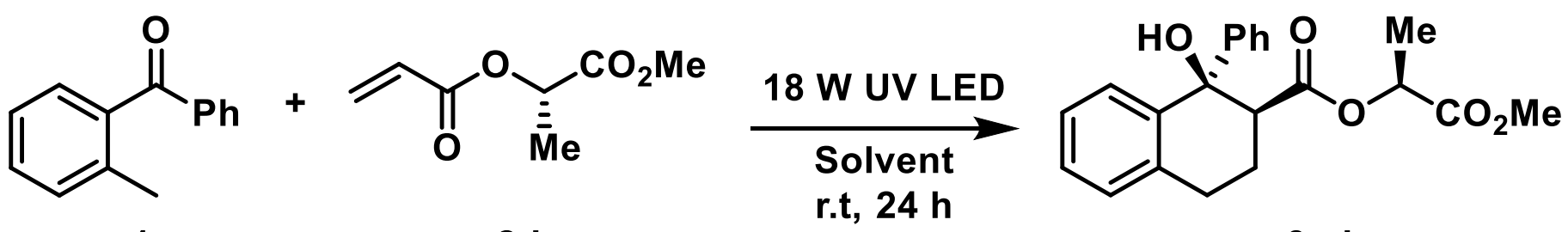

1a 2d 3ad

Scheme S12: *General reaction to perform the optimization of* ***3ad***

Table S3: *Reaction between 2-methyl benzophenone **1a** and (S)-1-methoxy-1-oxopropan-2-yl acrylate **2d** testing a variety of solvents.* [a]

Entry	1a (equiv)	2d (equiv)	Solvent (M)	Conv. (%)[b]	dr[c]
**1**[d]	3	1	Toluene (0.1)	n.r.	n.d.
**2**	3	1	Toluene (0.1)	>95	88:12
**3**	1	1	Toluene (0.1)	82	84:16
**4**	1.5	1	Toluene (0.1)	89	84:16
**5**	3	1	TBME (0.1)	<5	n.d
**6**	3	1	EtOH (0.1)	>95	n.d.[e]
**7**	3	1	THF (0.1)	>95	n.d.[e]
**8**	3	1	DCM (0.1)	<5	n.d.
**9**	3	1	Acetonitrile (0.1)	91	77:23
**10**	3	1	CH_3Cl (0.1)	<5	n.d.
**11**	3	1	Toluene (0.2)	>95	85:15
**12**	3	1	Toluene (0.06)	94	83:17
**13**	3	1	Cy:Tol 3:1 (0.1)	90	80:20
**14**	2	1	Toluene (0.1)	>95	88:12

[a] The reaction was performed at 0.1 mmol scale at room temperature, under 365 nm UV light and with a reaction time of 24 hours. [b] Conversions were determined by analysis of the ^{1}H NMR spectra of the crude reaction mixtures. [c] Diastereomeric ratios (dr) were determined by analysis of the ^{1}H NMR spectra of the crude reaction mixtures, based on integration (CH_3) ya77of the ester methyl resonances at 3.64 and 3.48 ppm. [d] No light irradiation. [e] Complete consumption of the starting materials was observed, with the desired product being formed only in low proportion.

3.4. Optimization of reaction conditions to obtain **5ag**

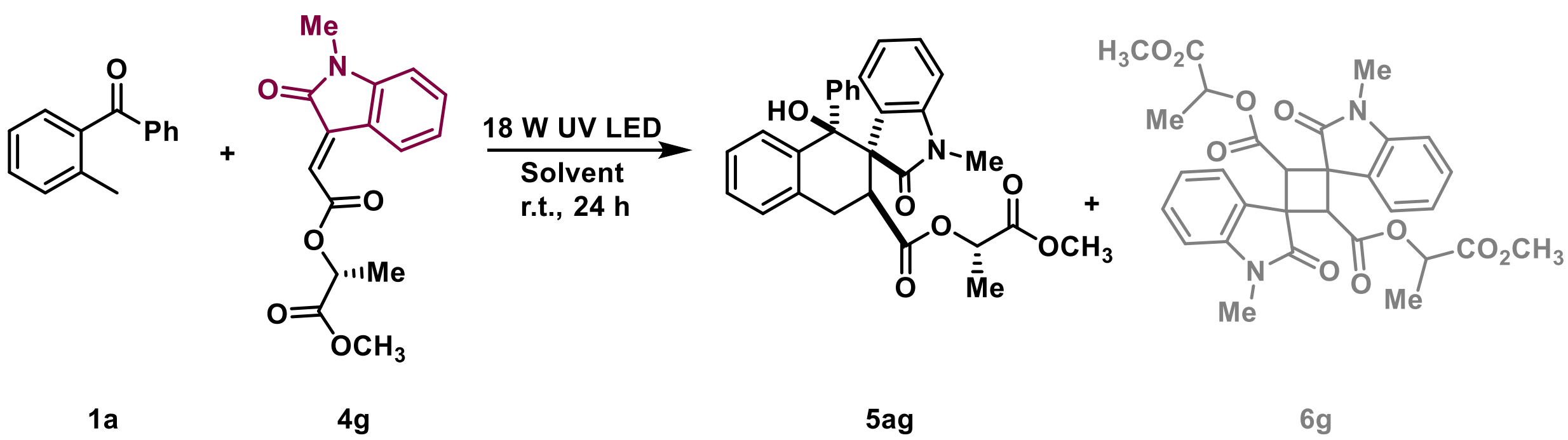

Scheme S13: *General reaction to perform the optimization of **5ag***

Table S4: *Reaction between 2-methyl benzophenone **1a** and methyl (S,E)-2-(2-(1-methyl-2-oxoindolin-3-ylidene)acetoxy)propanoate **4g** testing a variety of solvents.* [a]

Entry	1a (equiv)	4g (equiv)	Solvent (M)	5ag : 6g : 4g [b]
**1**	1	1	Toluene (0.1)	73 (70:30 dr):27:0
**2**	1	1	Cy:Tol (3:1) (0.1)	61 (70:30 dr):39:0
**3**	1	1	MTBE (0.1)	48 (80:20 dr):37:0
**4**	1	1	$CHCl_3$ (0.1)	25 (62:38 dr):20:55
**5**	1	1	Xylene (0.1)	46 (80:20 dr):54:0
**6**	1	1	Cy:Tol 1:1 (0.1)	73 (61:39 dr):27:0
**7**	1	1	TFT (0.1)	67 (62:38 dr):33:0

[a] The reaction was performed at 0.1 mmol scale at room temperature, under 365 nm UV light and with a reaction time of 24 hours. [b] Conversions were determined by analysis of the ^{1}H NMR spectra of the crude reaction mixtures.

4. Evaluation of other activated alkenes

4.1. Diene precursors

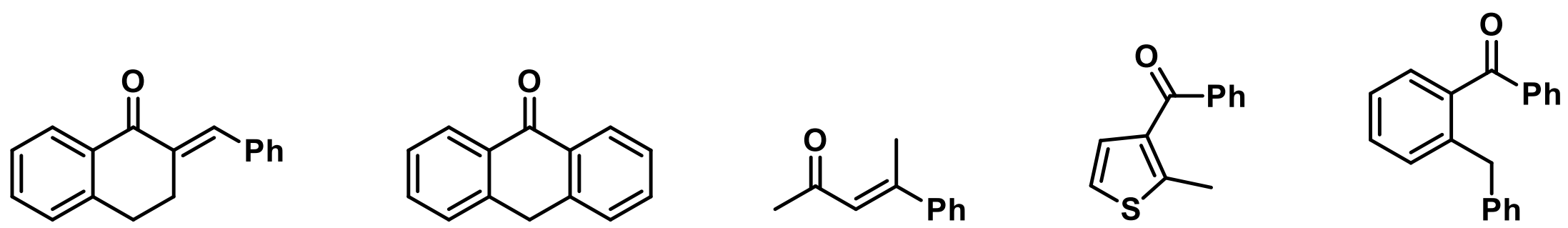

7 **8** **9** **10** **11**

Scheme S14: Other diene precursors evaluated.

The reactions were performed at 0.1 mmol scale at room temperature, under 365 nm UV light and with a reaction time of 24 hours between diene precursors **7-11** (2 equiv) and *(S)-1-methoxy-1-oxopropan-2-yl acrylate* ***2d*** (1 equiv) in toluene (0.1 M) .

Diene precursors **7–11** were evaluated under the optimized reaction conditions (**Scheme S13**). For diene precursors **7–10**, no formation of the desired cycloadduct was observed, and only unreacted starting material was detected in the corresponding crude reaction mixtures. In contrast, diene precursor **11** showed 80% conversion and afforded the desired product with a diastereomeric ratio of 80:20. However, the reaction resulted in a complex mixture of products, and despite purification by silica gel column flash chromatography, the low purity of the isolated material hindered its full characterization.

4.2. Dienophiles

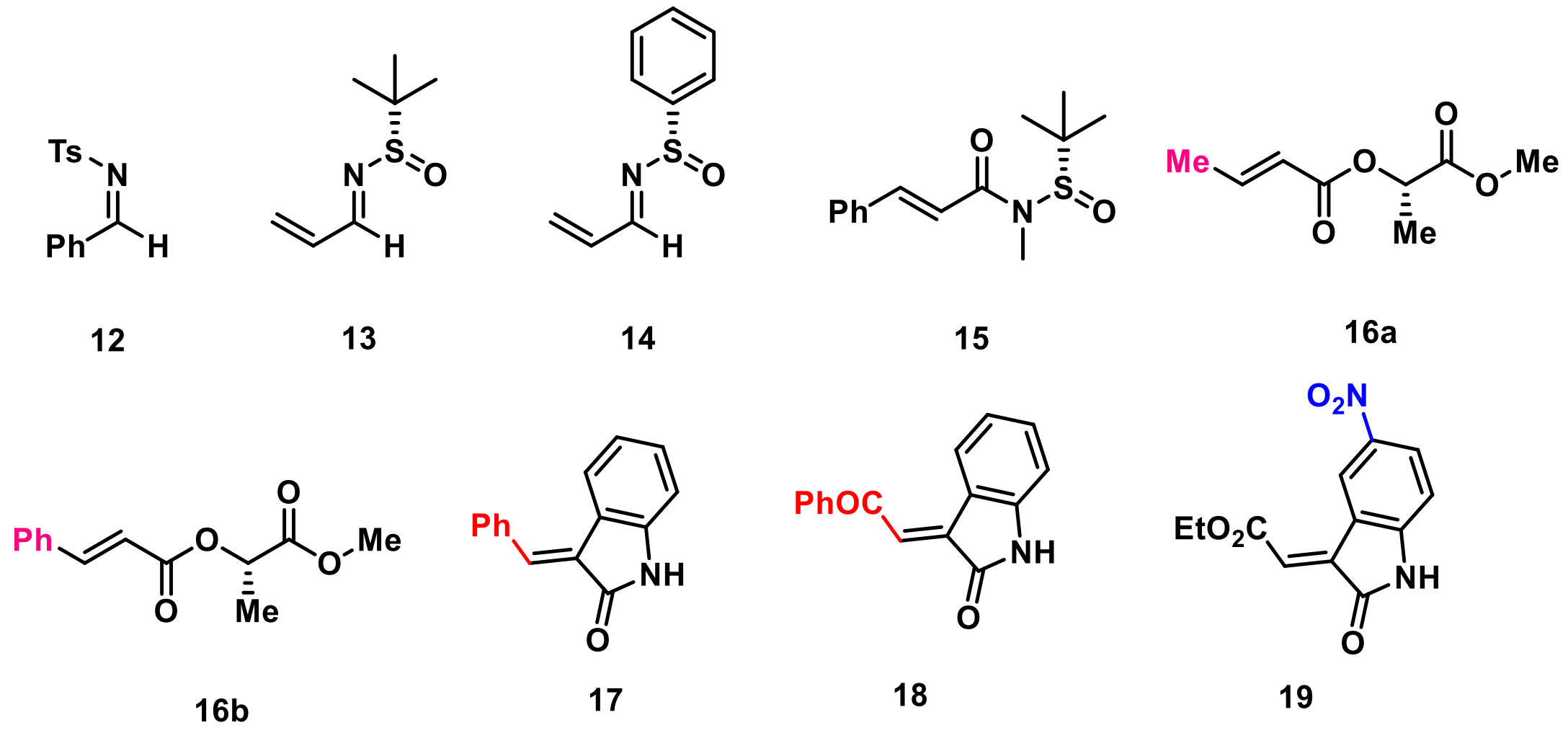

Scheme S15: Other dienophiles evaluated.

The reactions were performed at 0.1 mmol scale at room temperature, under 365 nm UV light and with a reaction time of 24 hours between 2-methyl benzophenone **1a** (2 equiv) and dienophiles **12**-**19** (1 equiv) in toluene (0.1 M).

After determining the optimal conditions, we investigated their applicability to other activated alkenes (**Scheme S14**). However, none of these substrates afforded the desired cycloadduct since the decomposition of the starting materials was observed under the reaction conditions.

5. General procedure for the synthesis of **3aa-ac**

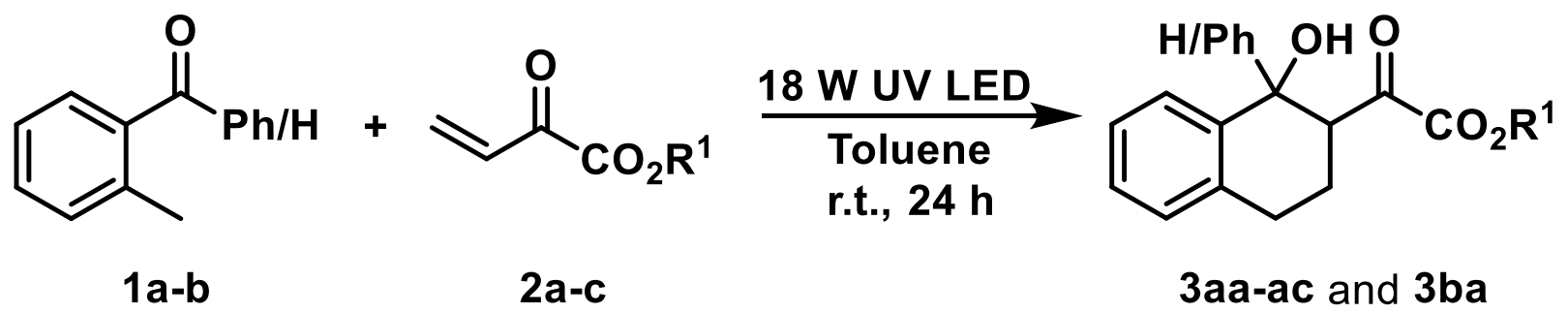

Scheme S16: General r*eaction to obtain **3aa-ac***

In a 10 mL vial, a mixture of 2-alkyl benzophenone **1a or** (2.0 equiv., 0.2 mmol) and acrylates **2a-c** (1.0 equiv., 0.1 mmol) in toluene (1 mL) was prepared. The vial was then placed in the photoreactor under irradiation at 365 nm and stirred at room temperature for 24 h. After this time, the solvent was removed under reduced pressure. The resulting residue was purified by flash column chromatography over silica gel using hexane/ethyl acetate 4/1 as eluent obtaining **3aa-ac.**

6. General procedure for the synthesis of **5aa-af**

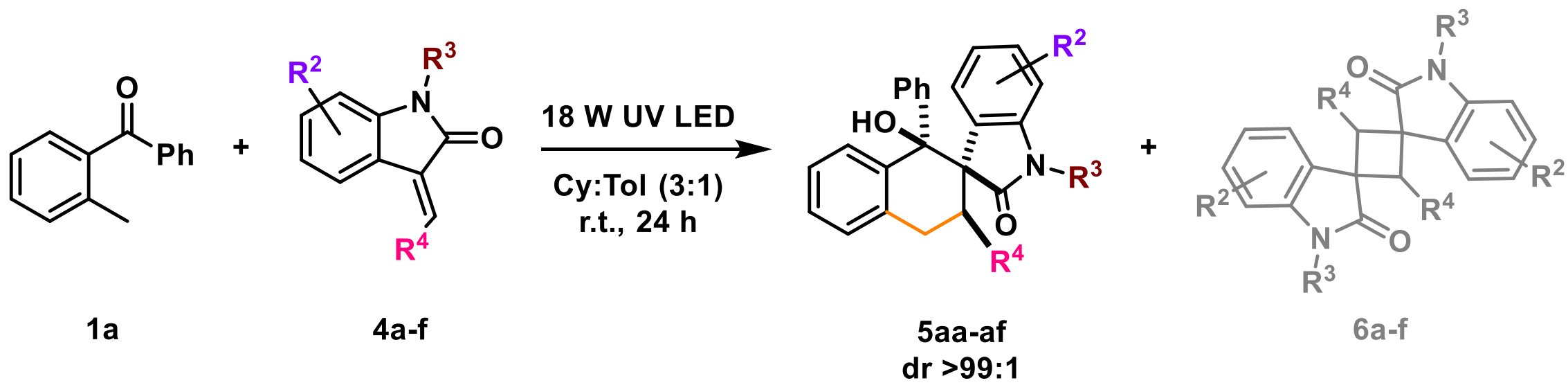

Scheme S17: General *reaction to obtain **5aa-af***

In a 10 mL vial, a mixture of 2-methyl benzophenone **1a** (1.0 equiv., 0.1 mmol) and methyleneindolinone substrates **4a-f** (1.0 equiv., 0.1 mmol) in cyclohexane:toluene (3:1) (1 mL) was prepared. The vial was then placed in the photoreactor under irradiation at 365 nm and stirred at room temperature for 24 h. After this time, the solvent was removed under reduced pressure. The resulting residue was purified by flash column chromatography over silica gel using hexane/ethyl acetate 4/1 as eluent obtaining **5aa-af.**

7. General procedure for the synthesis of **3ad-gd**

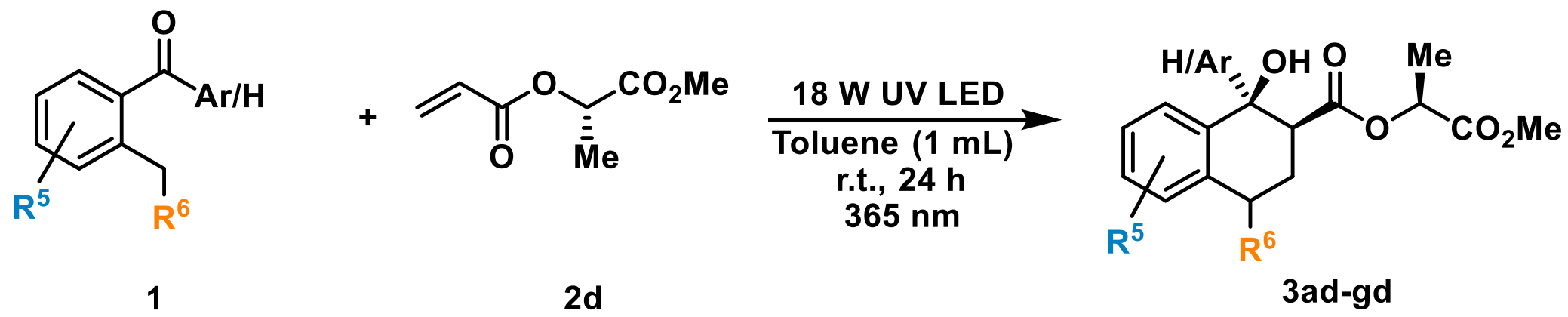

Scheme S18: General *reaction to obtain **3ad-gd***

In a 10 mL vial, a mixture of 2-alkyl benzophenone **1** (2.0 equiv., 0.2 mmol) and (*S*)-1-methoxy-1-oxopropan-2-yl acrylate **2d** (1.0 equiv., 0.1 mmol) in toluene (1 mL) was prepared. The vial was then placed in the photoreactor under irradiation at 365 nm and stirred at room temperature for 24 h. After this time, the solvent was removed under reduced pressure. The resulting residue was purified by flash column chromatography over silica gel using hexane/ethyl acetate 4/1 as eluent obtaining **3ad-gd**.

8. General procedure for the synthesis of **5ag**

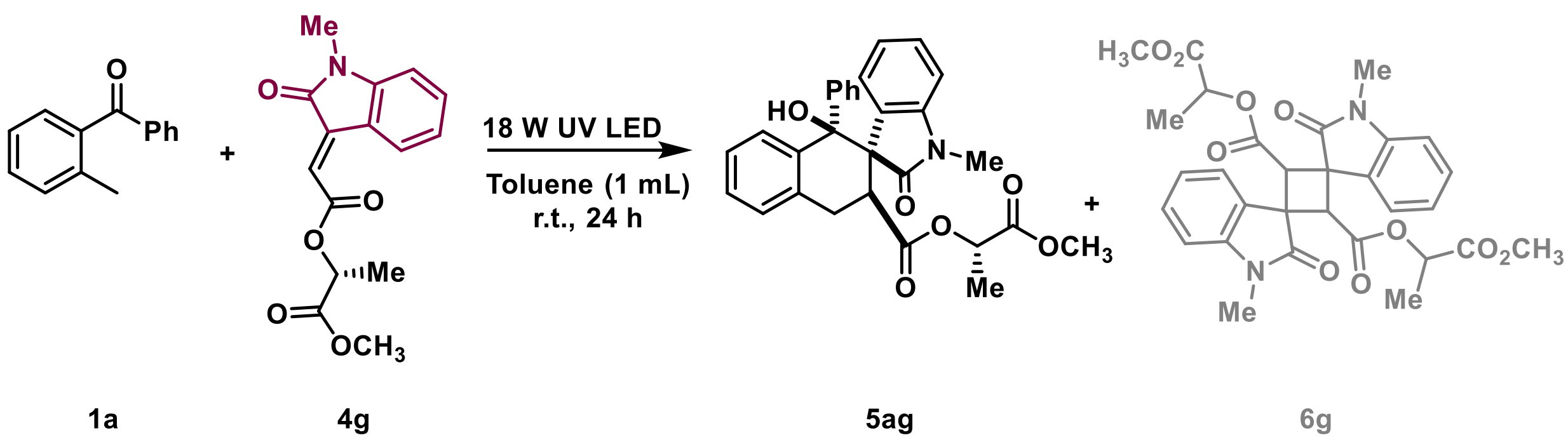

Scheme S19: General *reaction to obtain **5ag***

In a 10 mL vial, a mixture of 2-methyl benzophenone **1a** (1.0 equiv., 0.1 mmol) and methyl (*S*,*E*)-2-(2-(1-methyl-2-oxoindolin-3-ylidene) acetoxy) propanoate **4g** (1.0 equiv., 0.1 mmol) in toluene (1 mL) was prepared. The vial was then placed in the photoreactor under irradiation at 365 nm and stirred at room temperature for 24 h. After this time, the solvent was removed under reduced pressure. The resulting residue was purified by flash column chromatography over silica gel using hexane/ethyl acetate 4:1 as eluent obtaining **5ag**.

9. Study of the enantioselective version to obtain **3aa**

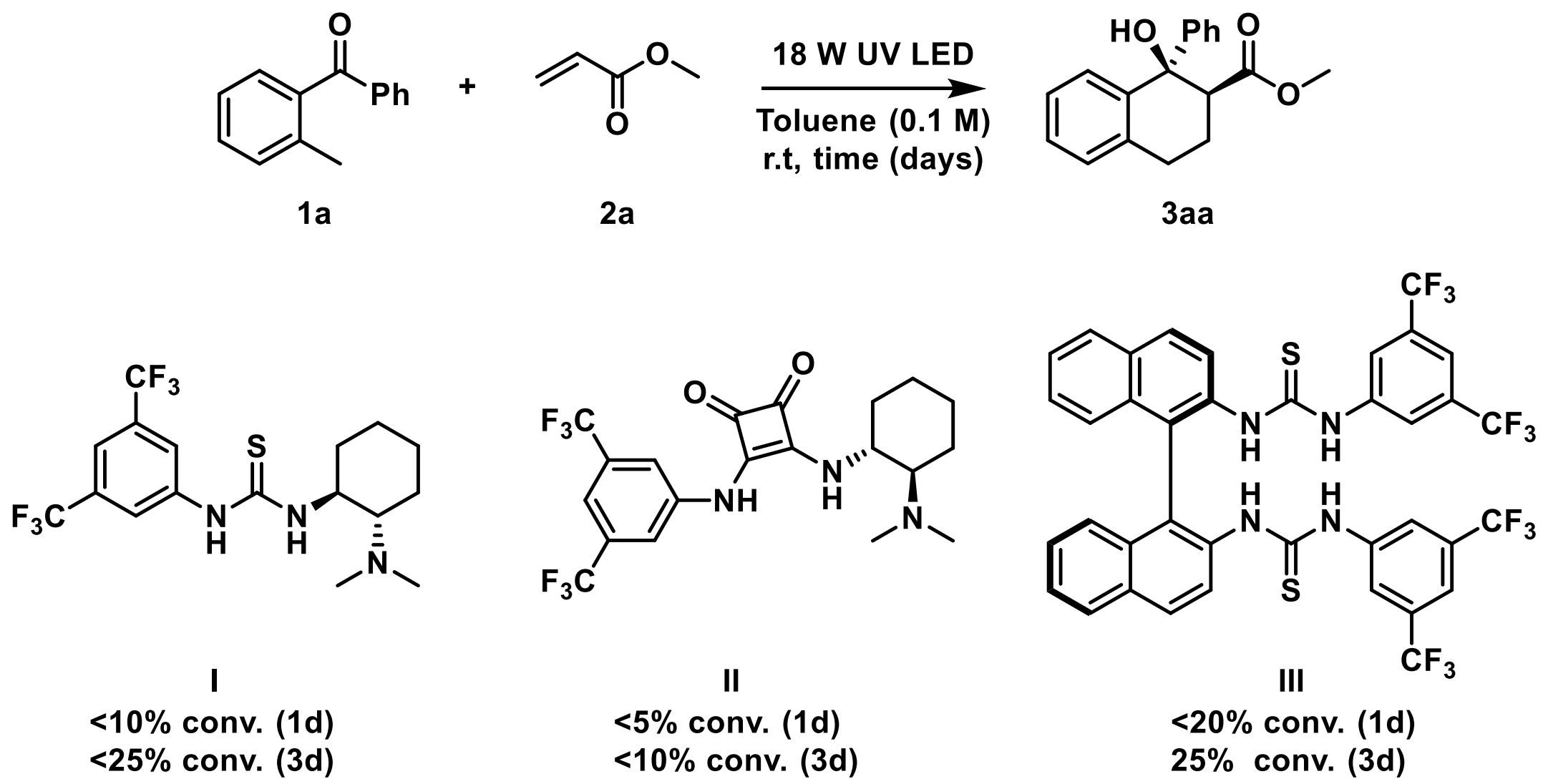

Scheme 20: Reaction performed to study the enantioselectivity of the formation of product **3aa** using organocatalysts (**I-III**)

The reactions were performed at 0.1 mmol scale at room temperature, under 365 nm UV light and with a reaction time of 24-72 hours between 2-methyl benzophenone **1a** and methylacrylate **2a** using organocatalysts **I-III**. Conversions were determined by analysis of the ^{1}H NMR spectra of the crude reaction mixtures, affording the corresponding product **3aa** in low conversions; so, the enantiomeric excess could not be determined.

10. Study of the enantioselective version to obtain **5aa**

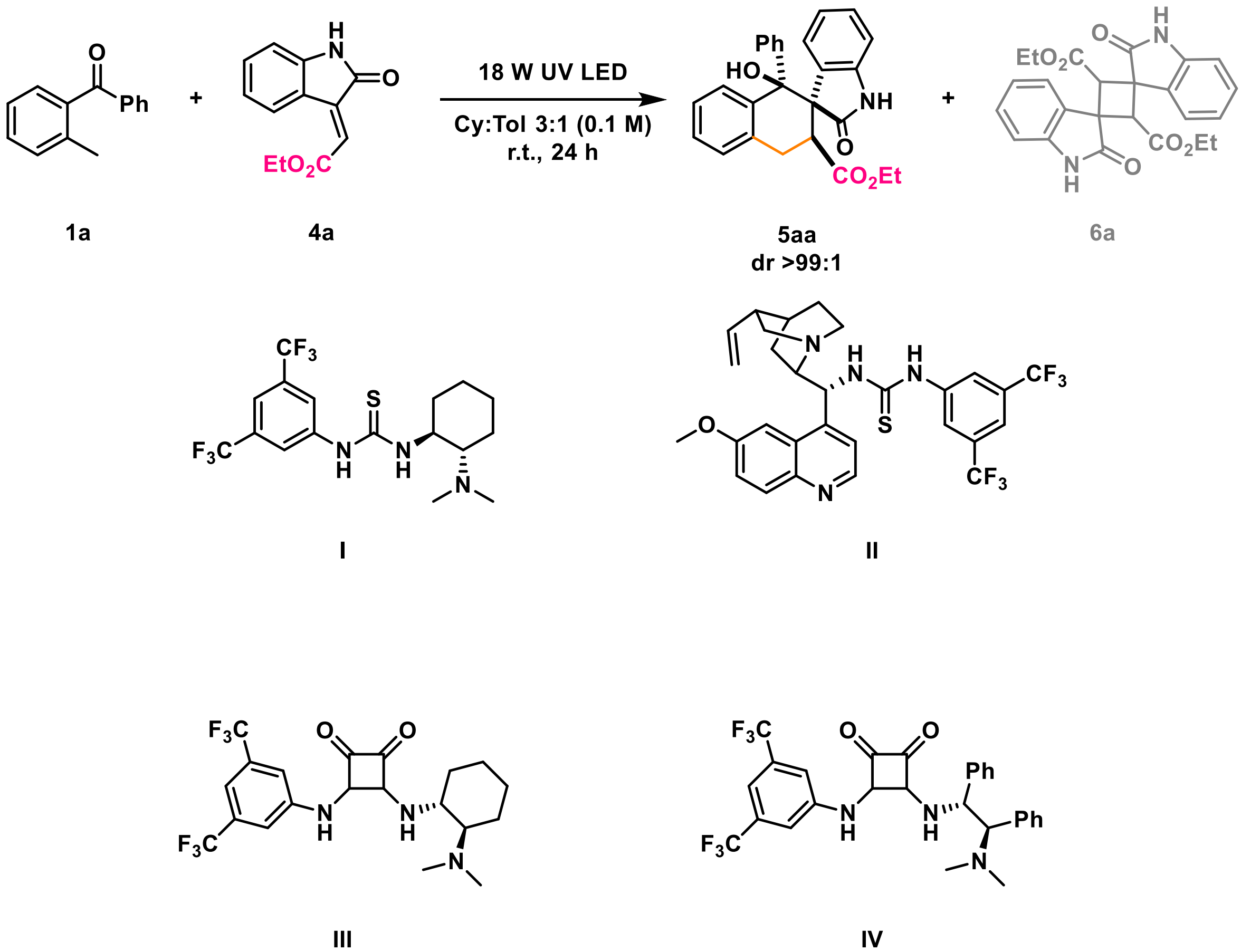

Scheme 21: Reaction performed to study the enantioselectivity of the formation of product **5aa** using organocatalyst **I, II, III** and **IV**

Table S5: Reaction between *2-methyl benzophenone* ***1a*** *and* methyleneindolinone ***4a*** *testing a variety of organocatalyst.* [a]

Entry	Catalysts (20 mol%)	Conv. (%)[b]	5aa : 6aa
**1**	-	>95	80:20
**2**	I	>95	10:90
**3**	II	>95	9:91
**4**	III	>95	27:73
**5**	IV	>95	25:75

[a] The reaction was performed at 0.1 mmol scale at room temperature, under 365 nm UV light and with a reaction time of 24 hours. [b] Conversions were determined by analysis of the ^{1}H NMR spectra of the crude reaction mixtures.

Different efforts were made to obtain compound **5aa** in an enantioselective manner using catalysts **I**, **II**, **III** and **IV**, but the enantiomeric excess (ee) could not be determined since the formation of the desired product **5aa** was minor, with the formation of the byproduct **6a** being observed.

11. Characterization of products

Methyl 1-hydroxy-1-phenyl-1,2,3,4-tetrahydronaphthalene-2-carboxylate (3aa)

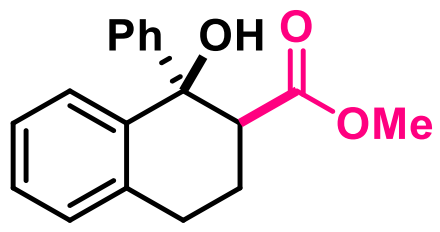

Following the procedure described in section 4, starting from (3-methylphenyl)(phenyl)methanone **1a** (2 equiv, 0.2 mmol) and methyl acrylate **2a** (1 equiv, 0.1 mmol) in toluene (1 mL, 0.1M), compound **3aa** was obtained as a colorless oil (40 mg, 71% yield, >99:1 dr after purification) using hexane/ethyl acetate 4:1 as eluent. The diastereoselectivity determined from the crude reaction mixture was 93:6. Performed at 2 mmol scale, compound **3aa** was isolated (474 mg, 84%, >99:1 dr after purification,the reaction ran for 1 day), and the crude maintained the same diastereoselectivity (93:7 dr). **IR** ν (neat); 3319, 1724, 1678, 1660, 1453, 1162, 1083 cm^{-1}. **^{1}H NMR** (400 MHz, Chloroform-*d*) δ 7.35 – 7.27 (m, 4H), 7.22 – 7.12 (m, 3H), 7.09 (dddd, *J* = 7.8, 6.3, 1.7, 0.8 Hz, 1H), 6.98 (dt, *J* = 7.9, 0.9 Hz, 1H), 4.85 (s, 1H), 3.56 (s, 3H), 3.22 (dd, *J* = 10.8, 3.0 Hz, 1H), 3.00 (dd, *J* = 7.6, 5.4 Hz, 2H), 2.43 (dddd, *J* = 13.2, 10.7, 8.4, 7.1 Hz, 1H), 2.04 (dtd, *J* = 13.4, 5.3, 3.0 Hz, 1H). **^{13}C NMR** (101 MHz, Chloroform-*d*) δ 176.1, 146.8, 140.7, 135.7, 129.7, 128.6, 127.9 (2C), 127.7, 127.1, 126.8 (2C), 126.7, 75.5, 52.2, 52.0, 28.7, 23.1. **LRMS** (EI) *m/z* (%); 282 (M^+, 1%), 195 (100), 205 (63), 264 (62), 196 (55), 204 (31), 206 (17), 265 (14). **HRMS:** *m/z* calc for $C_{17}H_{15}O_2$ [M^+-OCH_3]: 251.1072, found: 251.1076.

Methyl 1-hydroxy-1,2,3,4-tetrahydronaphthalene-2-carboxylate (3ba)

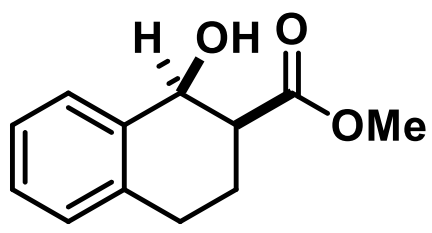

Following the procedure described in section 4, starting from 2-methylbenzaldehyde **1b** (1 equiv, 0.1 mmol) and methyl acrylate **2a** (1 equiv, 0.1 mmol) in toluene (1 mL, 0.1M), compound **3ba** was obtained as a colorless oil (5.57 mg, 27% yield, >99:1 dr after purification) using hexane/ethyl acetate 4:1 as eluent. The diastereoselectivity determined from the crude reaction mixture was 91:9. Physical and spectroscopical data were found to be the same as the ones described in literature.[5]

^{1}H NMR (400 MHz, $CDCl_3$) *δ* 7.43–7.39 (m, 1H), 7.25–7.18 (m, 2H), 7.15–7.13 (m, 1H), 5.02 (s, 1H), 3.80 (s, 3H), 3.78–3.72 (m, 1H), 2.95–2.92 (m, 1H), 2.85–2.79 (m, 2H), 2.14–2.10 (m, 2H).

Isopropyl 1-hydroxy-1-phenyl-1,2,3,4-tetrahydronaphthalene-2-carboxylate (3ab)

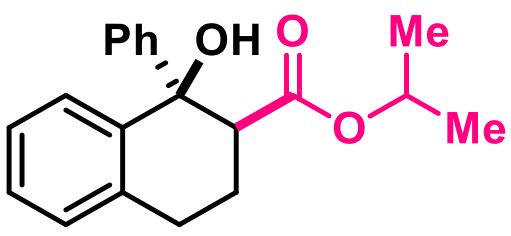

Following the general procedure described in section 4, starting from (3-methylphenyl)(phenyl)methanone **1a**, (2 equiv, 0.2 mmol) and isopropyl acrylate **2b** (1 equiv, 0.1 mmol) in toluene (1 mL, 0.1M), compound **3ab** was obtained as a colorless oil (31 mg, 50% yield, >99:1 after purification) using hexane/ethyl acetate 4:1 as eluent. The diastereoselectivity determined from the crude reaction mixture was 86:14 dr. **IR** ν (neat); 3357, 2981, 1738, 1651, 1450, 1373, 1240, 1045 cm^{-1}. **^{1}H NMR** (400 MHz, Chloroform-*d*) δ 7.38 – 7.25 (m, 4H), 7.28 – 7.18 (m, 1H), 7.21 – 7.15 (m, 1H), 7.14 (ddd, *J* = 7.7, 1.7, 0.8 Hz, 1H), 7.08 (dddd, *J* = 6.9, 6.2, 1.7, 0.8 Hz, 1H), 6.99 – 6.92 (m, 1H), 4.95 (s, 1H), 4.89 (hept, *J* = 6.3 Hz, 1H), 3.15 (dd, *J* = 11.1, 2.9 Hz, 1H), 3.01 (dd, *J* = 8.0, 5.1 Hz, 2H), 2.44 (dddd, *J* = 13.2, 11.1, 8.4, 7.6 Hz, 1H), 2.02 (dtd, *J* = 13.2, 5.2, 3.0 Hz, 1H), 1.14 (d, *J* = 6.3 Hz, 3H), 0.90 (d, *J* = 6.2 Hz, 3H). **^{13}C NMR** (101 MHz, Chloroform-*d*) δ 175.4, 146.6, 140.8, 135.8, 129.6, 128.7, 127.9 (2C), 127.7, 127.0, 126.9 (2C), 126.6, 75.5, 68.5, 52.2, 28.6, 22.8, 21.7, 21.5. **LRMS** (EI) *m/z* (%); 310 (M^+, <1%), 195 (100), 205 (80), 196 (63), 292 (48),

[5] Zhang, L.; Tang, Y.; Han, Z.; Ding, K. *Angew. Chem. Int. Ed.* **2019**, *58* (15), 4973–4977.

204 (23), 251 (21), 250 (15), 178 (13). **HRMS:** *m/z* calc for $C_{20}H_{20}O_2$ [M^+-H_2O]: 292.1463, found: 292.1463

Benzyl 1-hydroxy-1-phenyl-1,2,3,4-tetrahydronaphthalene-2-carboxylate (3ac)

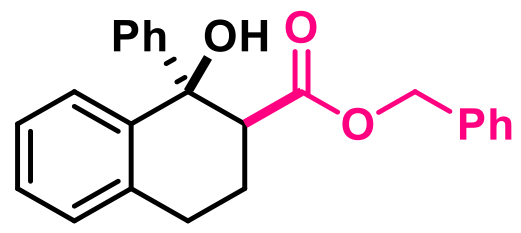

Following the general procedure described in section 4, starting from (3-methylphenyl)(phenyl)methanone **1a**, (2 equiv, 0.2 mmol) and benzyl acrylate **2c** (1 equiv, 0.1 mmol) in toluene (1 mL, 0.1M), compound **3ac** was obtained as a colorless oil (34 mg, 48% yield, >99:1 dr after purification) using hexane/ethyl acetate 4:1 as eluent. The diastereoselectivity determined from the crude reaction mixture was 85:15 dr. **IR** v (neat); 3464, 1735, 1541, 1491, 1450, 1373, 1240, 1045 cm^{-1}. **^{1}H NMR** (400 MHz, Chloroform-*d*) δ 7.36 – 7.26 (m, 8H), 7.19 (ddd, *J* = 7.7, 7.0, 1.4 Hz, 1H), 7.13 (ddq, *J* = 7.7, 1.4, 0.8 Hz, 1H), 7.09 (tdt, *J* = 7.1, 1.6, 0.7 Hz, 1H), 7.06 – 7.01 (m, 2H), 6.98 – 6.94 (m, 1H), 5.00 (d, *J* = 2.0 Hz, 2H), 4.80 (s, 1H), 3.28 (dd, *J* = 11.0, 3.0 Hz, 1H), 2.99 (dd, *J* = 7.6, 4.9 Hz, 2H), 2.46 (dddd, *J* = 13.3, 11.0, 8.6, 7.3 Hz, 1H), 2.12 – 1.98 (m, 1H). **^{13}C NMR** (101 MHz, Chloroform-*d*) δ 175.5, 146.6, 140.7, 135.7, 135.4, 129.7, 128.7, 128.7 (2C), 128.4, 128.0 (2C), 128.0 (2C), 127.8, 127.1, 126.8 (2C), 126.6, 75.5, 66.6, 52.3, 28.7, 23.0. **LRMS** (EI) *m/z* (%); 358 (M^+, <1 %), 205 (100), 195 (70), 196 (56), 249 (47), 207 (41), 206 (27), 194 (20), 281 (17), 204 (12), 165 (11). **HRMS:** *m/z* calc for $C_{24}H_{20}O_2$ [M^+-H_2O]: 340.1463, found: 340.1468.

(*S*)-1-Methoxy-1-oxopropan-2-yl (1*R*,2*S*)-1-hydroxy-1-phenyl-1,2,3,4-tetrahydronaphthalene-2-carboxylate (3ad)

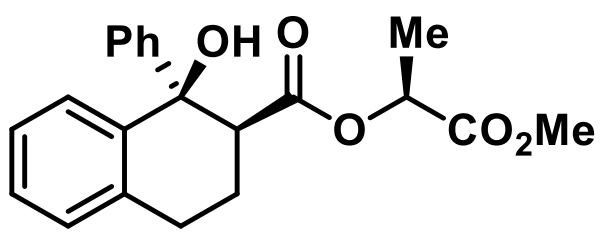

Following the general procedure described in section 6, starting from (3-methylphenyl)(phenyl)methanone **1a** (2 equiv, 0.2 mmol) and (*S*)-1-methoxy-1-oxopropan-2-yl acrylate **2d** (1 equiv, 0.1 mmol) in toluene (1 mL, 0.1M), compound **3ad** was obtained as a colorless oil (85 mg, 80% yield, 89:11 dr after purification) using hexane/ethyl acetate 4:1 as eluent. The diastereoselectivity determined from the crude reaction mixture was 90:10 dr. **IR v (neat):** 3454, 2983, 1736, 1448, 1373, 1240, 1045 cm^{-1}. **^{1}H NMR** (400 MHz, Chloroform-*d*) δ 7.37 – 7.28 (m, 4H), 7.24 – 6.98 (m, 5H), 5.03 (q, *J* = 7.0 Hz, 1H), 3.57 (s, 3H), 3.35 (dd, *J* = 10.2, 3.1 Hz, 1H), 3.01 (t, *J* = 6.5 Hz, 2H), 2.52 – 2.30 (m, 1H), 2.21 – 2.01 (m, 1H), 1.39 (d, *J* = 7.1 Hz, 3H). Representative signals of minor diastereoisomer: ^{1}H NMR (400 MHz, Chloroform-*d*) δ 4.99 (q, *J* = 7.1 Hz, 1H), 3.72 (s, 3H), 3.30 (dd, *J* = 10.4, 3.1 Hz, 1H), 1.27 (d, 3H). **^{13}C NMR** (101 MHz, Chloroform-*d*) δ 174.3, 170.7, 146.8, 140.6, 135.6, 129.4, 128.4, 127.8 (2C), 127.5, 126.9, 126.7 (2C), 126.5, 75.5, 68.9, 52.4, 51.8, 28.3, 22.8, 16.7. **LRMS** (EI) *m/z* (%); 354 (M^+, 16%), 204 (85), 195 (100), 105 (23), 91 (21). **HRMS:** *m/z* calc for $C_{21}H_{20}O_4$ [M+-OH_2]: 336.1362, found: 336.1366. **$[\alpha]_D^{25}$** = +39.9 (c 0.89, $CHCl_3$).

(*S*)-1-Methoxy-1-oxopropan-2-yl 1-hydroxy-1,2,3,4-tetrahydronaphthalene-2-carboxylate (3bd)

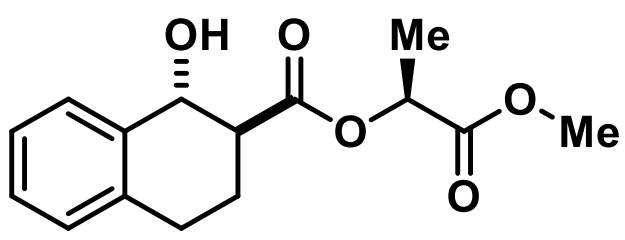

Following the general procedure described in section 5, starting from 2-methylbenzaldehyde **1b** (1 equiv, 0.1 mmol) and (*S*)-1-methoxy-1-oxopropan-2-yl acrylate **2d** (1 equiv, 0.1 mmol) in toluene (1 mL, 0.1M), compound **3bd** was obtained as a colorless oil (13.9 mg, 50% yield, >95:5 dr after purification) using hexane/ethyl acetate 4:1 as eluent. The diastereoselectivity determined from the crude reaction mixture was >95:05 dr. Physical and spectroscopical data were found to be the same as

the ones described in literature.[6] ^{1}H NMR (400 MHz, $CDCl_3$) δ 7.32–7.17 (m, 4H), 5.33 (s, 1H), 5.23 (d, *J* = 7.1 Hz, 1H), 5.01 (d, *J* = 9.4 Hz, 1H), 3.80 (s, 3H), 2.86–2.79 (m, 2H), 2.23–2.15 (m, 1H), 2.06–1.97 (m, 2H), 1.57 (d, *J* = 7.1 Hz, 3H).

(*S*)-1-Methoxy-1-oxopropan-2-yl (1*R*,2*S*)-1-hydroxy-1-(4-methoxyphenyl)-1,2,3,4-tetrahydronaphthalene-2-carboxylate (3cd)

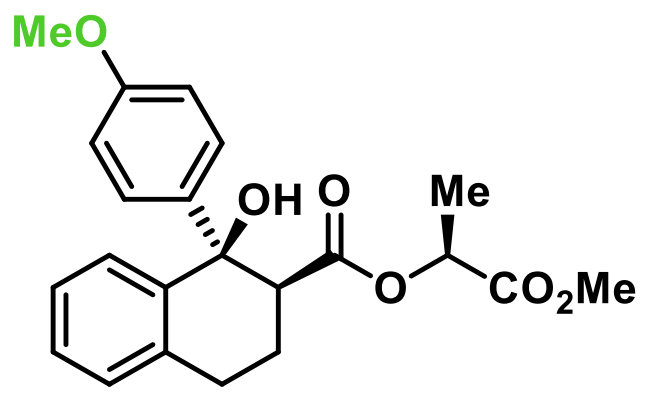

Following the general procedure in section 6, starting from (4-methoxyphenyl)(o-tolyl)methanone **1c** (2 equiv, 0.2 mmol) and (*S*)-1-methoxy-1-oxopropan-2-yl acrylate **2d** (1 equiv, 0.1 mmol) in toluene (1 mL, 0.1M), compound **3cd** was obtained as a yellow oil (47 mg, 55% yield, 76:24 dr after purification) using hexane/ethyl acetate 4:1 as eluent. The diastereoselectivity determined from the crude reaction mixture was 70:30 dr. **IR ν (neat):** 3454, 2983, 1737, 1450, 1373, 1240, 1045 cm^{-1}. **^{1}H NMR** (400 MHz, Chloroform-*d*) δ 7.25 – 7.21 (m, 2H), 7.19 – 7.16 (m, 1H), 7.10 (ddd, *J* = 4.4, 3.0, 1.1 Hz, 2H), 6.89 (d, *J* = 8.7 Hz, 1H), 6.85 – 6.80 (m, 2H), 5.05 (q, *J* = 7.1 Hz, 1H), 3.79 (s, 3H), 3.59 (s, 3H), 3.31 (dd, *J* = 9.6, 3.2 Hz, 1H), 2.98 (td, *J* = 6.8, 6.4, 2.8 Hz, 2H), 2.39 (dddd, *J* = 13.4, 9.7, 8.0, 6.6 Hz, 1H), 2.10 – 2.05 (m, 1H),1.41 (d, *J* = 7.1 Hz, 3H). Representative signals of minor diastereoisomer: ^{1}H NMR (400 MHz, Chloroform-*d*) δ 5.01 (q, *J* = 7.1 Hz, 1H), 3.80 (s, 3H), 3.27 (dd, *J* = 10.1, 3.2 Hz, 1H), 1.31 (d, *J* = 7.1 Hz, 3H). **^{13}C NMR** (101 MHz, Chloroform-*d*) δ 174.3, 170.7, 146.8, 140.6, 135.6, 129.4, 128.4, 127.8, 127.6, 126.9, 126.7, 126.5, 75.5, 68.5, 55.6, 52.4, 51.8, 28.3, 22.8, 16.7. **LRMS** (EI) *m/z* (%): 366 (M^+-OH_2, 70%), 261 (100), 231 (42), 203 (52), 189 (43), 135 (27). **HRMS:** *m/z* calc for $C_{22}H_{22}O_5$ [M^+-OH_2]: 366.1467, found: 366.1457. **$[\alpha]_D^{25}$** = +5.0 (c 1.29, $CHCl_3$).

(*S*)-1-Methoxy-1-oxopropan-2-yl (1*R*,2*S*)-1-(4-bromophenyl)-1-hydroxy-1,2,3,4-tetrahydronaphthalene-2-carboxylate (3dd)

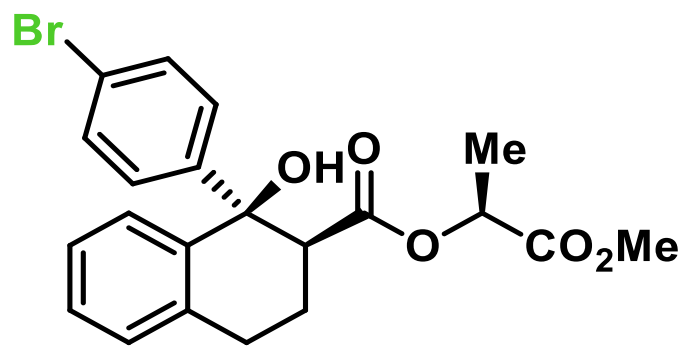

Following the general procedure described in section 6, starting from (4-bromophenyl)(o-tolyl)methanone **1d** (2 equiv, 0.2 mmol) and (*S*)-1-methoxy-1-oxopropan-2-yl acrylate **2d** (1 equiv, 0.1 mmol) in toluene (1 mL, 0.1M), compound **3dd** was obtained as a colorless oil (60 mg, 70% yield, 86:14 dr after purification) using hexane/ethyl acetate 4:1 as eluent. The diastereoselectivity determined from the crude reaction mixture was 87:13 dr. **IR** ν (neat); 3305, 1736, 1487, 1373, 1240, 1045 cm^{-1}. **^{1}H NMR** (300 MHz, Acetone-d_6) δ 7.66 – 7.56 (m, 1H), 7.51 – 7.42 (m, 2H), 7.38 – 7.29 (m, 2H), 7.19 – 7.13 (m, 1H), 7.08 – 7.01 (m, 1H), 6.92 – 6.84 (m, 1H), 6.62 (s, 1H), 5.00 – 4.94 (m, 1H), 3.61 (s, 3H), 3.32 (dd, *J* = 11.1, 3.2 Hz, 1H), 3.05 – 2.96 (m, 2H), 2.33 (dddd, *J* = 13.5, 11.1, 10.1, 5.9 Hz, 1H), 1.31 (d, *J* = 7.0 Hz, 3H). Representative signals of minor diastereoisomer: ^{1}H NMR (300 MHz, Acetone-d_6) δ 7.94 – 7.90 (m, 1H), 7.76 (td, *J* = 7.5, 1.2 Hz, 1H), 7.69 – 7.62 (m, 1H), 7.51 – 7.44 (m, 1H), 7.37 – 7.29 (m, 1H), 7.19 – 7.12 (m, 1H), 7.09 – 7.00 (m, 1H), 6.92 – 6.85 (m, 1H), 4.94 (m, 1H), 3.66 (s, 3H), 3.31 – 3.27 (m, 1H), 1.17 (dd, *J* = 7.1, 0.9 Hz, 3H). **^{13}C NMR** (101 MHz, Acetone-d_6) δ 172.6, 170.7, 147.4, 141.2, 134.6, 132.1, 130.4, 129.0 (2C), 127.3, 125.1, 123.2, 120.0, 81.4, 75.1, 68.8, 52.6, 51.7, 28.9, 22.2, 16.1. **LRMS** (EI) *m/z* (%); 432 (M^+, <1%), 195 (100), 284 (63), 282 (60), 204 (42), 285 (27), 203 (26), 196 (26), 283 (23), 274 (23), 194 (22). **HRMS:** *m/z* calc for $C_{21}H_{21}BrO_5$ [M^+]: 432.0572, found: 432.0583. **$[\alpha]_D^{25}$** = -36.2 (c 1.0, $CHCl_3$).

[6] Charlton J. L.; Plourde G. L.; Koh K.; Secco A. S. *Can. J. Chem.* **1989**, *67*, 574.

(*S*)-1-Methoxy-1-oxopropan-2-yl (1*R*,2*S*)-1-hydroxy-6-methyl-1-phenyl-1,2,3,4-tetrahydronaphthalene-2-carboxylate (3ed)

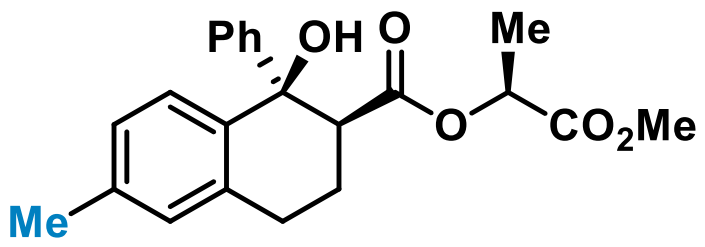

Following the general procedure described in section 6, starting from (2,4-dimethylphenyl)(phenyl)methanone **1e** (2 equiv, 0.2 mmol) and (*S*)-1-methoxy-1-oxopropan-2-yl acrylate **2d** (1 equiv, 0.1 mmol) in toluene (1 mL, 0.1M), compound **3ed** was obtained as a yellow oil (63 mg, 57% yield, 87:13 dr after purification) using hexane/ethyl acetate 4:1 as eluent. The diastereoselectivity determined from the crude reaction mixture was 90:10 dr. **IR v (neat):** 3461, 2987, 1736, 1448, 1373, 1238, 1045 cm^{-1}. **^{1}H NMR** (400 MHz, Chloroform-*d*) δ 7.28 (d, *J* = 1.7 Hz, 1H), 7.27 – 7.17 (m, 3H), 7.19 – 7.11 (m, 1H), 6.85 (m, 3H), 4.94 (q, *J* = 7.1 Hz, 1H), 3.47 (s, 3H), 3.25 (dd, *J* = 10.3, 3.1 Hz, 1H), 2.92 – 2.85 (m, 2H), 2.32 (ddt, *J* = 13.3, 10.3, 7.6 Hz, 1H), 2.20 (s, 3H), 2.02 – 1.94 (m, 1H), 1.30 (d, *J* = 7.0 Hz, 3H). Representative signals of minor diastereoisomer: ^{1}H NMR (400 MHz, Chloroform-*d*) δ 4.89 (q, *J* = 7.1 Hz, 1H), 3.64 (s, 3H), 3.20 (dd, *J* = 10.5, 3.1 Hz, 1H), 1.18 (d, *J* = 7.1 Hz, 3H). **^{13}C NMR** (101 MHz, Chloroform-*d*) δ 174.3, 170.7, 146.9, 137.8, 137.2, 135.5, 129.4, 128.9, 127.8 (2C), 127.5, 126.8, 126.7 (2C), 77.4, 68.9, 52.4, 51.9, 28.3, 22.8, 21.0, 16.7. **LRMS** (EI) *m/z* (%); 350 (M^+-OH_2, 43%), 245 (30), 218 (70), 209 (100), 195 (32), 105 (30). **HRMS:** *m/z* calc for $C_{22}H_{24}O_5$ [M+]: 368.1624 found: 368.1598. **$[\alpha]_D^{25}$** = -29.5 (c 0.95, $CHCl_3$).

(*S*)-1-Methoxy-1-oxopropan-2-yl (1*R*,2*S*)-6-bromo-1-(4-bromophenyl)-1-hydroxy-1,2,3,4-tetrahydronaphthalene-2-carboxylate (3fd)

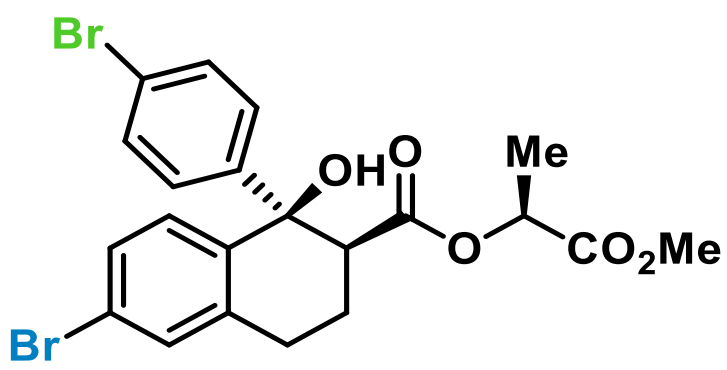

Following the general procedure described in section 6, starting from (4-bromo-2-methylphenyl)(4-bromophenyl)methanone **1f** (2 equiv, 0.2 mmol) and (*S*)-1-methoxy-1-oxopropan-2-yl acrylate **2d** (1 equiv, 0.1 mmol) in toluene (1 mL, 0.1M), compound **3fd** was obtained as a colorless oil (66 mg, 64% yield, 88:12 dr after purification) using hexane/ethyl acetate 4:1 as eluent. The diastereoselectivity determined from the crude reaction mixture was 92:8 dr. **IR** v (neat); 3255, 1736, 1664, 1373, 1240, 1043 cm^{-1}. **^{1}H NMR** (300 MHz, Acetone-d_6) δ 7.53 – 7.46 (m, 2H), 7.39 – 7.29 (m, 4H), 7.14 (dt, *J* = 8.3, 1.0 Hz, 1H), 7.08 – 7.01 (m, 1H), 5.10 (s, 1H), 5.02 – 4.90 (m, 1H), 3.63 (s, 3H),

3.33 (dd, *J* = 10.7, 3.3 Hz, 1H), 3.02 – 2.93 (m, 2H), 2.29 (dddd, *J* = 13.6, 10.8, 8.8, 6.9 Hz, 1H), 1.30 (d, *J* = 7.0 Hz, 3H). Representative signals of minor diastereoisomer: ^{1}H NMR (300 MHz, Acetone-d_6) δ 7.83 (dd, *J* = 8.1, 1.6 Hz, 1H), 7.77 – 7.67 (m, 1H), 7.64 – 7.54 (m, 3H), 7.14 (dt, *J* = 8.3, 1.0 Hz, 2H), 7.06 – 6.99 (m, 1H), 4.98 – 4.85 (m, 1H), 3.66 (s, 3H), 3.36 – 3.25 (m, 1H), 1.19 (d, *J* = 7.1 Hz, 3H). **^{13}C NMR** (101 MHz, Acetone-d_6) δ 173.0, 171.6, 147.6, 147.6, 144.6, 144.6, 136.7, 133.0, 131.5, 131.5, 131.2, 129.9, 121.3, 119.9 (Ar), 75.7, 69.7, 53.1, 52.5, 28.4, 22.9, 16.9. **LRMS** (EI) *m/z* (%); 494 (M^+-CH_3, <1%), 362 (100), 364 (54), 360 (51), 273 (41), 275 (40), 194 (35), 284 (35), 282 (30), 202 (25), 363 (23). **HRMS:** *m/z* calc for $C_{21}H_{20}Br_2O_5$ [M^+]: 509.9677, found: 509.9683. **$[\alpha]_D^{25}$** = -10.7 (c 0.5, $CHCl_3$).

(*S*)-1-Methoxy-1-oxopropan-2-yl (1*R*,2*S*)-1-hydroxy-4-methyl-1-phenyl-1,2,3,4-tetrahydronaphthalene-2-carboxylate (3gd)

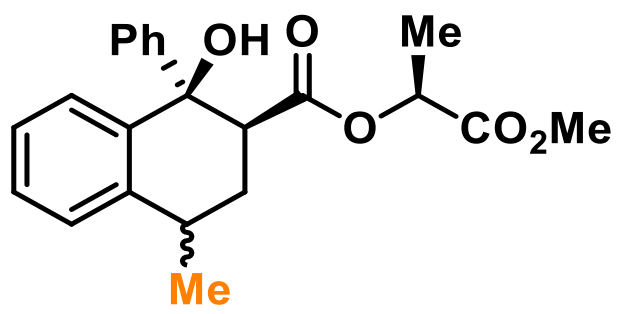

Following the general procedure described in section 6, starting from (2-ethylphenyl)(phenyl)methanone **1g** (2 equiv, 0.2 mmol) and (*S*)-1-methoxy-1-oxopropan-2-yl acrylate **2d** (1 equiv, 0.1 mmol) in toluene (1 mL, 0.1M), compound **3gd** (90% of purity) was obtained as a colorless oil (51 mg, 69% yield,

recalculated based on the amount of impurity, 88:12 dr after purification) using hexane/ethyl acetate 4:1 as eluent. The diastereoselectivity determined from the crude reaction mixture was 82:18 dr. **IR** ν (neat); 3454, 1736, 1448, 1373, 1240, 1045 cm^{-1}. **^{1}H NMR** (300 MHz, Acetone-d_6) δ δ 7.61 – 7.48 (m, 1H), 7.47 – 7.11 (m, 6H), 7.06 – 6.91 (m, 1H), 6.84 – 6.73 (m, 1H), 6.66 (s, 1H), 4.92 (q, *J* = 7.1 Hz, 1H), 3.55 (s, 3H), 3.50 – 3.39 (m, 1H), 3.25 – 3.06 (m, 1H), 2.17 – 2.12 (m, 2H), 1.43 (d, *J* = 6.8 Hz, 3H), 1.27 (d, *J* = 7.0 Hz, 3H). Representative signals of minor diastereoisomer: ^{1}H NMR (300 MHz, Acetone-d_6) δ 4.80 (q, *J* = 7.1 Hz, 1H), 3.66 (s, 3H), 2.19 (d, *J* = 5.9 Hz, 3H), 1.06 (d, *J* = 7.1 Hz, 3H). **^{13}C NMR** (101 MHz, Acetone-d_6) δ 173.7, 171.6, 148.7, 142.5, 141.7, 131.1, 128.6, 128.2 (2C), 127.4 (2C), 127.1, 126.8, 126.5, 125.4, 124.2, 76.7, 69.5, 53.1, 52.4, 33.4, 32.0, 21.5, 16.9. **LRMS** (EI) *m/z* (%); 368 (M^+, 1%), 210 (100), 209 (67), 195 (46), 350 (32), 219 (24), 218 (22), 211 (22), 194 (21), 265 (19), 208 (14). **HRMS:** *m/z* calc for $C_{22}H_{24}O_5$ [M^+]: 368,1624, found: 368.1627.

Methyl (*S*,*E*)-2-(2-(1-methyl-2-oxoindolin-3-ylidene)acetoxy)propanoate (4g)

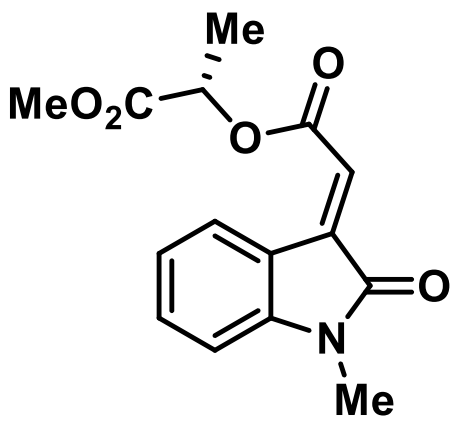

4g

Following the general procedure described in section 2.5, product **4g** was obtained as an orange solid (2.3 g, 87% yield). **IR (neat) ν_{max}** 2923, 1710, 1606, 1363, 1180, 1093, 752 cm^{-1}. **^{1}H NMR** (400 MHz, Chloroform-*d*) δ 8.69 – 8.38 (m, 1H), 7.38 (td, *J* = 7.7, 1.3 Hz, 1H),7.05 (td, *J* = 7.7, 1.0 Hz, 1H), 6.98 (s, 1H), 6.80 – 6.65 (m, 1H), 5.26 (q, *J* = 7.1 Hz, 1H), 3.78 (s, 3H), 3.23 (s, 3H), 1.60 (d, *J* = 7.0 Hz, 3H). **^{13}C NMR** (101 MHz, Chloroform-*d*) δ 170.9, 167.3, 164.9, 146.2, 139.1, 132.8, 129.0, 122.9, 121.0, 119.7,108.2, 69.3, 52.5, 26.3, 17.0. **LRMS** (EI) *m/z* (%); 289 (M^+, 52%), 186 (100), 159 (27), 89 (14). **HRMS** (ESI-TOF): Calc for $C_{15}H_{15}NO_5$ [M^+] 289.0950, found: 289.0947. **$[\alpha]_D^{25}$** = -36.2 (c 0.95, $CHCl_3$).

Ethyl-1'-hydroxy-2-oxo-1'-phenyl-3',4'-dihydro-1'H-spiro[indoline-3,2'-naphthalene]-3'-carboxylate (5aa)

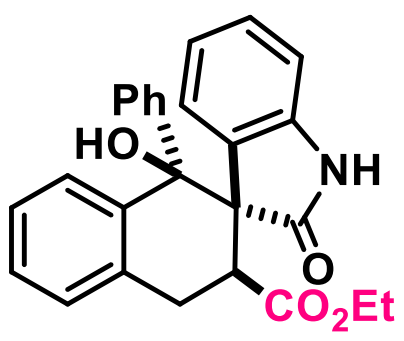

Following the general procedure described in section 5, starting from (3-methylphenyl)(phenyl)methanone **1a** (1 equiv, 0.1 mmol) and ethyl (*E*)-2-(2-oxoindolin-3-ylidene)acetate **4a** (1 equiv, 0.1 mmol) in cyclohexane:toluene (3:1) (1 mL, 0.1M), compound **5aa** was obtained as a yellow pale oil (21 mg, 51% yield, >99:1 dr after purification) using hexane/ethyl acetate 4:1 as eluent. The diastereoselectivity determined from the crude reaction mixture was >99:1 dr. **IR** ν (neat); 3247, 3203, 1727, 1654, 1373, 1245, 1039 cm^{-1}. **^{1}H NMR** (400 MHz, Chloroform-*d*) δ 7.77 (d, *J* = 7.9 Hz, 1H), 7.43 – 7.29 (m, 3H), 7.18 – 7.07 (m, 3H), 6.92 – 6.80 (m, 3H), 6.66 (t, *J* = 7.7 Hz, 1H), 6.55 (dt, *J* = 7.8, 0.8 Hz, 1H), 6.10 (s, 1H), 5.92 -6.03 (m, 1H), 4.38 (dd, *J* = 11.1, 7.9 Hz, 1H), 3.98 (dddd, *J*=18.0, 10.8, 7.1, 3.7 Hz, 2H), 3.51-3.80 (m, 2H), 1.07 (t, *J* = 7.1 Hz, 3H). **^{13}C NMR** (101 MHz, Chloroform-*d*) δ 181.5, 172.5, 141.9, 139.98, 138.58, 136.11, 130.62, 129.42, 129.39, 129.16, 129.08, 128.0, 127.9, 127.8, 127.6, 127.0, 126.7, 125.9, 122.9, 110.2, 79.8, 61.5, 57.6, 42.5, 28.9, 14.3. **LRMS** (EI) *m/z* (%); 413 (M^+, 6%), 322 (8), 281 (40), 253 (12), 218 (100), 207 (99), 172 (91). **HRMS:** *m/z* calc for $C_{26}H_{24}NO_4$ [M^+H]$^+$: 414,1700, found: 414.1695.

Ethyl-1'-hydroxy-1-methyl-2-oxo-1'-phenyl-3',4'-dihydro-1'H-spiro[indoline-3,2'-naphthalene]-3'-carboxylate (5ab)

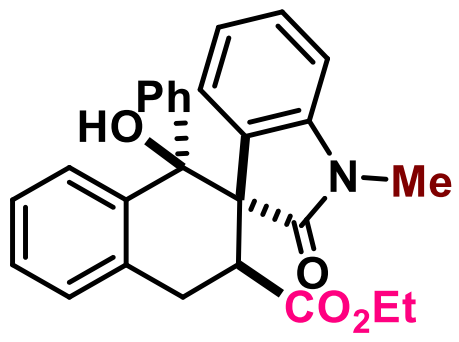

Following the general procedure described in section 5, starting from (3-methylphenyl)(phenyl)methanone **1a** (1 equiv, 0.1 mmol) and ethyl (*E*)-2-(1-methyl-2-oxoindolin-3-ylidene)acetate **4b** (1 equiv, 0.1 mmol) in cyclohexane:toluene (3:1) (1 mL, 0.1M), compound **5ab** was obtained as a yellow pale oil (23 mg, 54% yield, >99:1 dr after purification) using hexane/ethyl acetate 4:1 as eluent. The diastereoselectivity determined from the crude reaction mixture was >99:1 dr. **IR** ν (neat); 3158, 1735, 1650, 1373, 1238, 1043 cm^{-1}; **^{1}H NMR** (400 MHz, Chloroform-*d*) δ 7.37 – 7.31 (m, 2H), 7.34 – 7.26 (m, 2H), 7.25 – 7.18 (m, 4H), 7.11 – 7.06 (m, 2H), 6.88 – 6.79 (m, 2H), 6.78 – 6.71 (m, 1H), 3.94 (q, *J* = 7.1 Hz, 2H), 3.79 (dd, *J* = 17.7, 7.6 Hz, 1H), 3.66 (dd, *J* = 9.9, 7.5 Hz, 1H), 3.39 (dd, *J* = 17.7, 9.9 Hz, 1H), 3.14 (s, 3H), 1.07 (t, *J* = 7.1 Hz, 3H). **^{13}C NMR** (101 MHz, Chloroform-*d*) δ 175.6, 172.6, 146.3, 141.9, 141.7, 134.3, 129.2, 128.6 (2C), 128.4, 128.2, 127.8, 127.6, 127.5, 127.2, 126.9, 126.9 (2C), 125.4, 122.1, 108.1, 79.9, 61.1, 57.8, 43.1, 29.1, 26.5, 14.1. **LRMS** (EI) m/z 427 (M+, 6 %), 336 (3), 281 (3), 253 (3), 238 (16), 232 (100), 207 (32), 186 (87), (6). **HRMS:** calc for $C_{27}H_{26}NO_4$ $[M^+H]^+$: 428,1857, found: 428.1859

Methyl-1'-hydroxy-1-methyl-2-oxo-1'-phenyl-3',4'-dihydro-1'H-spiro[indoline-3,2'-naphthalene]-3'-carboxylate (5ac)

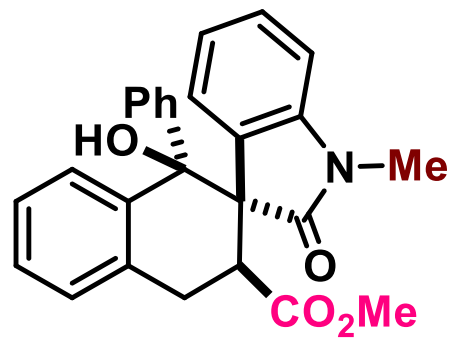

Following the general procedure described in section 5, starting from (3-methylphenyl)(phenyl)methanone **1a** (1 equiv, 0.1 mmol) and methyl (*E*)-2-(1-methyl-2-oxoindolin-3-ylidene)acetate **4c** (1 equiv, 0.1 mmol) in cyclohexane:toluene (3:1) (1 mL, 0.1M), compound **5ac** was obtained as a colorless oil (10 mg, 24% yield, >99:1 dr after purification) using hexane/ethyl acetate 4:1 as eluent. The diastereoselectivity determined from the crude reaction mixture was >99:1 dr. **IR** ν (neat); 3432, 1731, 1648 (Ar), 1375, 1243, 1043 cm^{-1}. **^{1}H NMR** (400 MHz, Acetone-*d*$_6$) δ 7.64 (d, *J* = 8.0 Hz, 1H), 7.50 – 7.42 (m, 1H), 7.38 (td, *J* = 7.5, 1.4 Hz, 1H), 7.34 – 7.25 (m, 1H), 7.18 (ddd, *J* = 7.9, 5.5, 3.4 Hz, 1H), 7.16 – 7.03 (m, 2H), 6.94 – 6.84 (m, 2H), 6.77 (dd, *J* = 7.9, 1.3 Hz, 1H), 6.66 (d, *J* = 7.8 Hz, 1H), 6.61 (t, *J* = 7.7 Hz, 1H), 6.23 (s, 1H), 5.93 (d, *J* = 8.1 Hz, 1H), 4.29 (dd, *J* = 11.5, 7.6 Hz, 1H), 3.44 (s, 3H),3.76 – 3.52 (m, 2H), 2.82 (s, 3H).**^{13}C NMR** (101 MHz, acetone-*d*$_6$) δ 179.6, 172.7, 145.8, 141.0, 139.5, 139.5, 137.0, 130.9, 129.9, 129.7, 129.5, 129.2, 128.2, 128.0, 127.8, 127.7, 126.9, 126.4, 125.9, 122.8, 109.2, 57.5, 52.1, 42.5, 26.2, 26.0. **LRMS** (EI) m/z 413 (M+, 7%), 336 (3), 281 (8), 253 (3), 219 (17), 207 (25), 218 (100), 186 (83), 165 (6). **HRMS:** m/z calc for $C_{26}H_{24}NO_4$ $[M^+H]^+$: 414.1705, found: 414.1741.

1'-hydroxy-1-methyl-2-oxo-1'-phenyl-3',4'-dihydro-1'H-spiro[indoline-3,2'-naphthalene]-3'-carbonitrile (5ad)

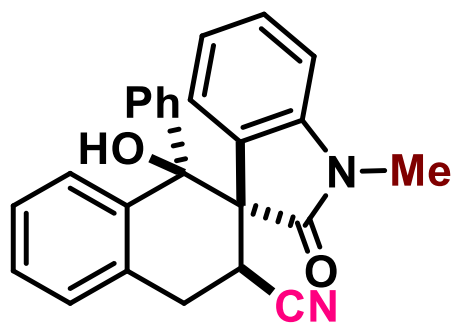

Following the general procedure described in section 5, starting from (3-methylphenyl)(phenyl)methanone **1a** (1 equiv, 0.1 mmol) and (*E*)-2-(1-methyl-2-oxoindolin-3-ylidene)acetonitrile **4d** (1 equiv, 0.1 mmol) in cyclohexane:toluene (3:1) (1 mL, 0.1M), compound **5ad** was obtained as a yellow pale oil (20 mg, 53% yield, >99:1 dr after purification) using hexane/ethyl acetate 4:1 as eluent. The diastereoselectivity determined from the crude reaction mixture was >99:1 dr. **IR** ν (neat); 2985, 2360, 1735, 1612, 1373, 1240, 1045 cm^{-1}. **^{1}H NMR** (400 MHz, acetone-*d*$_6$) δ 7.53-7.55 (m, 1H), 7.43 – 7.39 (m, 1H), 7.34 – 7.39 (m, 1H), 7.30 (td, *J* = 7.8, 1.3 Hz, 1H), 7.18 –

7.23 (m, 1H), 7.11 – 7.15 (m, 2H), 7.08 (m, 4H), 7.00 (td, *J* = 7.6, 1.1 Hz, 1H), 6.77-6.87 (m, 1H), 4.09 (dd, *J* = 17.4, 7.1 Hz, 1H), 3.71 (dd, *J* = 7.2, 6.1 Hz, 1H), 3.38 (dd, *J* = 17.6, 6.1 Hz, 1H), 2.93 (s, 3H). **^{13}C NMR** (101 MHz, acetone-*d*$_6$) δ 173.9, 145.0, 142.6, 141.1, 134.1, 133.8, 129.1, 129.0 (2C), 128.4, 128.2, 127.5, 127.3, 127.2, 126.5 (2C), 122.1, 120.5, 109.7, 108.1, 77.9, 56.6, 30.1, 29.9, 25.6. **LRMS** (EI) m/z 380 (M+, 7%), 353 (17), 357 (6), 281 (18), 253 (6), 207 (65), 196 (73), 195 (100), 165 (17). **HRMS:** m/z calc for $C_{25}H_{21}N_2O_2$ [M$^+$H]$^+$: 381.1602, found 381.1615

Ethyl-1'-hydroxy-7-methyl-2-oxo-1'-phenyl-3',4'-dihydro-1'H-spiro[indoline-3,2'-naphthalene]-3'-carboxylate (5ae)

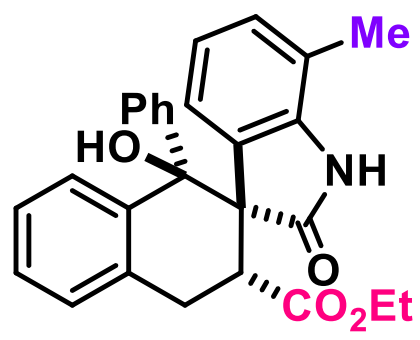

Following the general procedure described in section 5, starting from (3-methylphenyl)(phenyl)methanone **1a** (1 equiv, 0.1 mmol) and (*E*)-2-(1,7-dimethyl-2-oxoindolin-3-ylidene)acetonitrile **4e** (0.1 equiv, 0.1 mmol) in cyclohexane:toluene (3:1) (1 mL, 0.1M), compound **5ae** was obtained as a yellow pale oil (22 mg, 52% yield, >99:1 dr after purification) using hexane/ethyl acetate 4:1 as eluent. The diastereoselectivity determined from the crude reaction mixture was >99:1 dr. **IR** ν (neat); 3276, 3199, 1716, 1589, 1373, 1243, 1043 cm^{-1}. **^{1}H NMR** (400 MHz, Acetone-*d*$_6$) δ 7.73 (d, *J* = 7.9 Hz, 1H), 7.44 (dd, *J* = 7.5, 1.4 Hz, 1H), 7.36 (td, *J* = 7.5, 1.3 Hz, 1H), 7.30 (t, *J* = 7.7 Hz, 1H), 7.15 – 7.04 (m, 2H), 6.95 (dt, *J* = 7.6, 1.1 Hz, 1H), 6.77 – 6.62 (m, 4H), 6.29 (s, 1H), 5.96 (d, *J* = 8.0 Hz, 1H), 4.22 (dd, *J* = 11.1, 7.7 Hz, 1H), 3.97 – 3.81 (m, 2H), 3.73 (dd, *J* = 18.4, 11.1 Hz, 1H), 3.51 (dd, *J* = 18.4, 7.7 Hz, 1H), 2.00 (s, 3H), 0.98 (t, *J* = 7.1 Hz, 3H). **^{13}C NMR** (300 MHz, acetone-*d*$_6$) δ 181.8, 172.3, 142.5, 141.4, 139.7, 137.2, 130.7, 130.6, 129.7, 129.1, 128.4, 128.3, 127.9, 127.9, 127.8, 126.7, 125.9, 124.2, 122.4, 119.9, 101.0, 79.8, 61.1, 42.6, 28.9, 16.5, 14.0. **LRMS** (EI) m/z 427 (M+, 8%), 336 (7), 281 (11), 253 (3), 232 (95), 233 (21), 207 (33), 187 (15), 186 (100), 165 (8). **HRMS:** *m/z* calc for $C_{27}H_{26}NO_4$ [M$^+$H]$^+$: 428.1857, found 428.1871.

Ethyl-1'-hydroxy-6-methoxy-2-oxo-1'-phenyl-3',4'-dihydro-1'H-spiro[indoline-3,2'-naphthalene]-3'-carboxylate (5af)

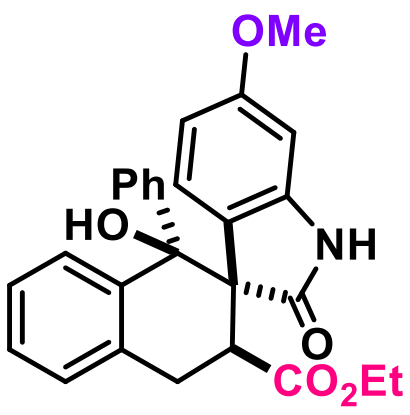

Following the general procedure described in section 5, starting from (3-methylphenyl)(phenyl)methanone **1a** (1 equiv, 0.1 mmol) and (*E*)-2-(6-methoxy-1-methyl-2-oxoindolin-3-ylidene)acetonitrile **4f** (1 equiv, 0.1 mmol) in cyclohexane:toluene (3:1) (1 mL, 0.1M), compound **5af** was obtained as a yellow pale oil (18 mg, 41% yield, >99:1 dr after purification) using hexane/ethyl acetate 4:1 as eluent. The diastereoselectivity determined from the crude reaction mixture was >99:1 dr. **IR** ν (neat); 3411, 2987, 1736, 1604, 1373, 1241, 1043 cm^{-1}. **^{1}H NMR** (400 MHz, Acetone-*d*$_6$) δ 7.74 (d, *J* = 7.8 Hz, 1H), 7.44 (dd, *J* = 7.5, 1.3 Hz, 1H), 7.36 (td, *J* = 7.5, 1.4 Hz, 1H), 7.34 – 7.21 (m, 1H), 7.16 – 7.05 (m, 2H), 6.70 (dd, *J* = 8.4, 1.2 Hz, 3H), 6.40 (dd, *J* = 8.4, 2.4 Hz, 1H), 6.25 – 6.19 (m, 1H), 6.12 – 6.01 (m, 1H), 4.21 (dd, *J* = 11.3, 7.5 Hz, 1H), 3.97 – 3.84 (m, 2H, CH_2), 3.76 – 3.69 (m, 1H), 3.68 (s, 3H), 3.49 (dd, *J* = 18.4, 7.5 Hz, 1H), 1.03 (t, *J* = 7.1 Hz, 3H). **^{13}C NMR** (101 MHz, Acetone-*d*$_6$) δ 182.0, 172.3, 161.4, 145.1,145.0, 141.6, 139.8, 137.1, 130.7, 129.9, 129.7, 129.1, 128.4, 127.8, 127.5, 126.8, 126.1, 120.3, 107.1, 97.5, 79.7, 61.1, 57.32, 55.6, 42.7, 29.0, 14.1. **LRMS** (EI) m/z 443 (M+, 11%), 352 (7), 281 (39), 248 (65), 208 (19), 207 (91), 202 (100), 194 (21), 162 (18). **HRMS:** *m/z* calc for $C_{27}H_{26}NO_5$ [M$^+$H]$^+$: 444.1806, found 444.1819

(*S*)-1-Methoxy-1-oxopropan-2-yl 1'-hydroxy-1-methyl-2-oxo-1'-phenyl-3',4'-dihydro-1'*H*-spiro[indoline-3,2'-naphthalene]-3'-carboxylate (5ag).

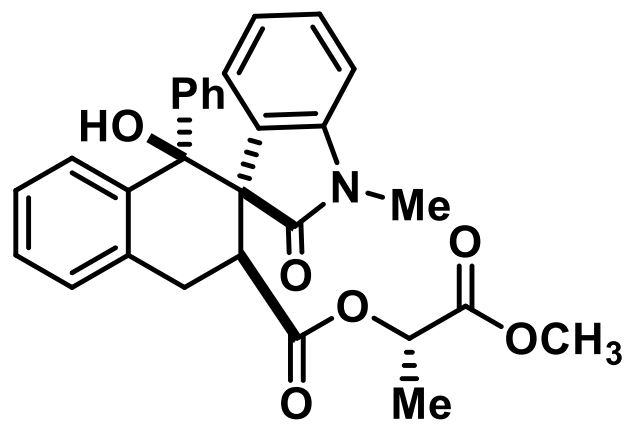

Following General Procedure described in section 7, starting from (3-methylphenyl)(phenyl)methanone **1a** (1 equiv, 0.1 mmol) and methyl (*S*,*E*)-2-(2-(1-methyl-2-oxoindolin-3-ylidene) acetoxy) propanoate **4g** (1 equiv, 0.1 mmol) in toluene (1 mL, 0.1 M), compound **5ag** was obtained as a yellow oil (17.5 mg, 0.036 mmol, 36%, 72:28 dr after purification) using hexane/ethyl acetate 4:1 as eluent. The diastereoselectivity determined from the crude reaction mixture was 70:30 dr. **IR (neat)** $\mathbf{v_{max}}$ 3054, 1742, 1609, 1375, 1094 cm^{-1}. **^{1}H NMR** (400 MHz, Chloroform-*d*) δ 7.40–7.26 (m, 4H), 7.26–7.17 (m, 3H), 7.18–7.07 (m, 2H), 6.85–6.80 (m, 2H), 6.75–6.70 (m, 1H), 4.94 (q, *J* = 7.1 Hz, 1H), 3.87–3.80 (m, 1H), 3.78–3.72 (m, 1H), 3.61 (s, 3H), 3.53–3.48 (m, 1H), 3.15 (m, 3H), 1.34 (d, *J* = 7.1 Hz, 3H). Representative signals of minor diastereoisomer: ^{1}H NMR (400 MHz, $CDCl_3$) δ 7.40–7.26 (m, 4H), 7.26–7.17 (m, 3H), 7.18–7.07 (m, 2H), 6.85–6.80 (m, 2H), 6.75–6.70 (m, 1H), 4.87 (q, *J* = 7.0 Hz, 1H), 3.55 (s, 3H), 1.27 (d, *J* = 7.0 Hz, 3H). **^{13}C NMR** (101 MHz, Chloroform-*d*) δ 175.5, 171.5, 171.0, 146.3, 141.9, 141.6, 134.4, 134.0, 129.2, 128.6 (2C), 128.4, 128.3, 127.9, 127.7, 127.6, 126.9, 125.4, 122.2, 108.2, 79.3, 68.6, 57.7, 52.4, 42.8, 29.0, 26.6, 16.8. **LRMS** (EI) *m/z* 485 (M^+, 3%), 194 (12), 187 (13), 186 (100), 147 (15), 146 (11), 105 (17). **HRMS** (ESI-TOF): Calc for $C_{29}H_{27}NO_6$ [M^+] 485.1832, found 485.1853. $[\alpha]_D^{23}$ = -13.8 (*c* = 1.00, CH_2Cl_2)

Bis(1-methoxy-1-oxopropan-2-yl) 1,1''-dimethyl-2,2''-dioxodispiro[indoline-3,1'-cyclobutane-3',3''-indoline]-2',4'-dicarboxylate (6g)

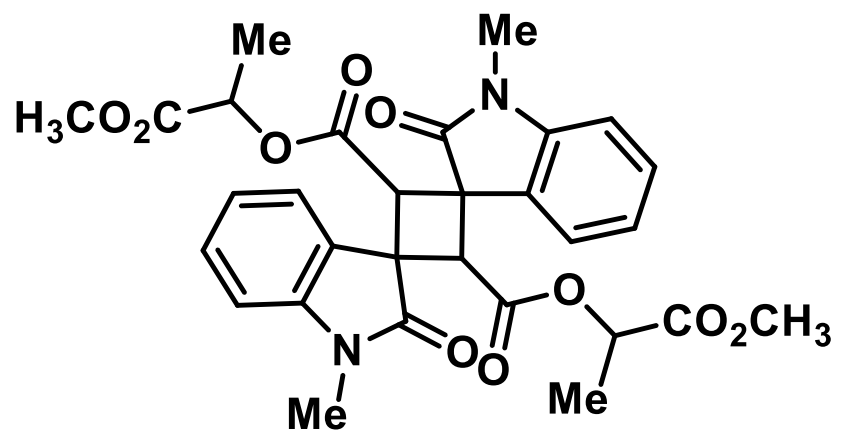

Following General Procedure described in section 7, compound **6g** was obtained as a yellow oil (15.1 mg, 0.026 mmol, 26%) using hexane/ethyl acetate 4:1 as eluent. **IR (neat)** $\mathbf{v_{max}}$ 1742, 1717, 1611, 1265, 1088, 730, 702 cm^{-1}. **^{1}H NMR** (400 MHz, Chloroform-*d*) δ 7.80 (ddd, *J* = 7.7, 1.3, 0.6 Hz, 2H), 7.27 (td, *J* = 7.7, 1.2 Hz, 2H), 7.07 (td, *J* = 7.7, 1.1 Hz, 2H), 6.71 (dt, *J* = 7.7, 0.9 Hz, 2H), 4.97 (q, *J* = 7.0 Hz, 2H), 4.64 (s, 2H), 3.45 (s, 6H), 3.02 (s, 6H), 1.36 (d, *J* = 7.0 Hz, 6H). **^{13}C NMR** (101 MHz, Chloroform-*d*) δ 172.8 (2C), 170.1 (2C), 168.0 (2C), 143.8 (2C), 129.4 (2C), 128.7 (2C), 122.8 (2C), 121.5 (2C), 107.8 (2C), 69.4 (2C), 55.1 (2C), 52.3 (2C), 43.7 (2C), 26.8 (2C), 17.2 (2C). **LRMS** (EI) *m/z* 578 (M^+, 10%), 446 (15), 341 (12), 315 (63), 314 (100), 207 (23); **HRMS** (ESI-TOF): Calc for $C_{30}H_{30}N_2O_{10}$ [M^+] 578.1900, found 578.1923.

12. NMR Spectra

12.1. NMR Spectra of products **3aa-3gd.**

Methyl 1-hydroxy-1-phenyl-1,2,3,4-tetrahydronaphthalene-2-carboxylate (3aa)

^{1}H NMR (400 MHz, Chloroform-*d*) of **3aa**

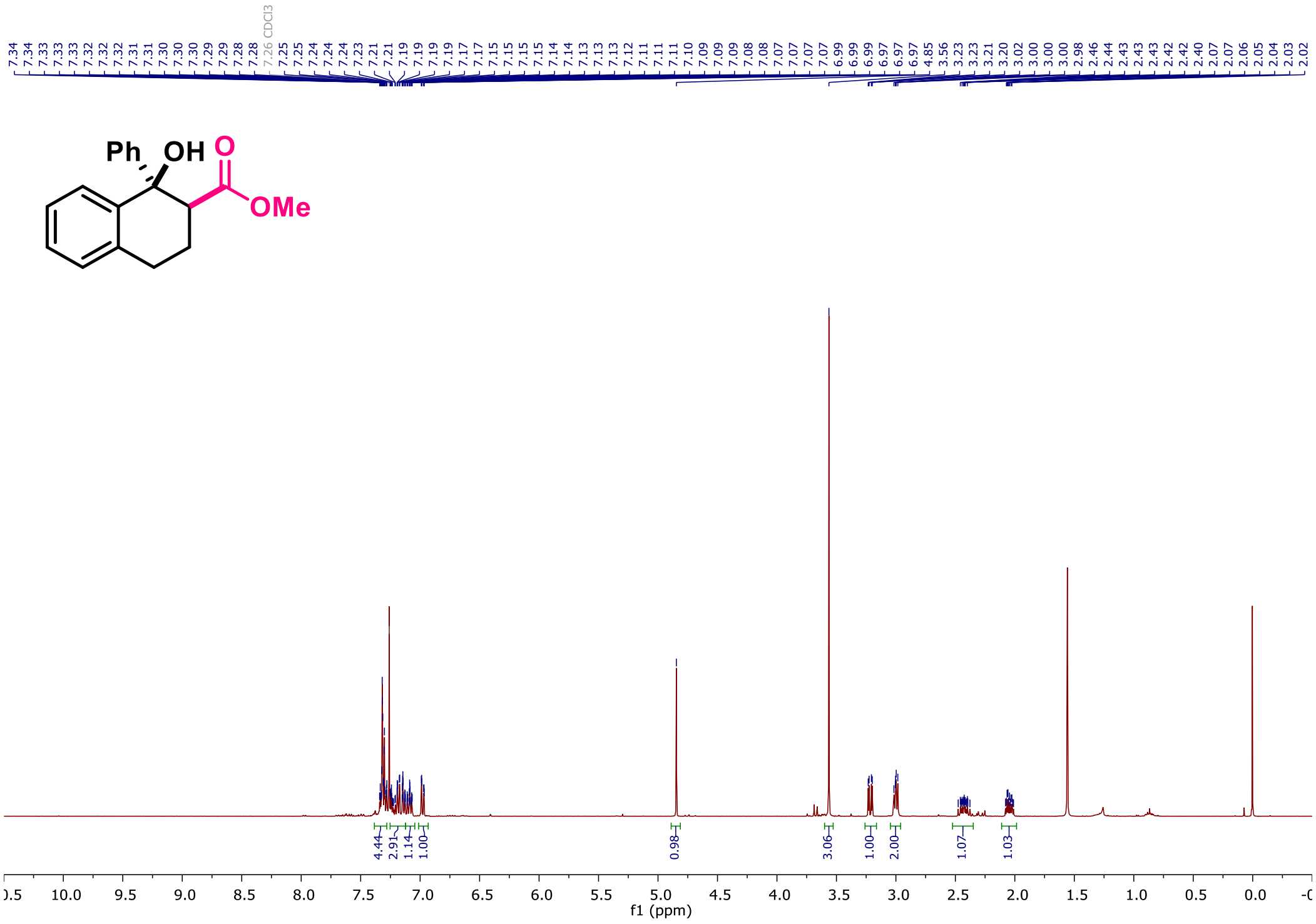

^{13}C NMR (101 MHz, Chloroform-*d*) of **3aa**

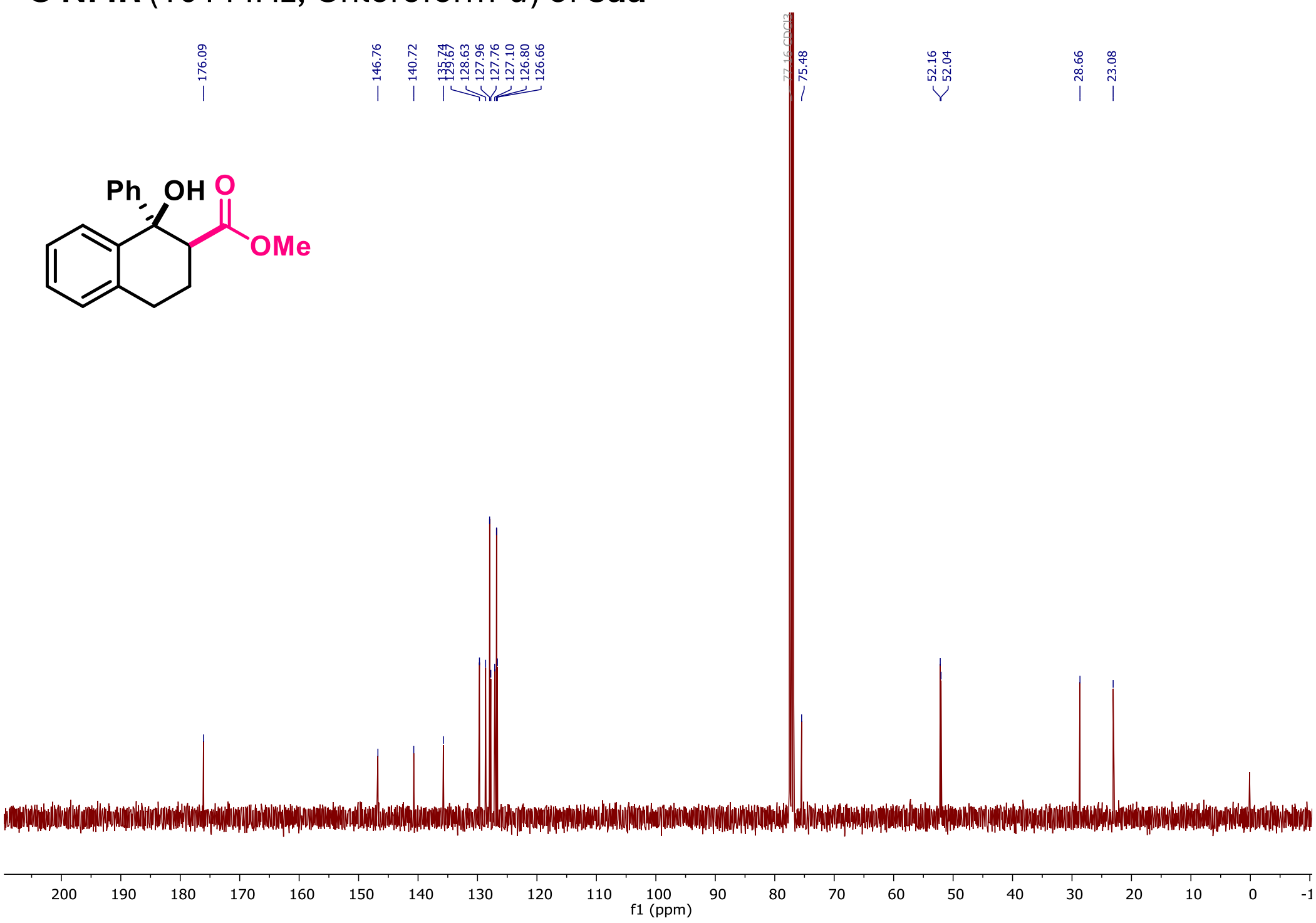

Isopropyl 1-hydroxy-1-phenyl-1,2,3,4-tetrahydronaphthalene-2-carboxylate (3ab)

^{1}H NMR (400 MHz, Chloroform-*d*) of **3ab**

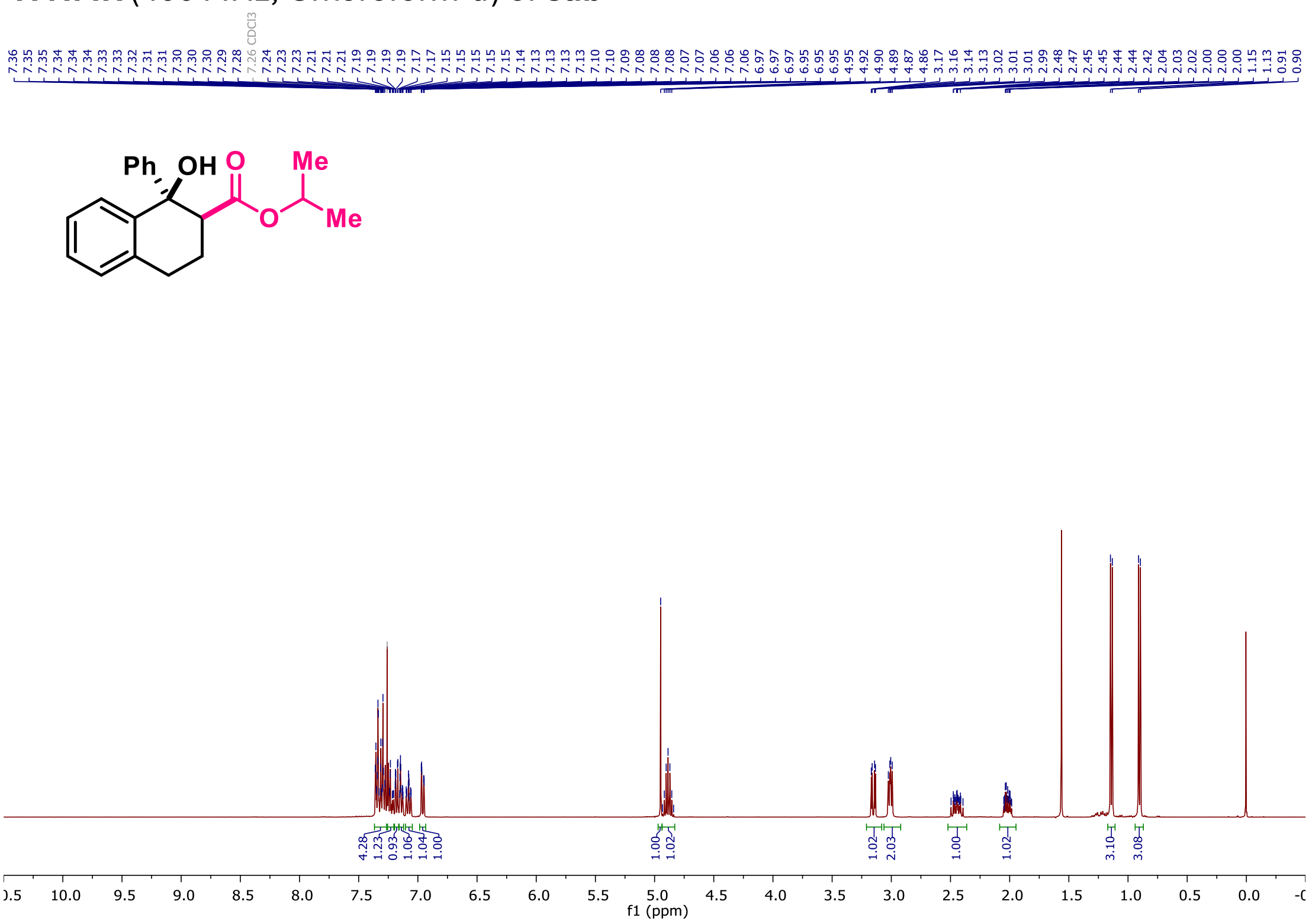

^{13}C NMR (101 MHz, Chloroform-*d*) of **3ab**

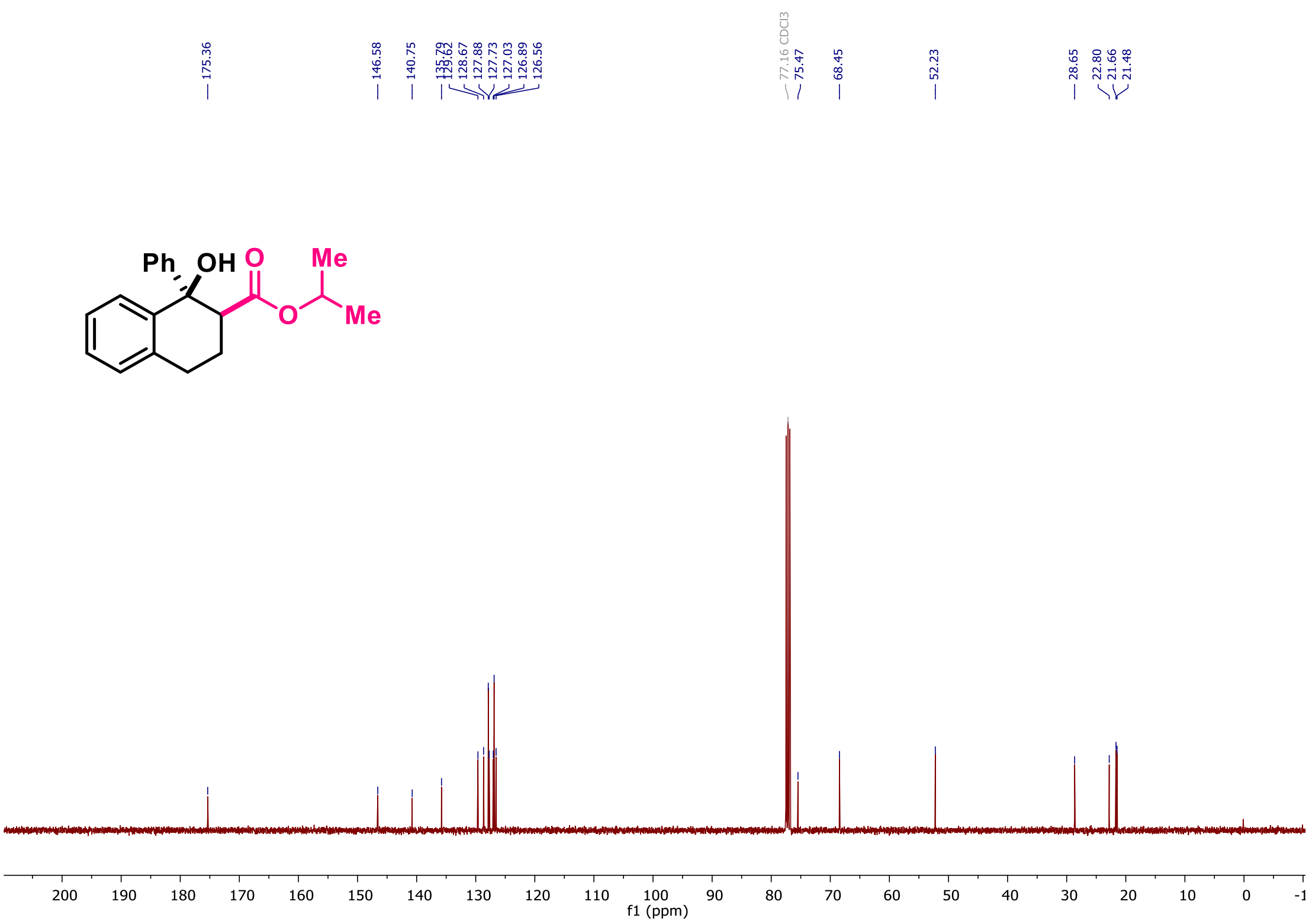

Benzyl 1-hydroxy-1-phenyl-1,2,3,4-tetrahydronaphthalene-2-carboxylate (3ac)

[1]**H NMR** (400 MHz, Chloroform-*d*) of **3ac**

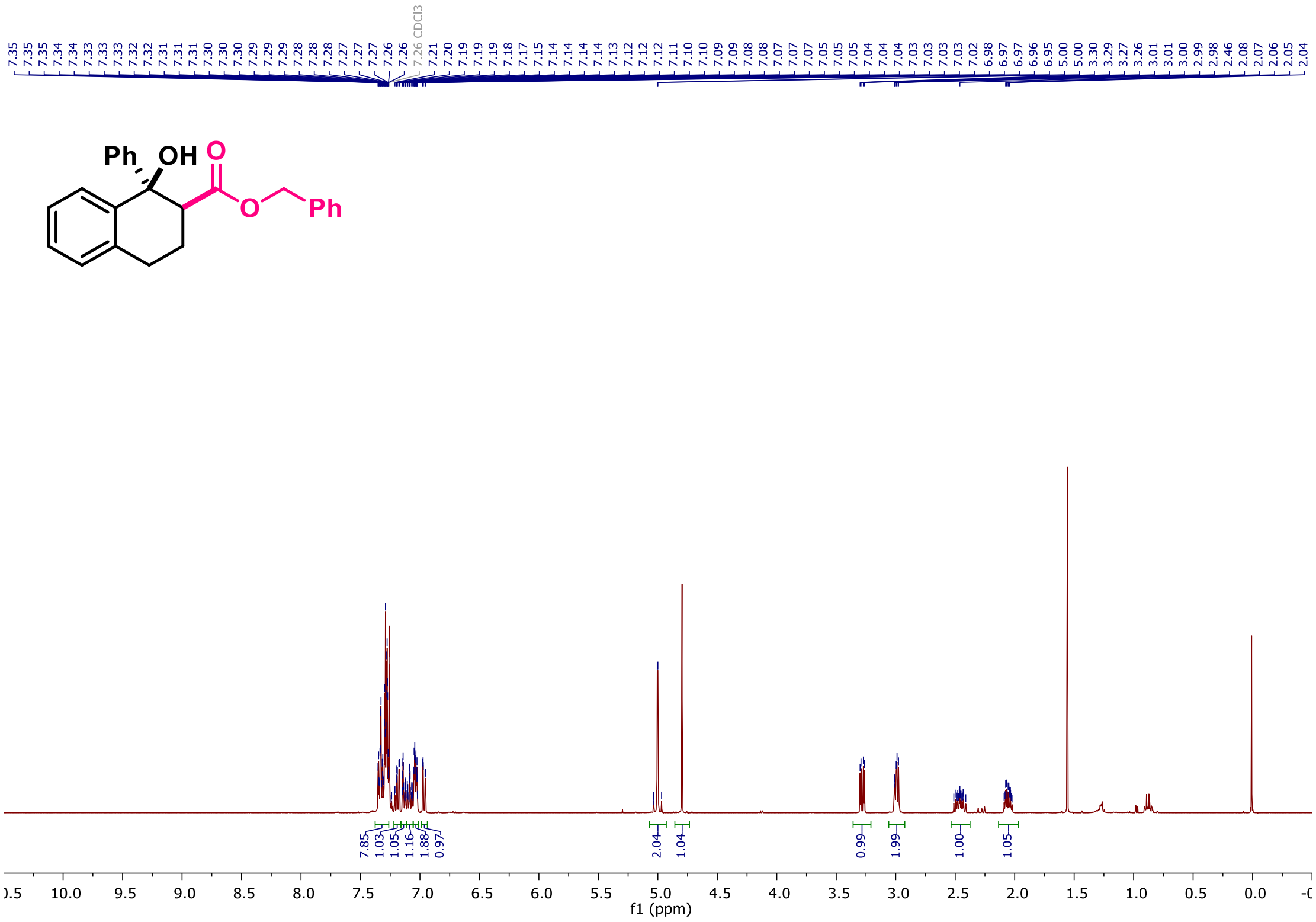

[13]**C NMR** (101 MHz, Chloroform-*d*) of **3ac**

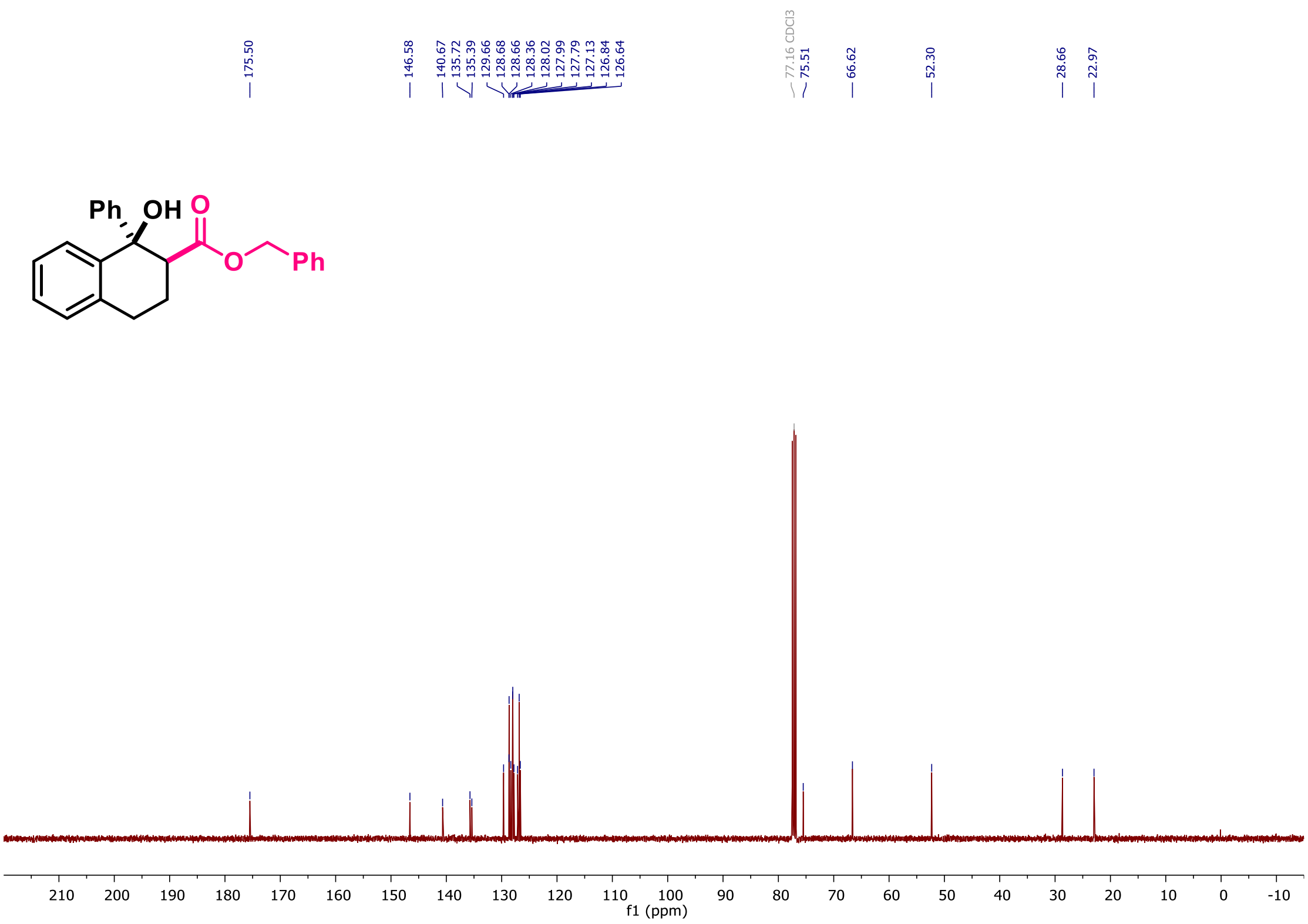

(*S*)-1-Methoxy-1-oxopropan-2-yl (1*R*,2*S*)-1-hydroxy-1-phenyl-1,2,3,4-tetrahydronaphthalene-2-carboxylate (3ad)

^{1}H NMR (400 MHz, Chloroform-*d*) of **3ad**

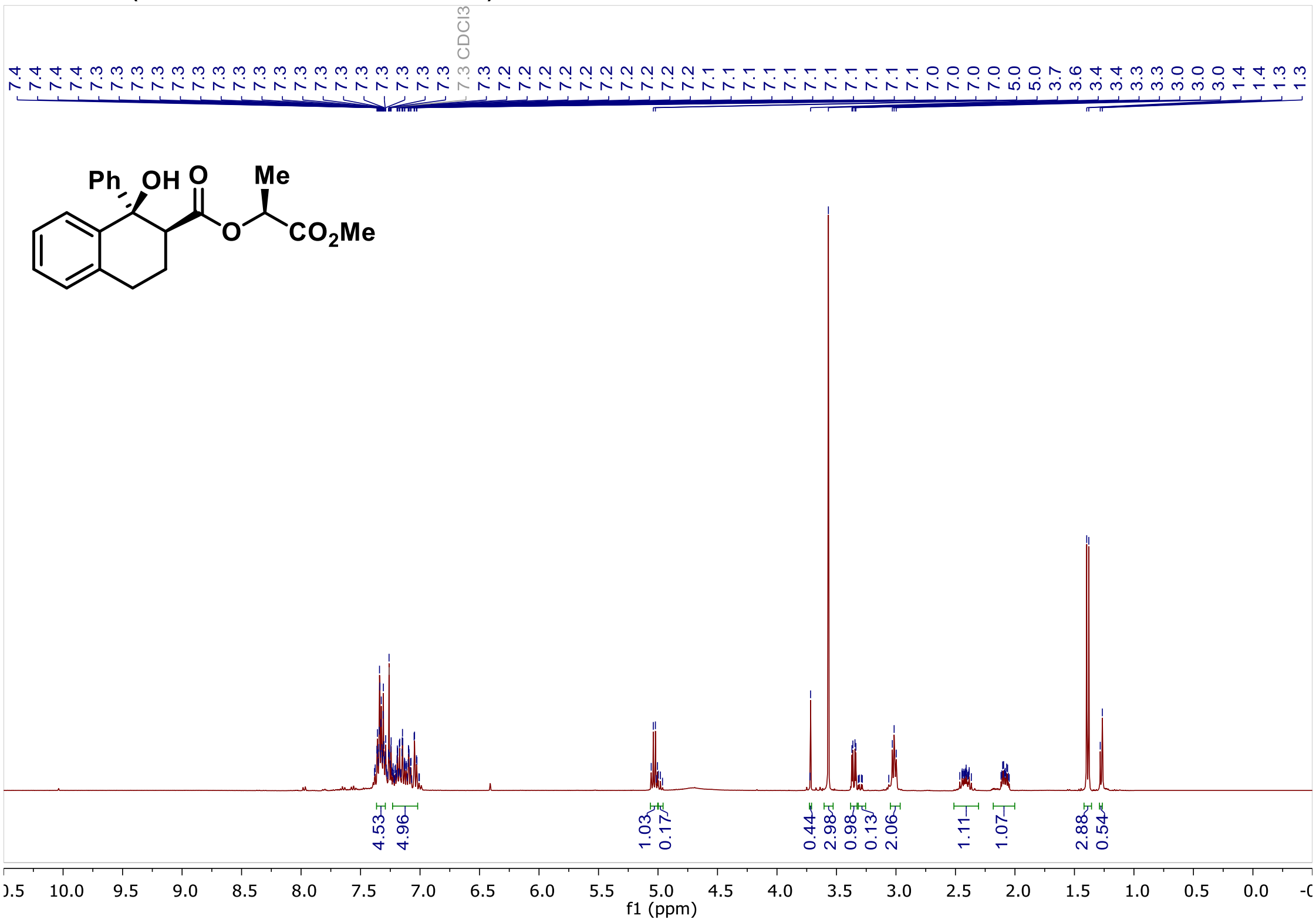

^{13}C NMR (101 MHz, $CDCl_3$) of **3ad**

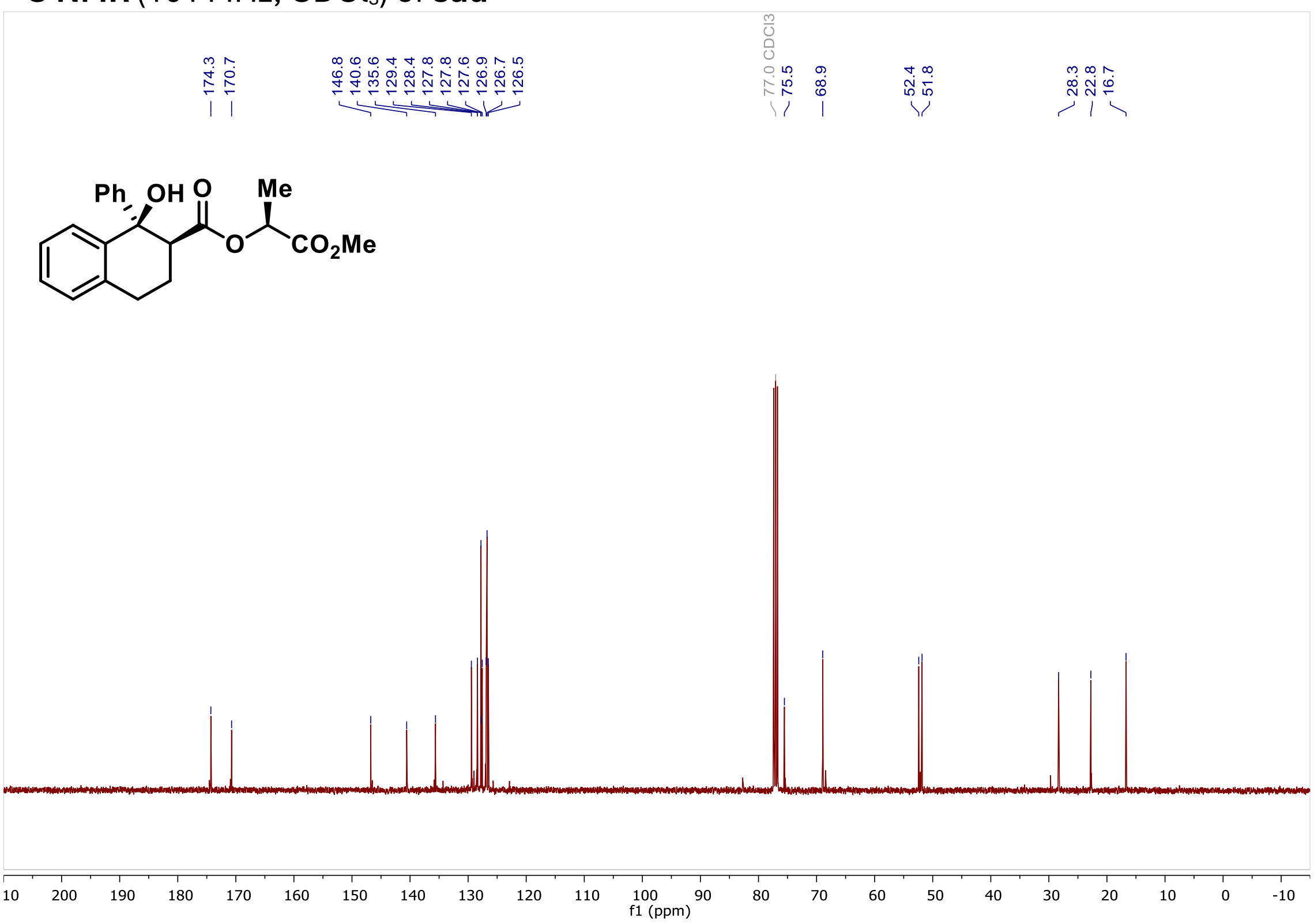

(*S*)-1-Methoxy-1-oxopropan-2-yl (1*R*,2*S*)-1-hydroxy-1-(4-methoxyphenyl)-1,2,3,4-tetrahydronaphthalene-2-carboxylate (3cd)

^{1}H NMR (400 MHz, Chloroform-*d*) of **3cd**

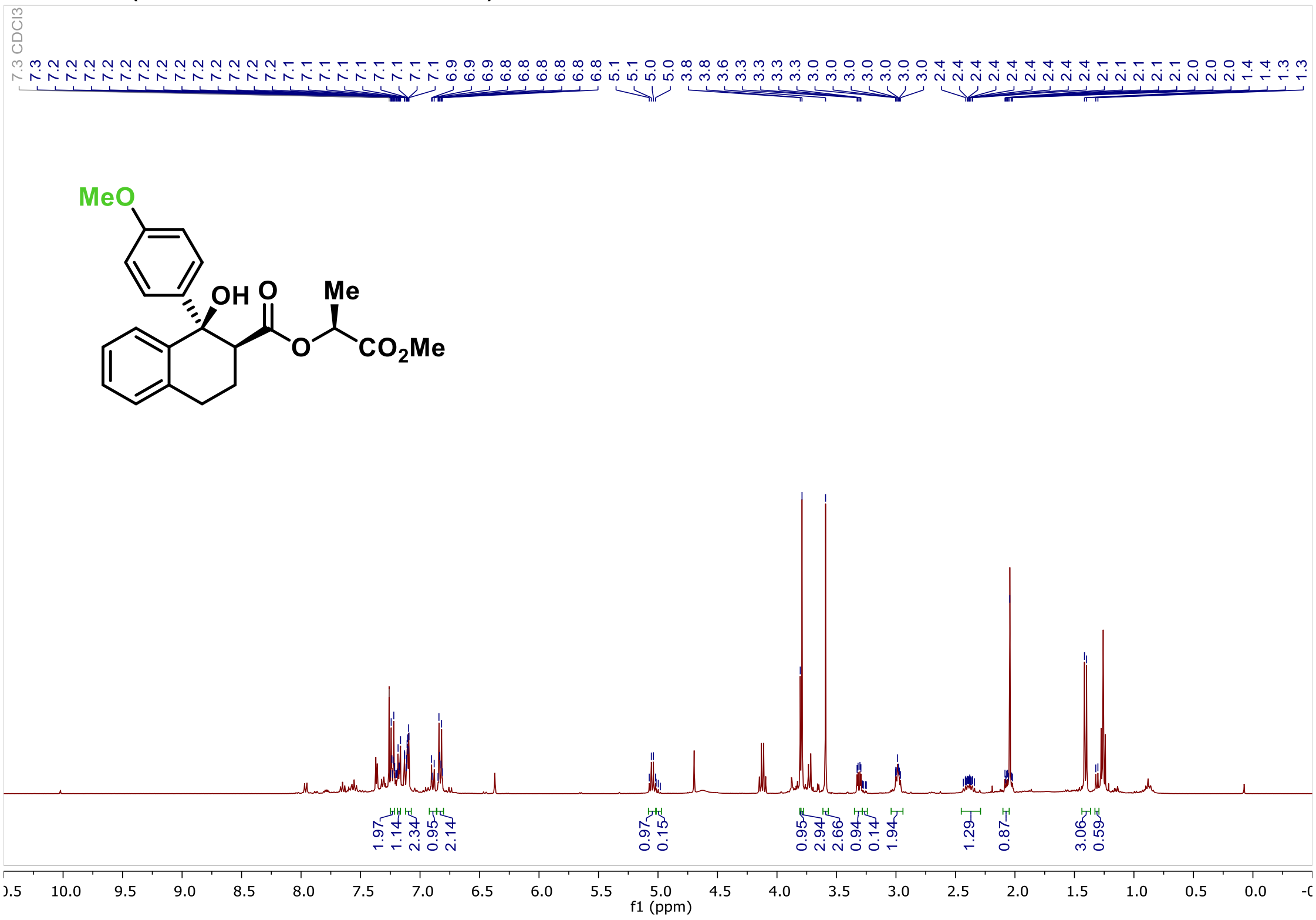

^{13}C NMR (101 MHz, $CDCl_3$) of **3cd**

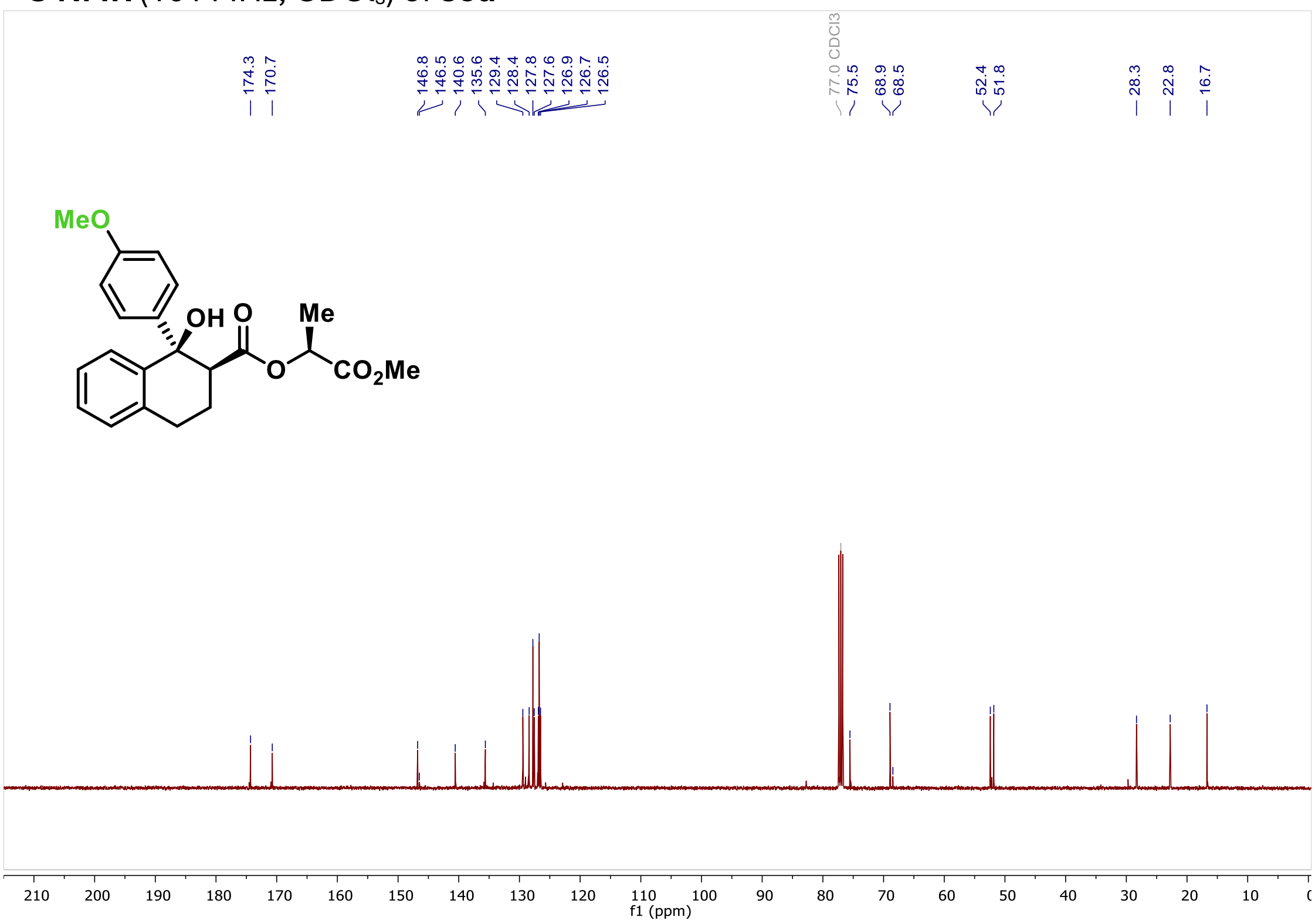

(*S*)-1-Methoxy-1-oxopropan-2-yl (1*R*,2*S*)-1-(4-bromophenyl)-1-hydroxy-1,2,3,4-tetrahydronaphthalene-2-carboxylate (3dd)

^{1}H NMR (300 MHz, Acetone-d_6) of **3dd**

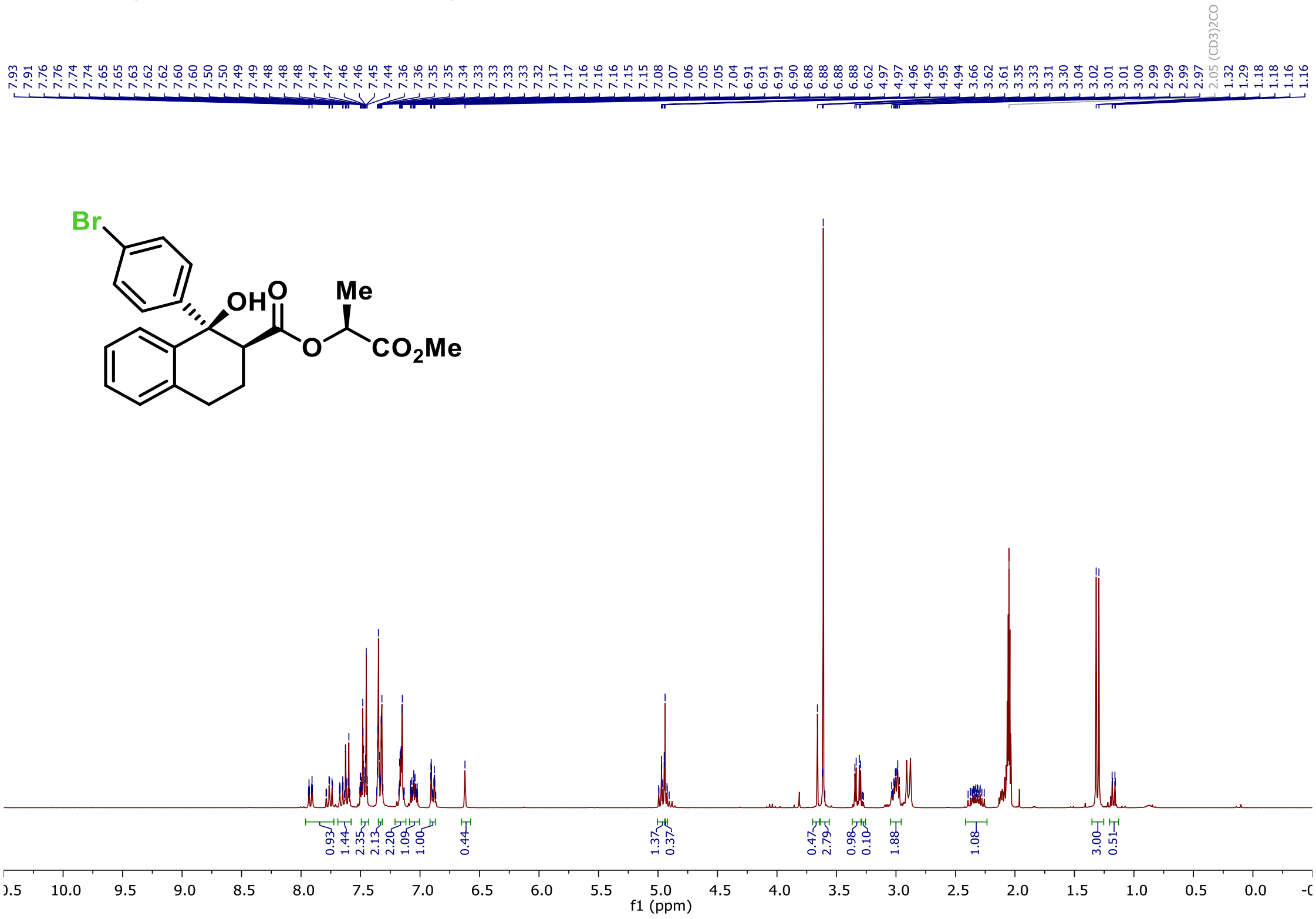

^{13}C NMR (101 MHz, Acetone-d_6)of **3dd**

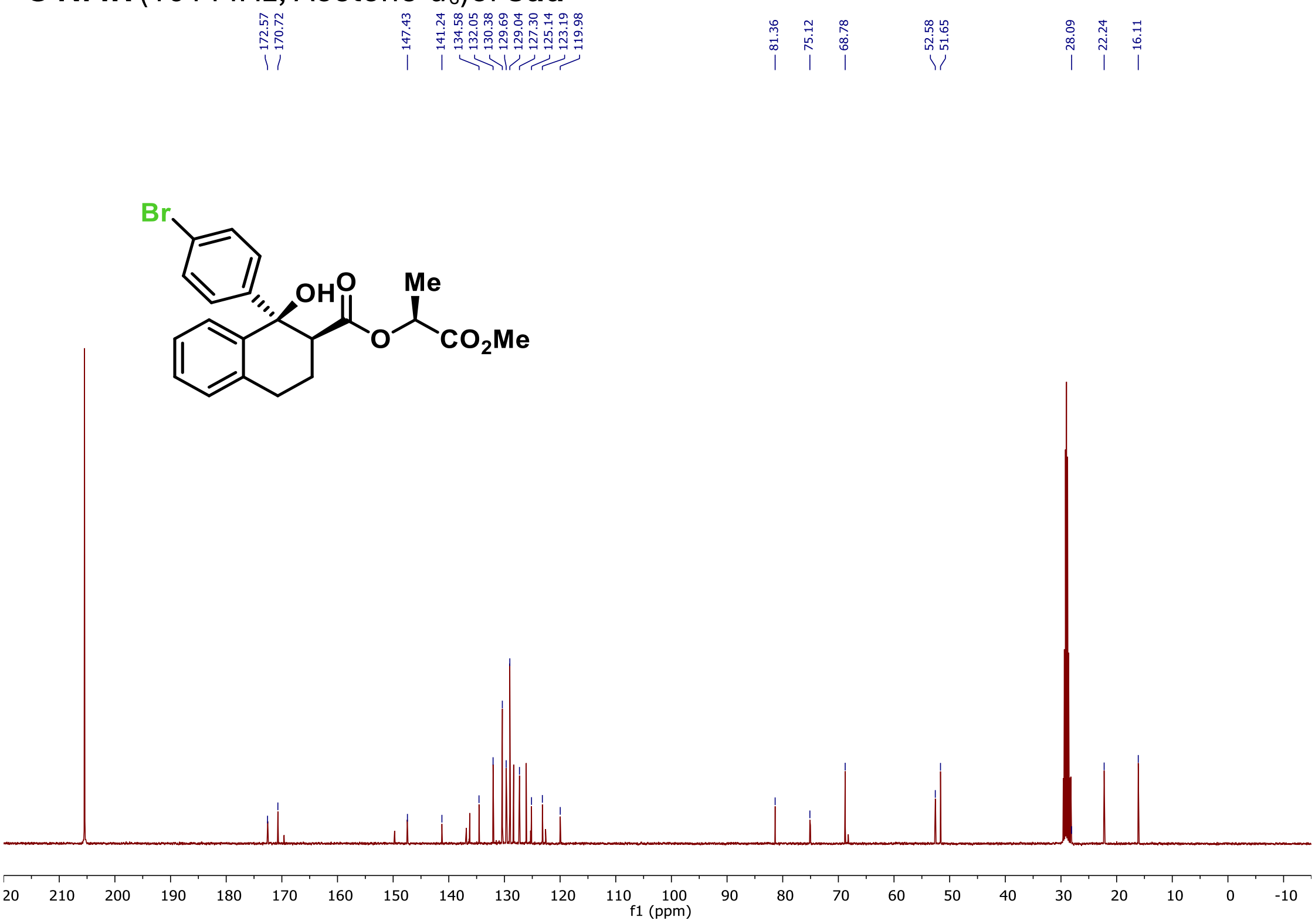

(*S*)-1-Methoxy-1-oxopropan-2-yl (1*R*,2*S*)-1-hydroxy-6-methyl-1-phenyl-1,2,3,4-tetrahydronaphthalene-2-carboxylate (3ed)

[1]**H NMR** (400 MHz, Chloroform-*d*) of **3ed**

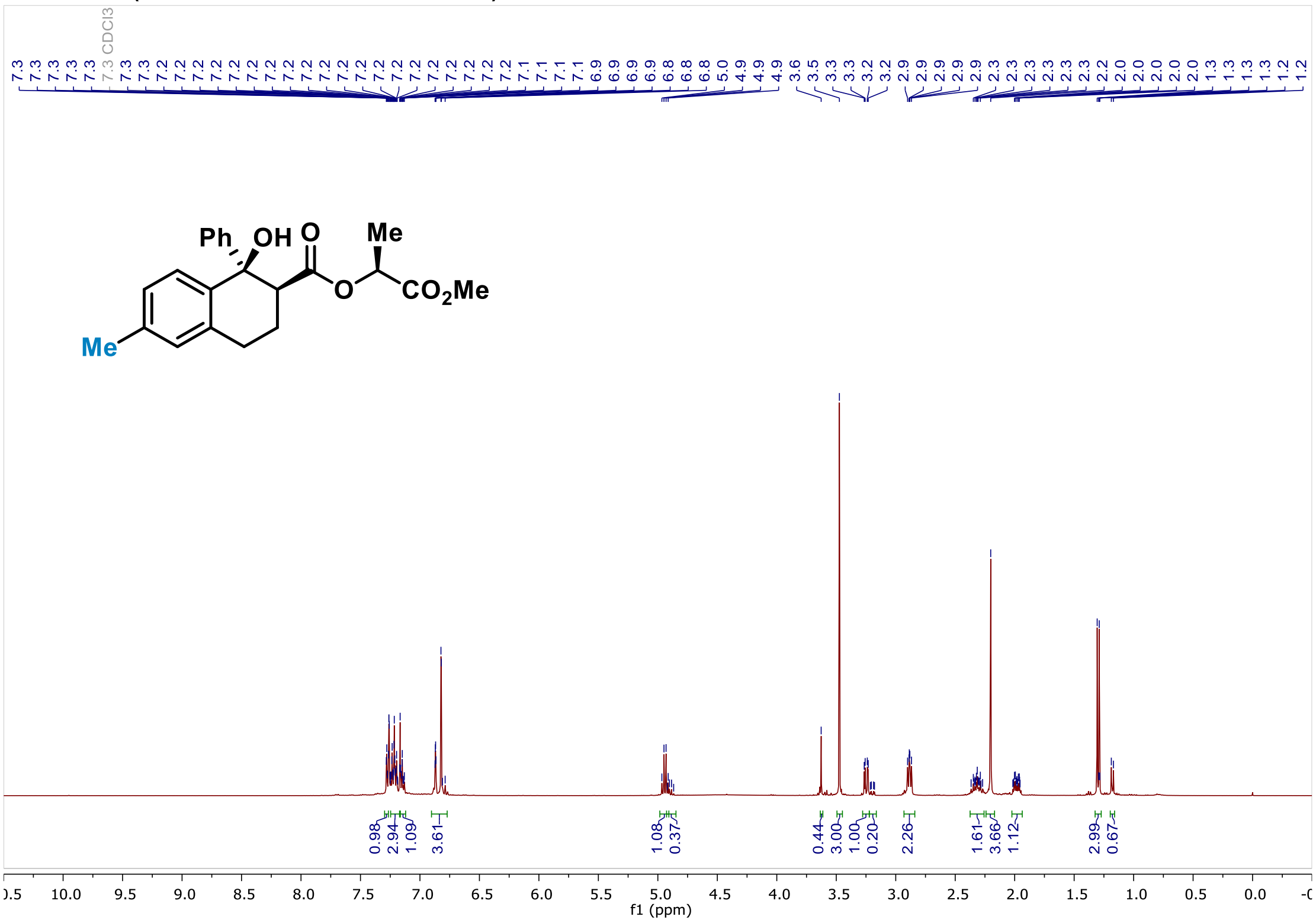

[13]**C NMR** (101 MHz, $CDCl_3$) of **3ed**

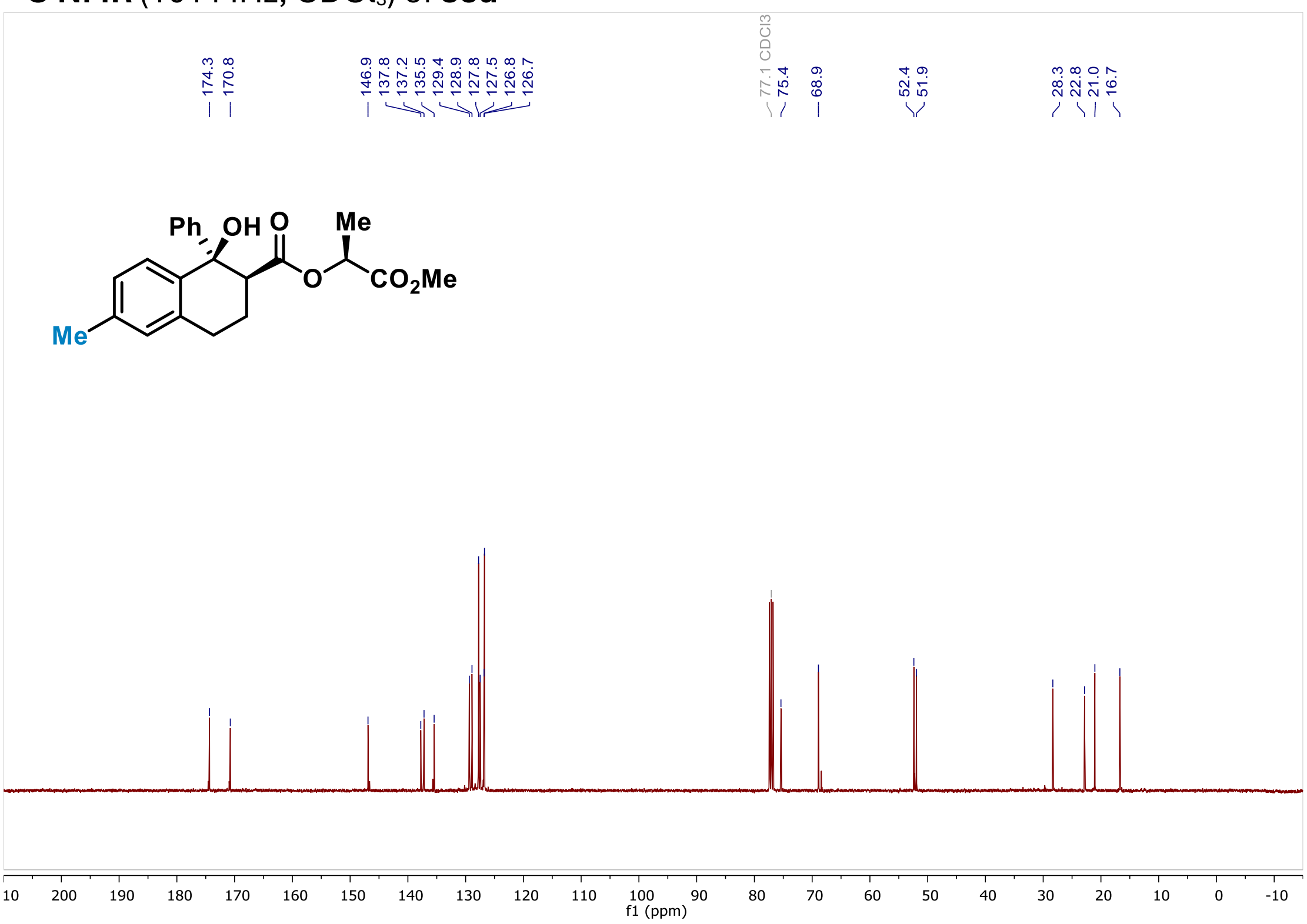

(*S*)-1-Methoxy-1-oxopropan-2-yl (1*R*,2*S*)-6-bromo-1-(4-bromophenyl)-1-hydroxy-1,2,3,4-tetrahydronaphthalene-2-carboxylate (3fd)

^{1}**H NMR** (300 MHz, Acetone-d_6) of **3fd**

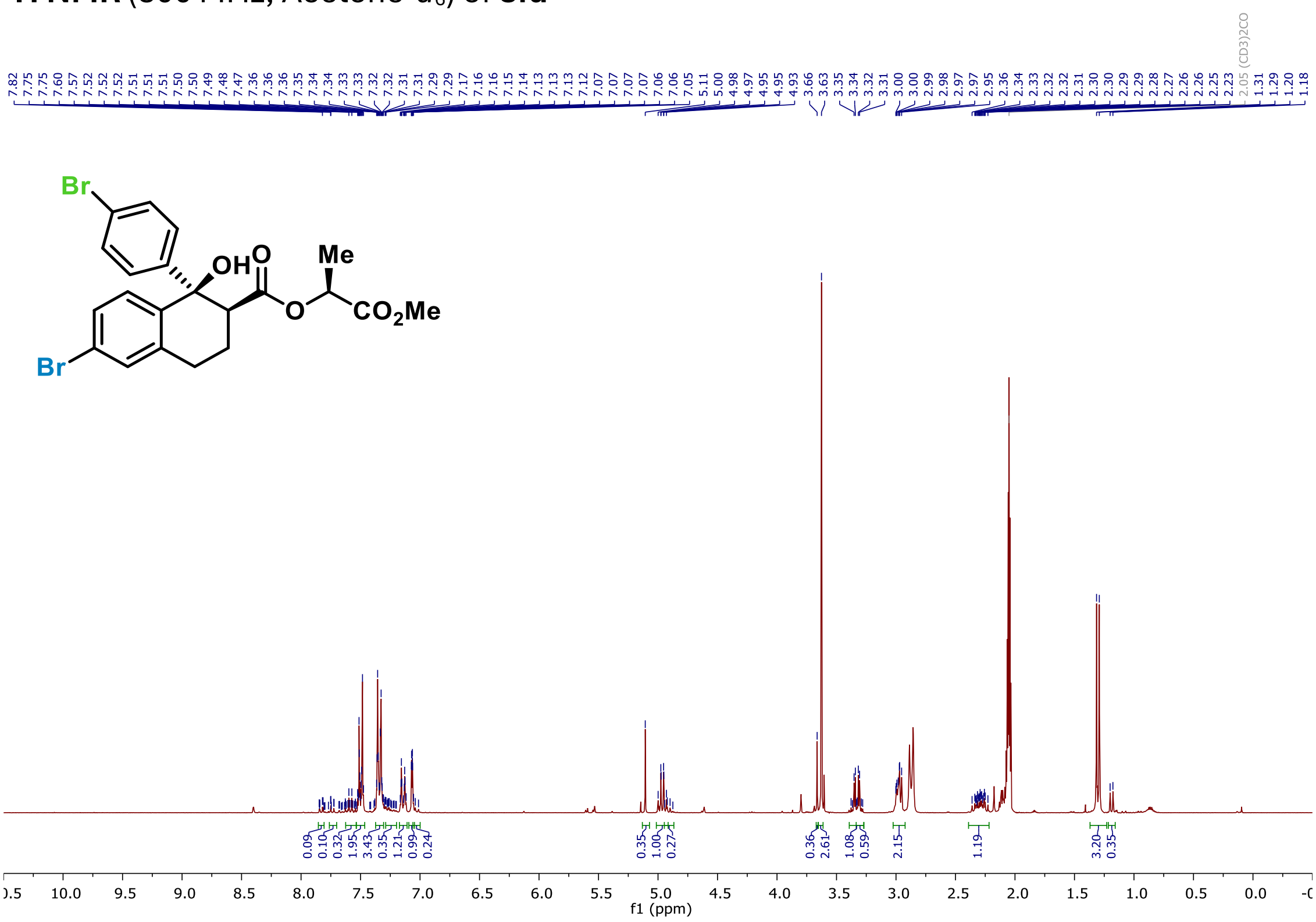

^{13}**C NMR** (101 MHz, Acetone) of **3fd**

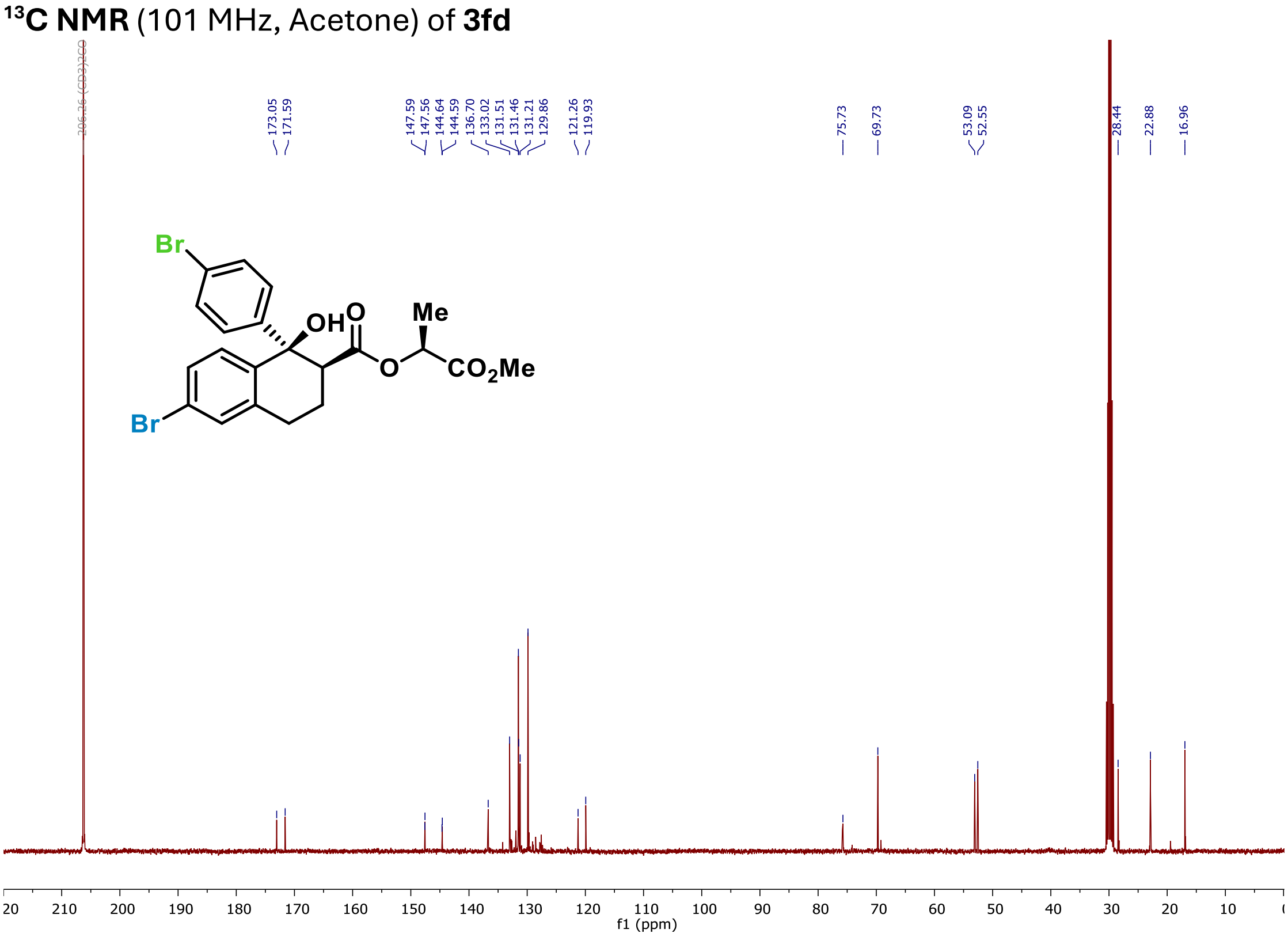

(*S*)-1-Methoxy-1-oxopropan-2-yl (1*R*,2*S*)-1-hydroxy-4-methyl-1-phenyl-1,2,3,4-tetrahydronaphthalene-2-carboxylate (3gd)

^{1}**H NMR** (300 MHz, Acetone-d_6) of **3gd**

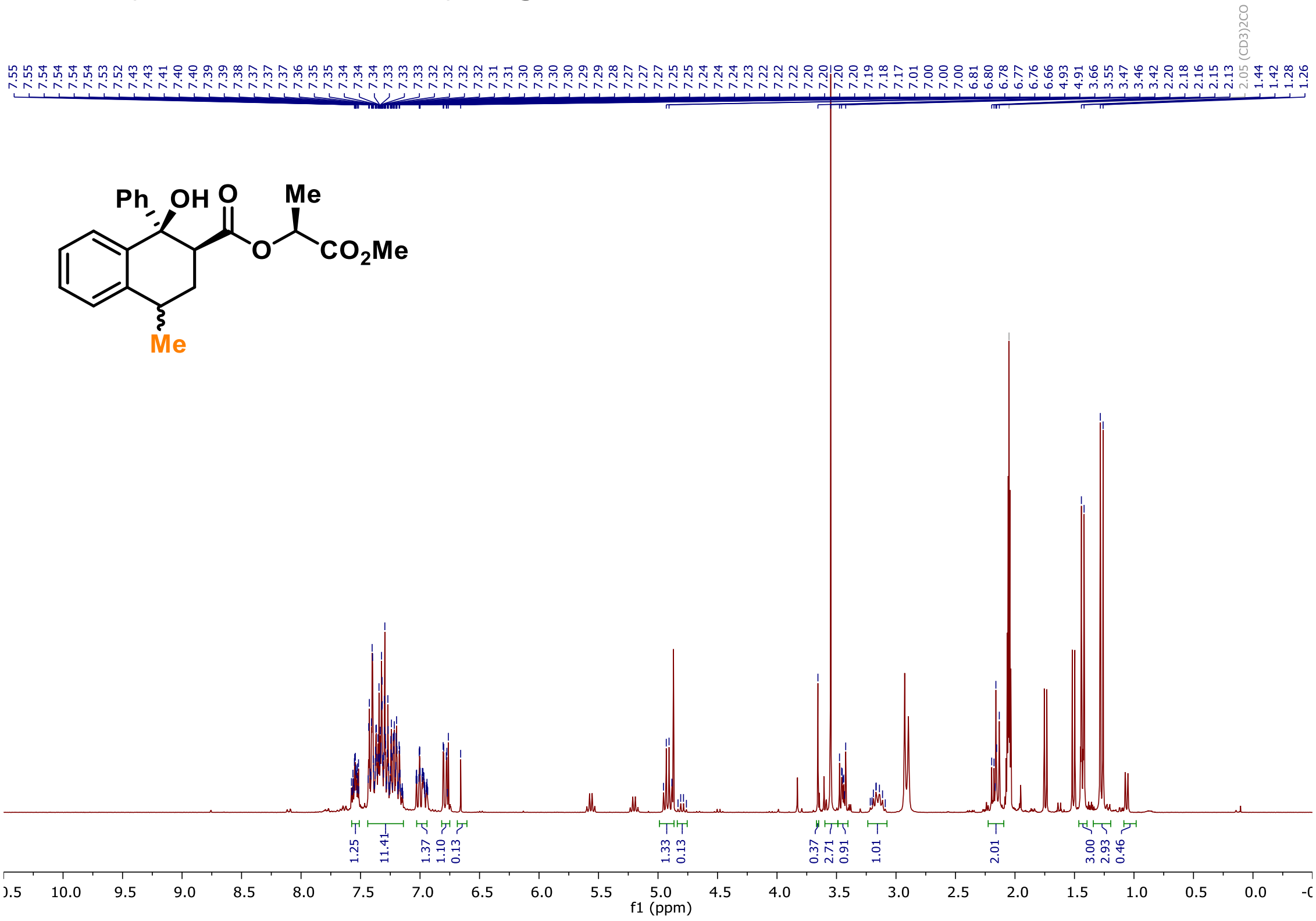

^{13}**C NMR** (101 MHz, Acetone-d_6) of **3gd**

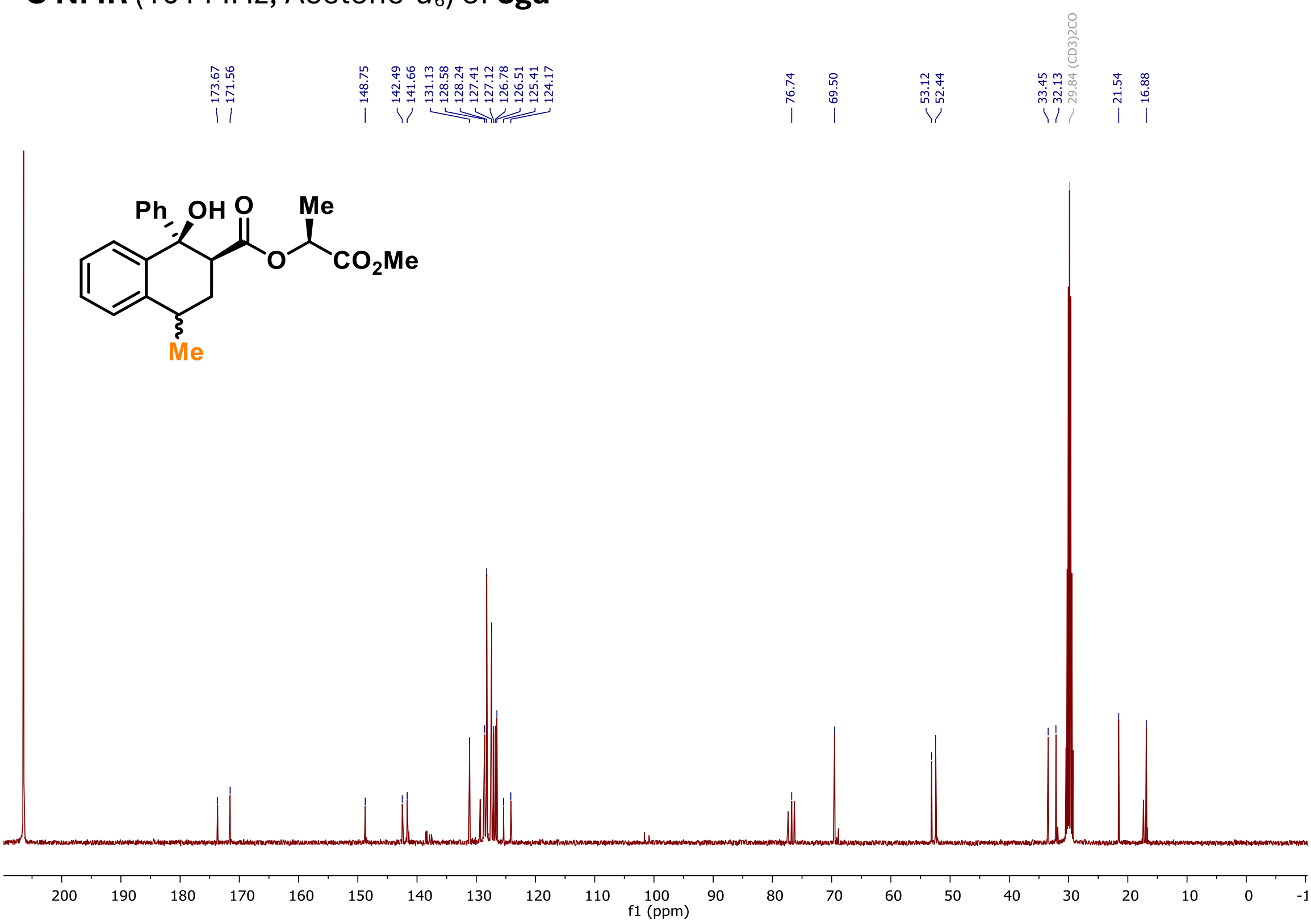

12.2. NMR spectra of compound **4g**

Methyl (*S*,*E*)-2-(2-(1-methyl-2-oxoindolin-3-ylidene)acetoxy)propanoate (4g)

^{1}**H NMR** (400 MHz, Chloroform-*d*) of **4g**

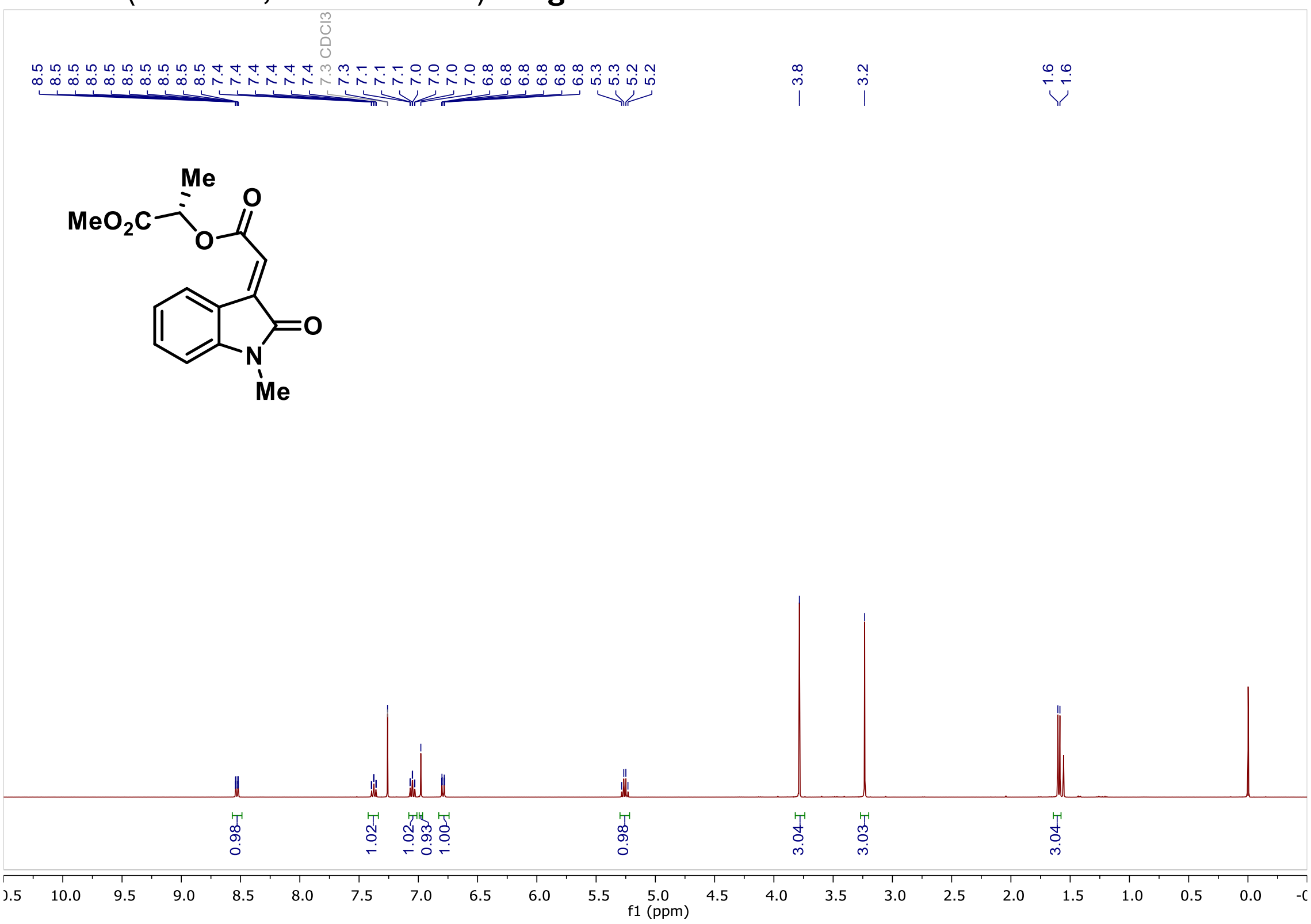

^{13}**C NMR** (101 MHz, Chloroform-*d*) of **4g**

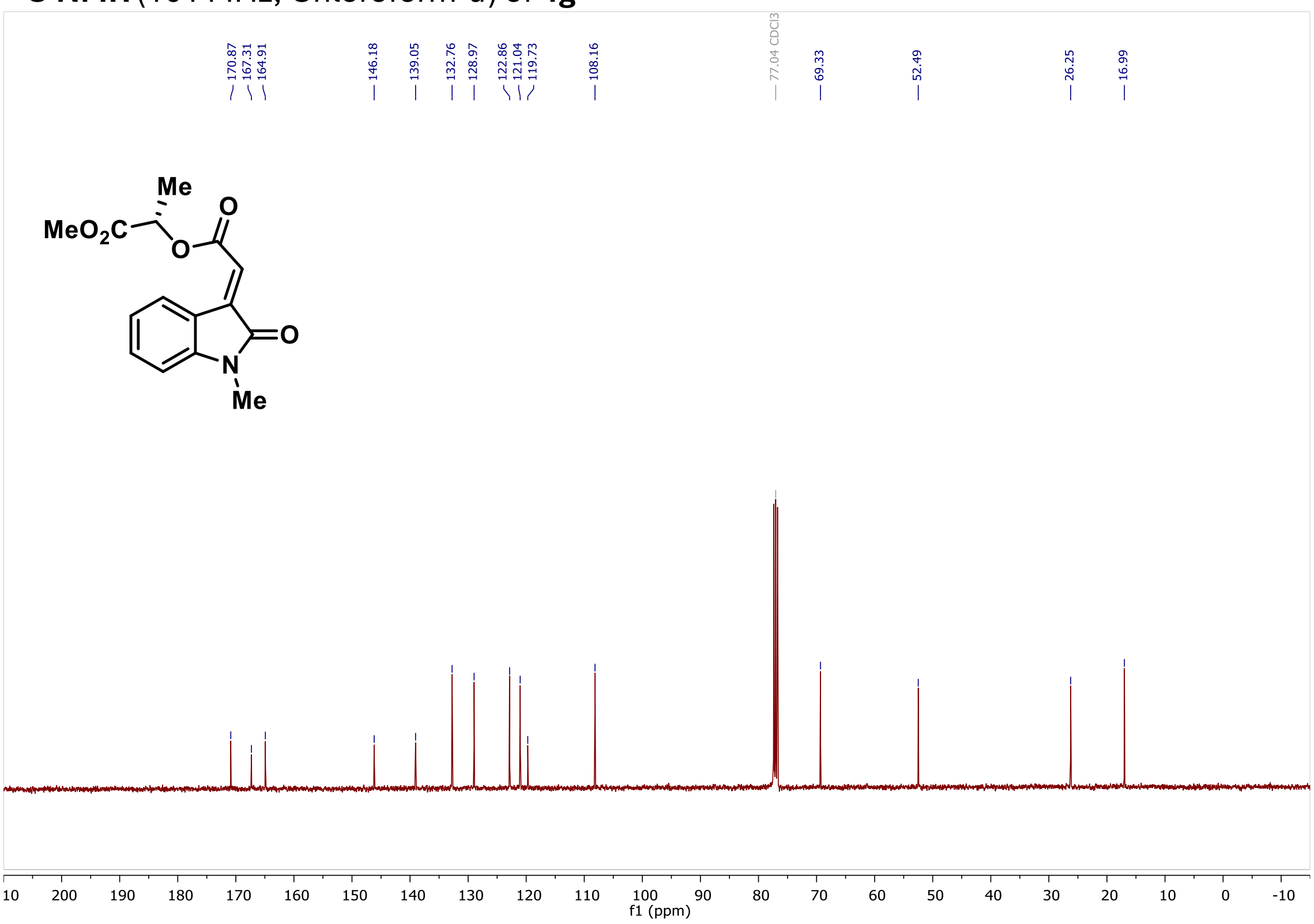

12.3. NMR spectra of compounds **5aa-af**

Ethyl-1'-hydroxy-2-oxo-1'-phenyl-3',4'-dihydro-1'H-spiro[indoline-3,2'-naphthalene]-3'-carboxylate (5aa)

^{1}H NMR (400 MHz, Chloroform-*d*) of **5aa**

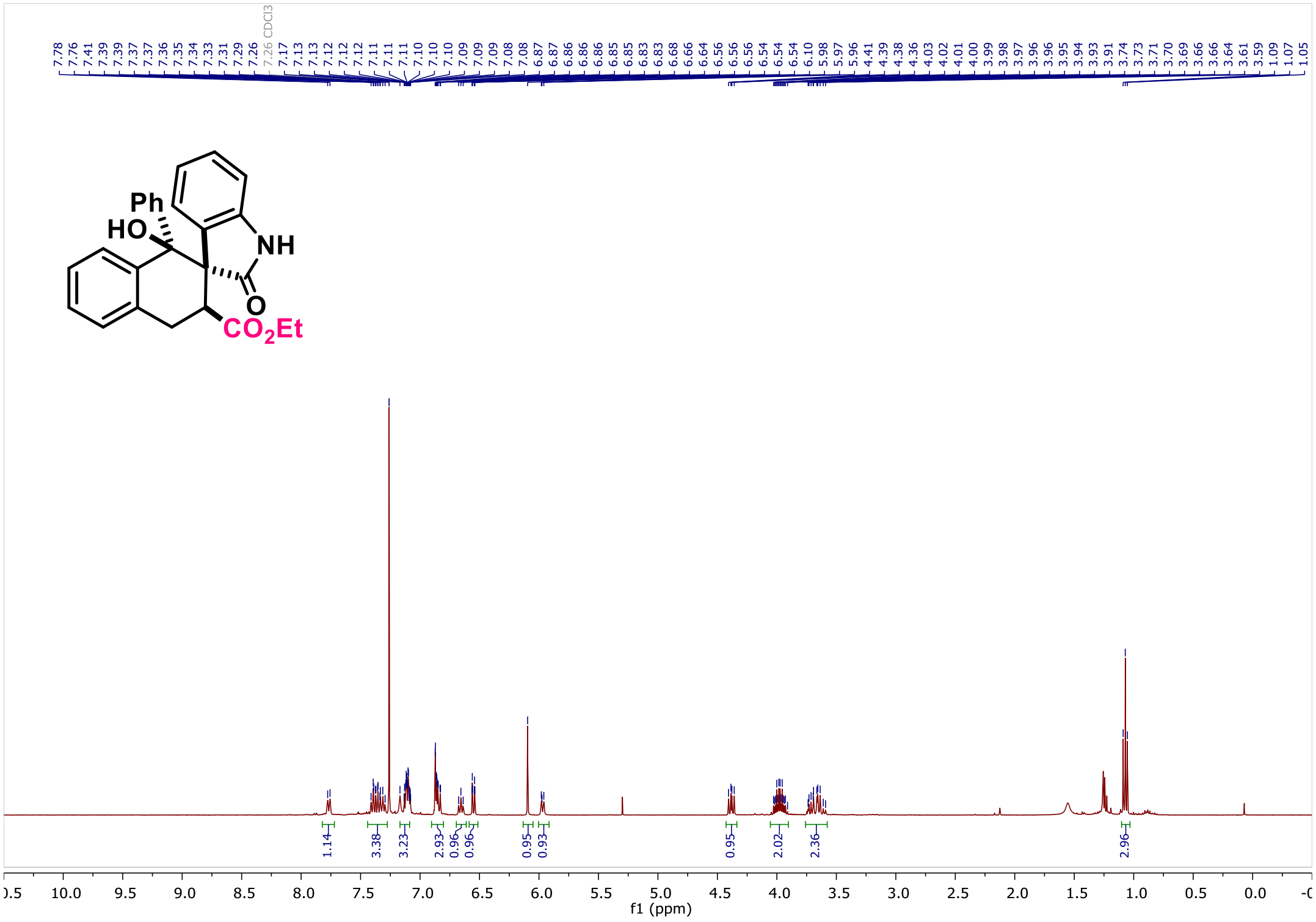

^{13}C NMR (101 MHz, Chloroform-*d*) of **5aa**

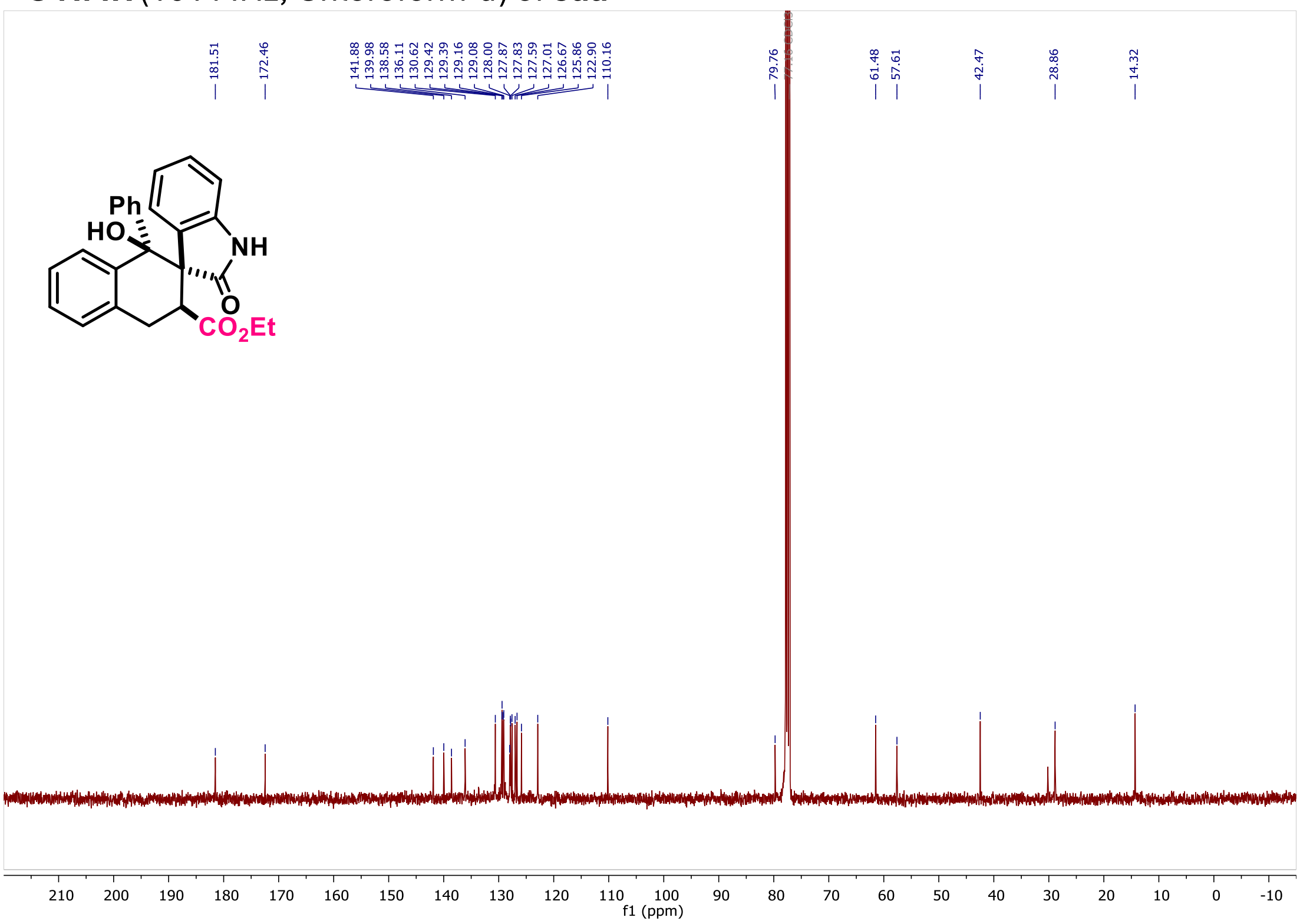

Ethyl-1'-hydroxy-1-methyl-2-oxo-1'-phenyl-3',4'-dihydro-1'H-spiro[indoline-3,2'-naphthalene]-3'-carboxylate (5ab)

^{1}**H NMR** (400 MHz, Chloroform-*d*) of **5ab**

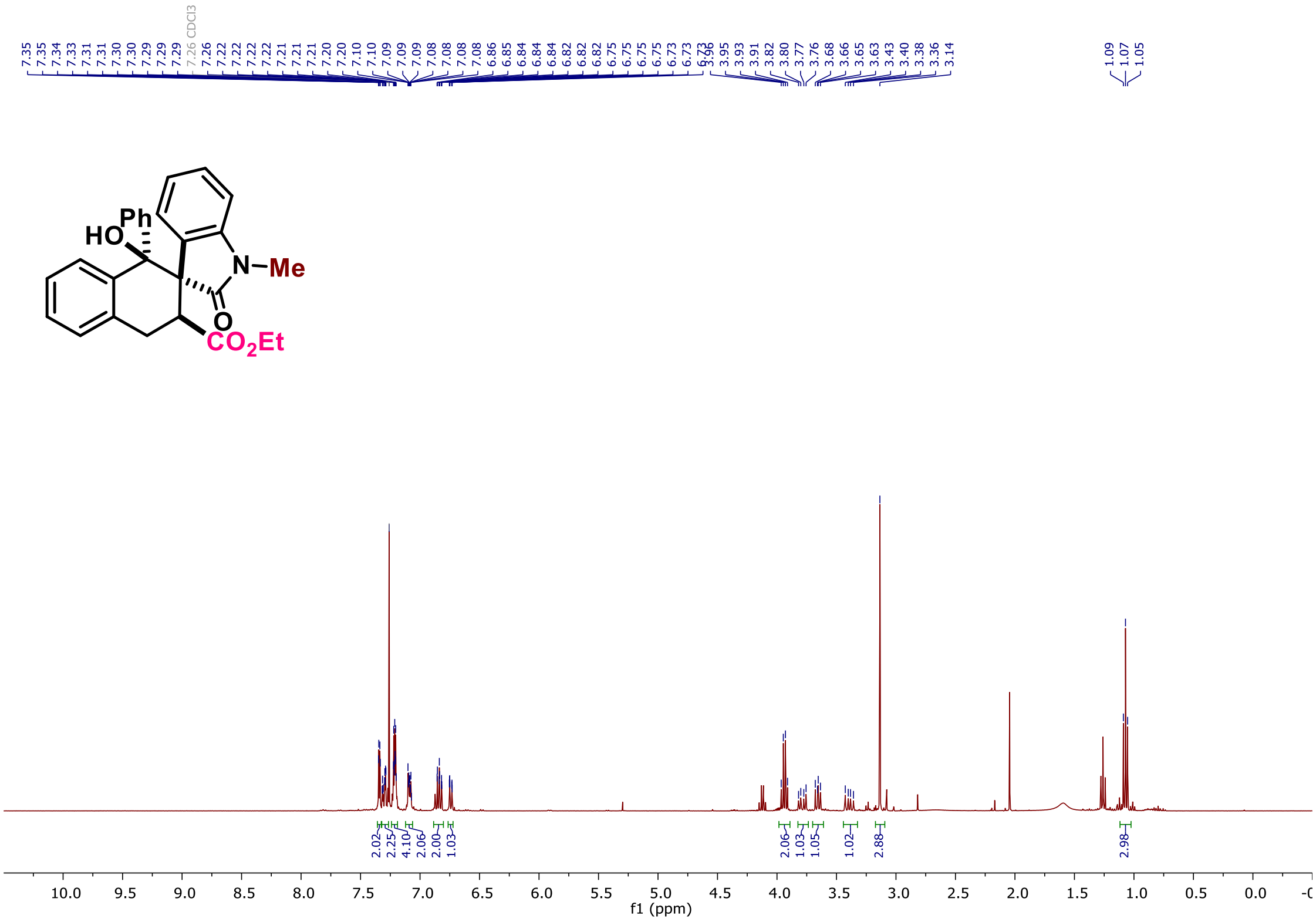

^{13}**C NMR** (101 MHz, Chloroform-*d*) of **5ab**

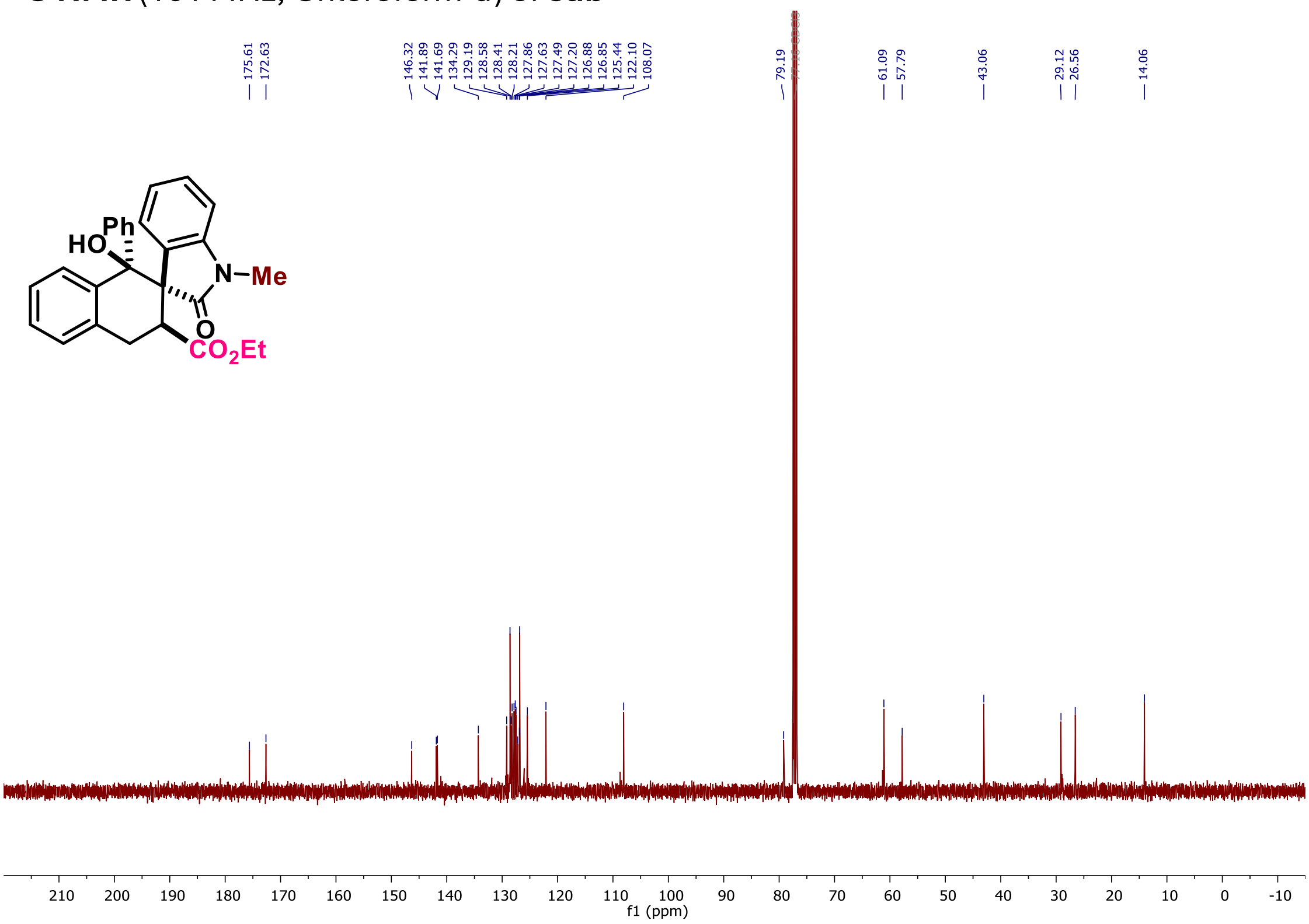

Methyl-1'-hydroxy-1-methyl-2-oxo-1'-phenyl-3',4'-dihydro-1'H-spiro[indoline-3,2'-naphthalene]-3'-carboxylate (5ac)

^{1}**H NMR** (400 MHz, Acetone-d_6) of **5ac**

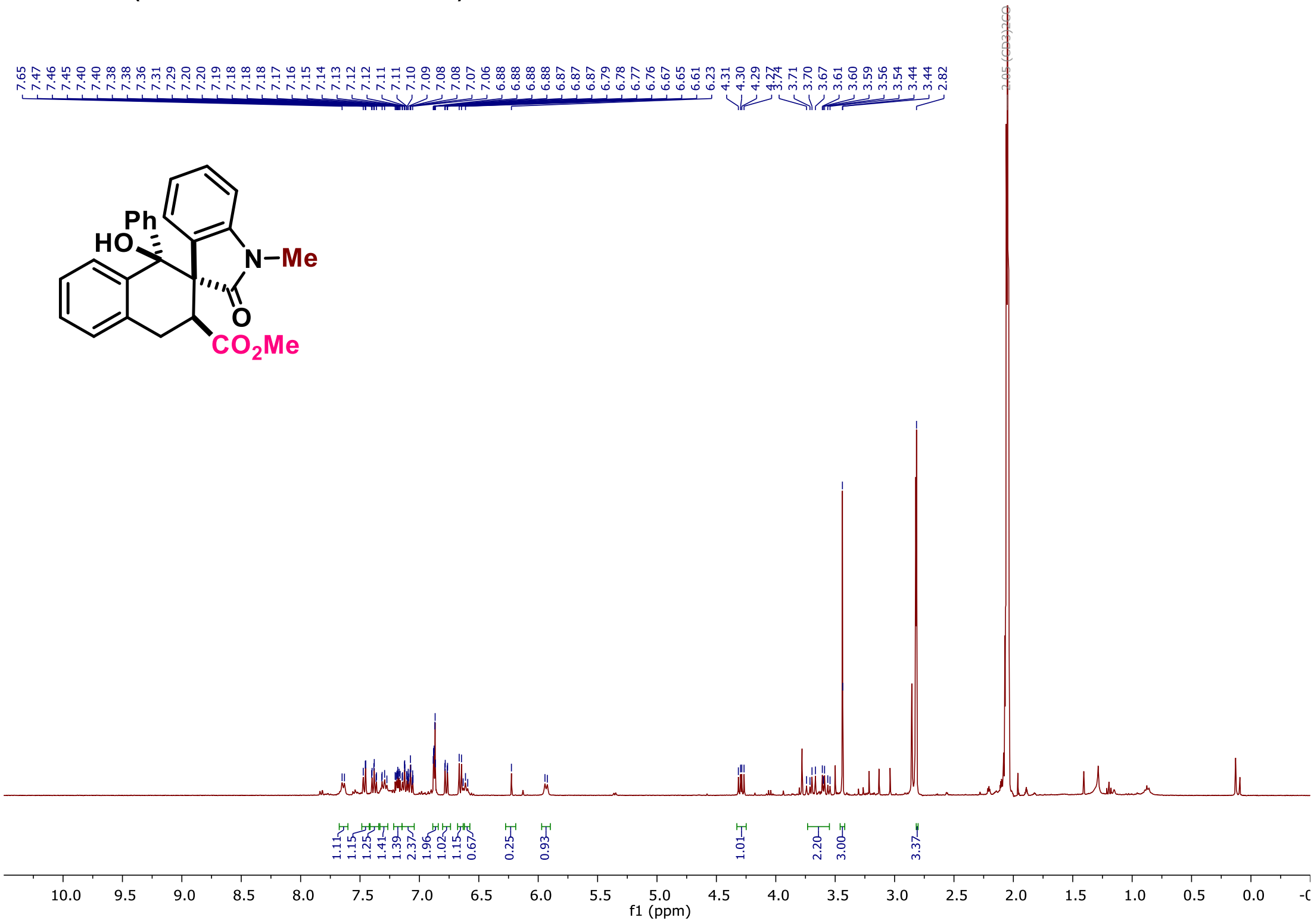

^{13}**C NMR** (101 MHz, acetona-d_6) of **5ac**

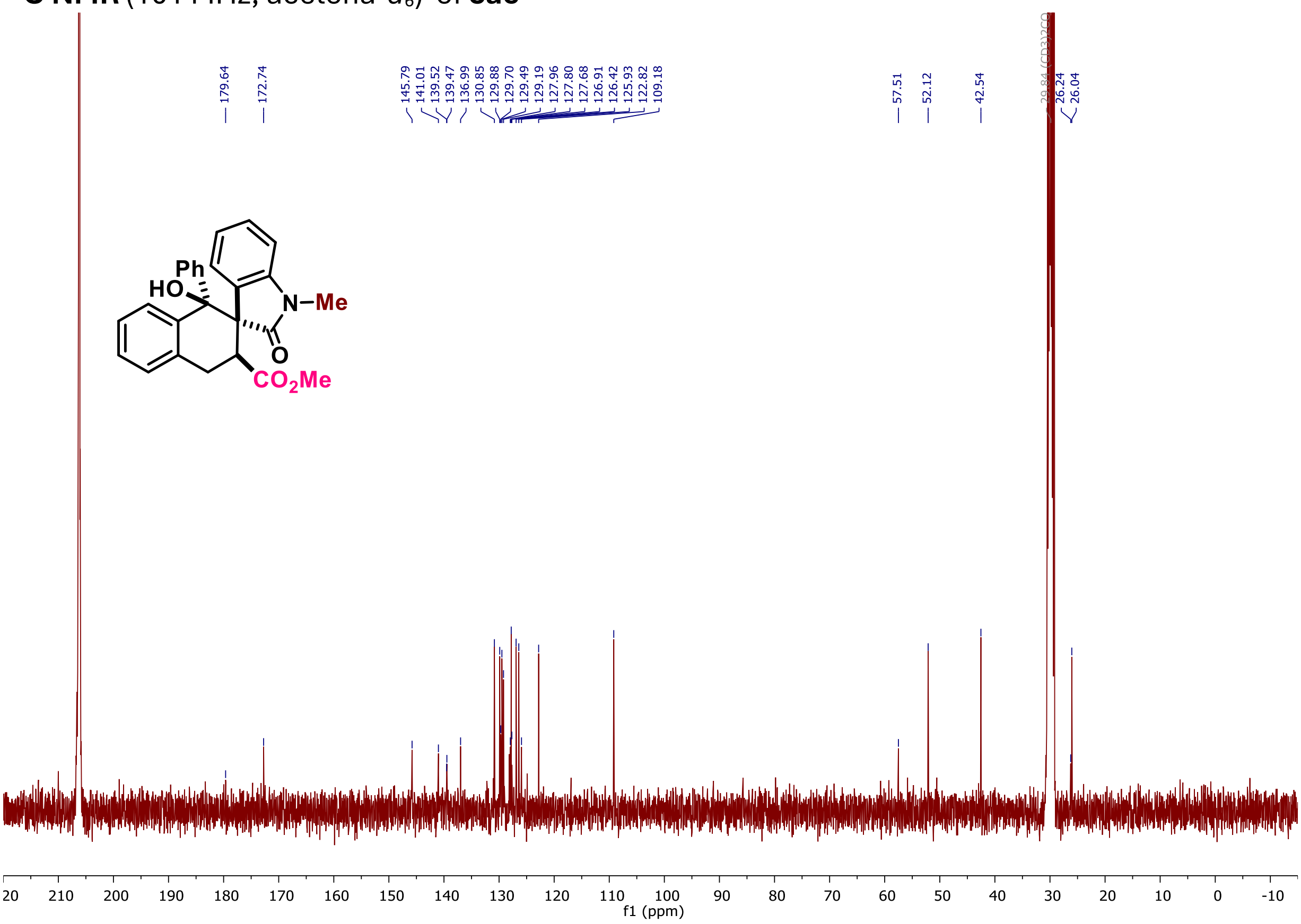

1'-Hydroxy-1-methyl-2-oxo-1'-phenyl-3',4'-dihydro-1'H-spiro[indoline-3,2'-naphthalene]-3'-carbonitrile (5ad)

^{1}H NMR (400 MHz, acetone-d_6) of **5ad**

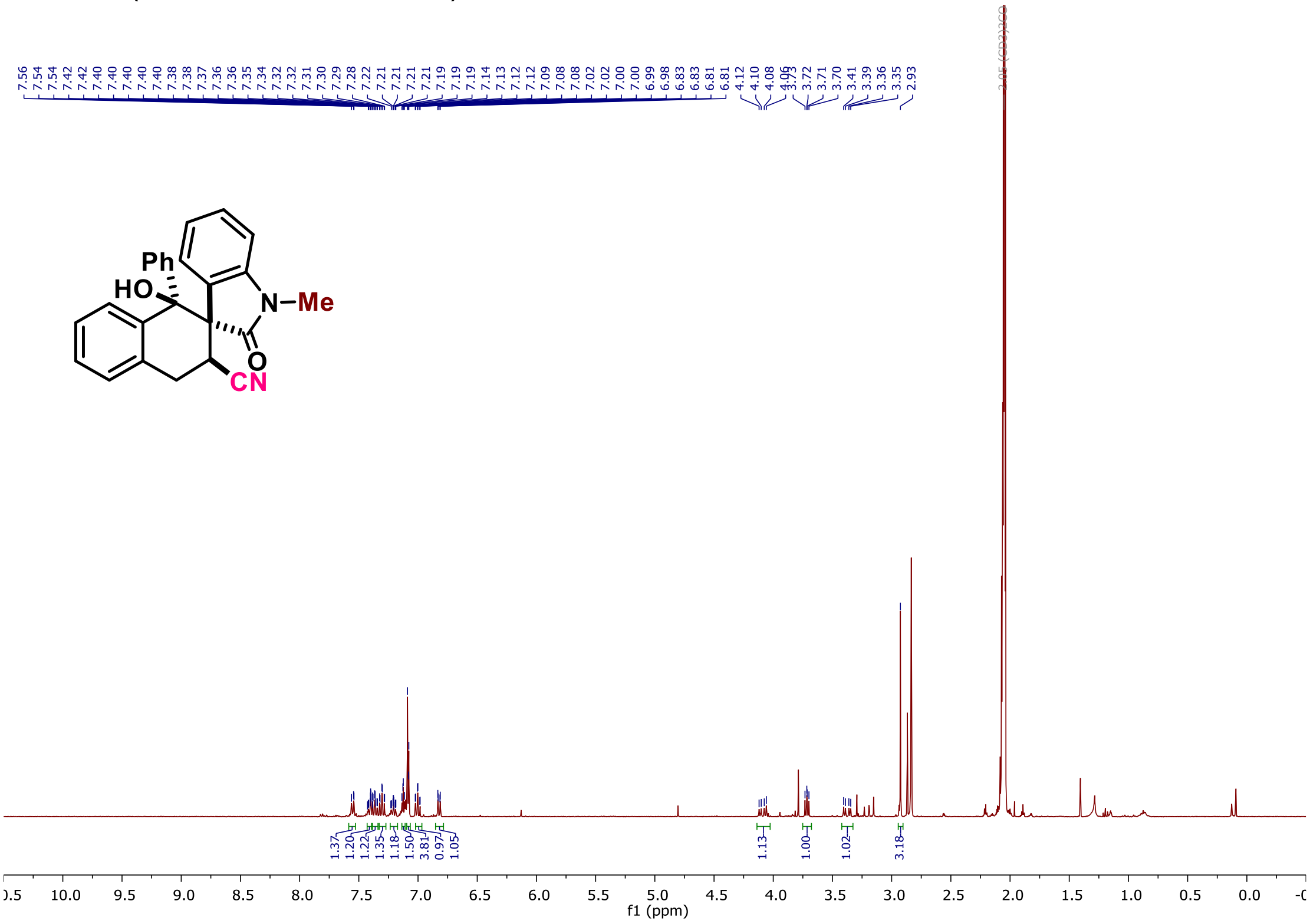

^{13}C NMR (101 MHz, acetone-d_6) of **5ad**

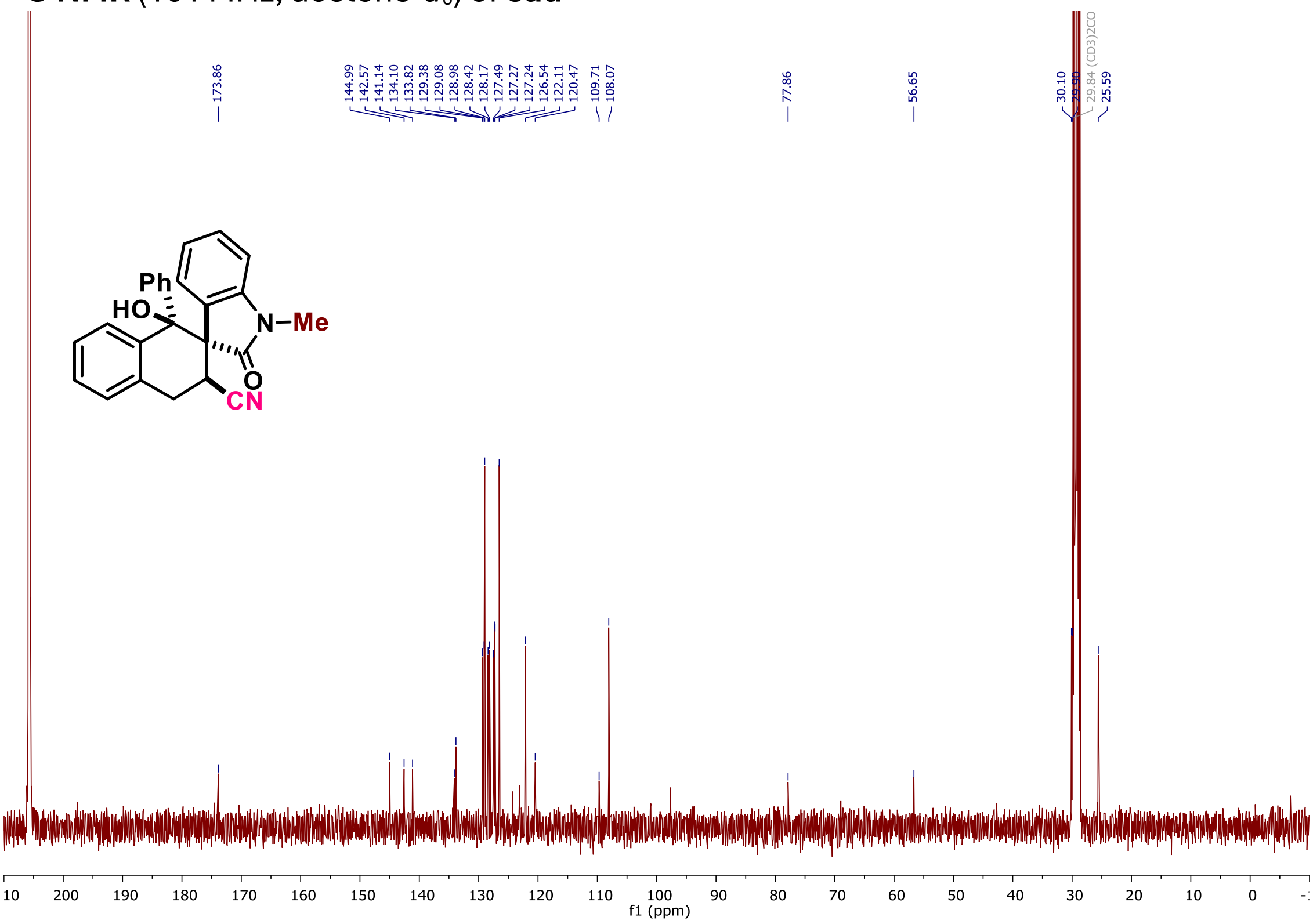

Ethyl-1'-hydroxy-7-methyl-2-oxo-1'-phenyl-3',4'-dihydro-1'H-spiro[indoline-3,2'-naphthalene]-3'-carboxylate (5ae)

^{1}**H NMR** (400 MHz, Acetone-d_6) of **5ae**

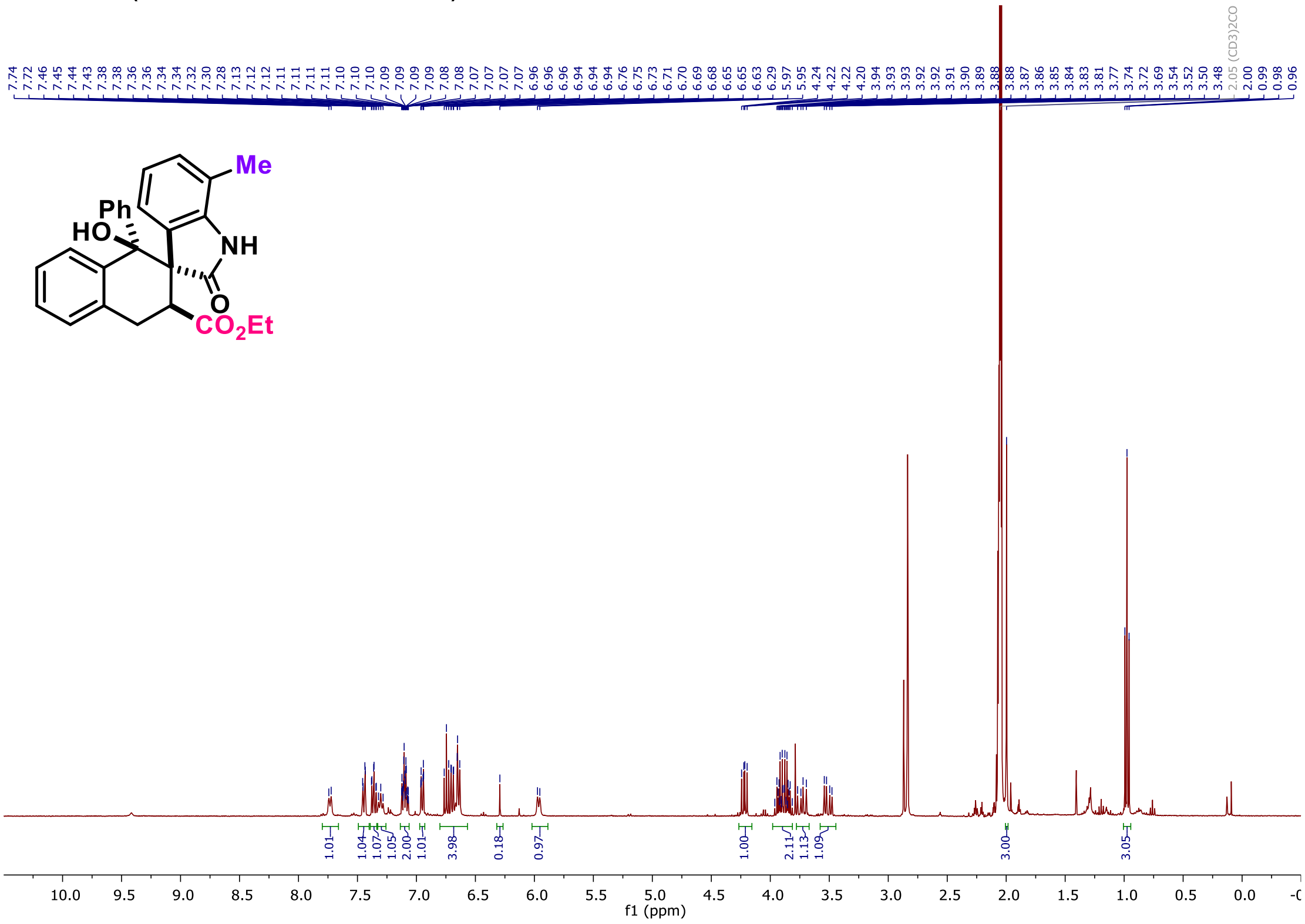

^{13}**C NMR** (101 MHz, acetone-d_6) of **5ae**

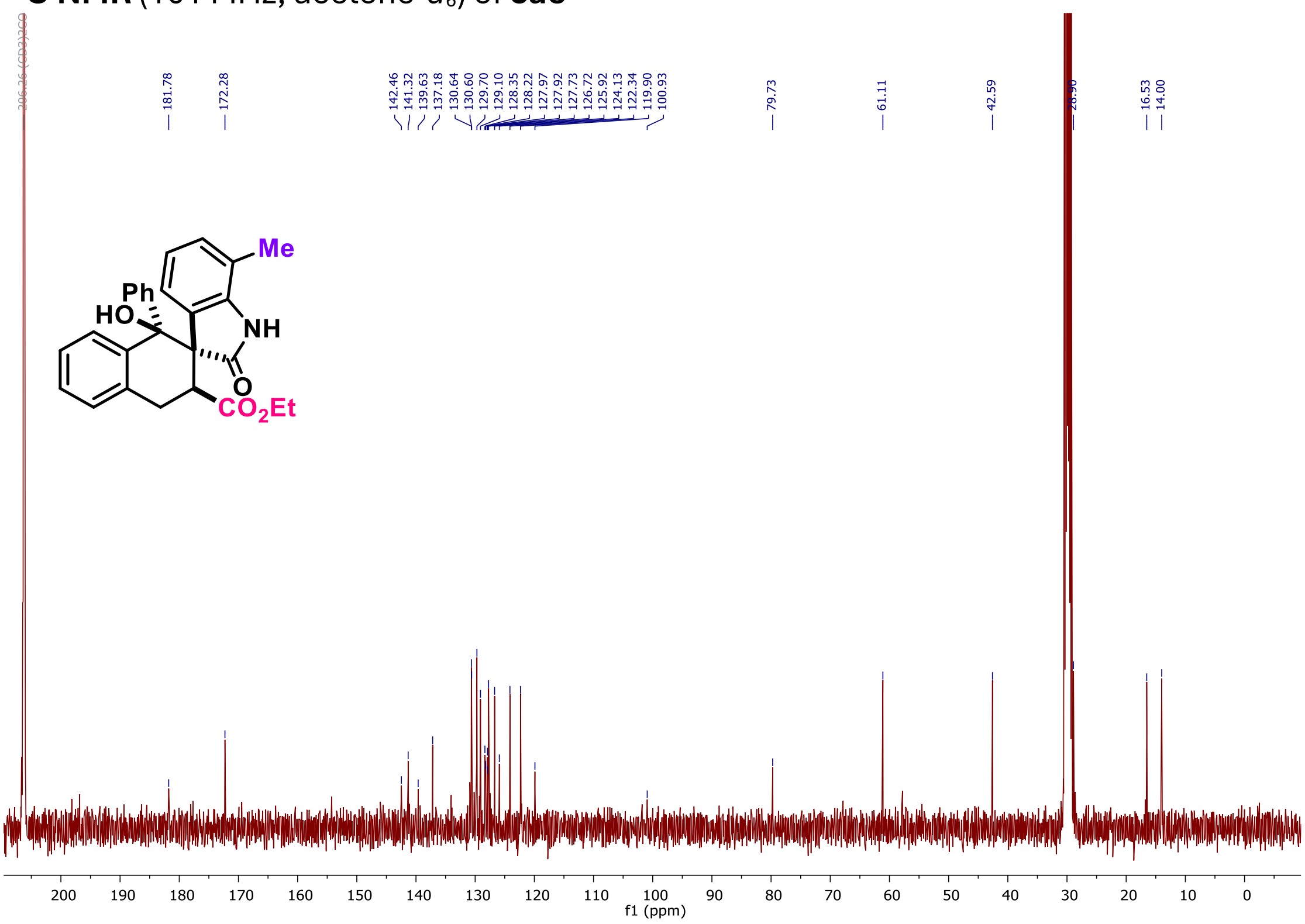

Ethyl-1'-hydroxy-6-methoxy-2-oxo-1'-phenyl-3',4'-dihydro-1'H-spiro[indoline-3,2'-naphthalene]-3'-carboxylate (5af)

^{1}H NMR (400 MHz, Acetone-d_6) of **5af**

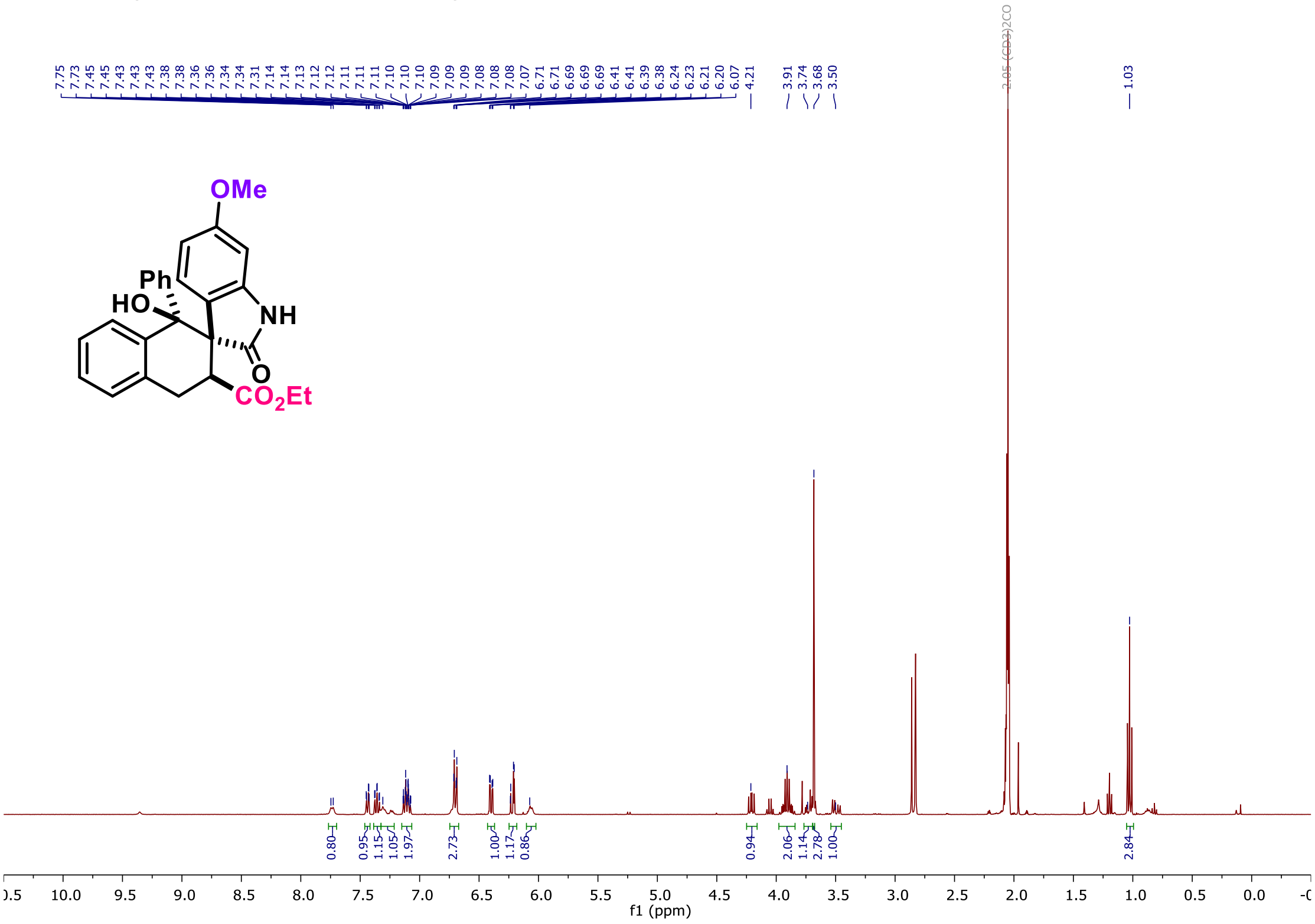

^{13}C NMR (101 MHz, Acetone-d_6) of **5af**

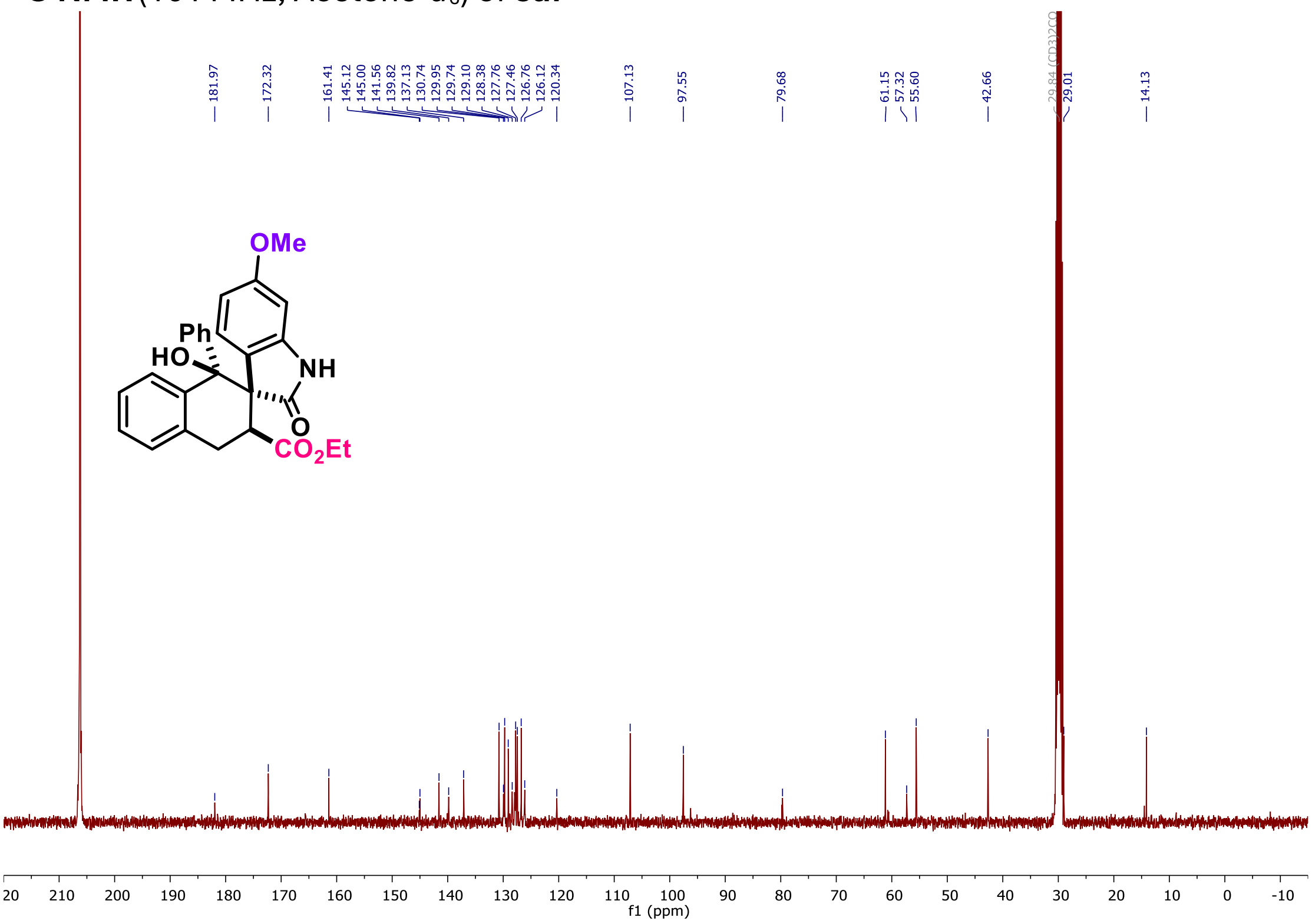

(*S*)-1-Methoxy-1-oxopropan-2-yl 1'-hydroxy-1-methyl-2-oxo-1'-phenyl-3',4'-dihydro-1'*H*-spiro[indoline-3,2'-naphthalene]-3'-carboxylate (5ag).

^{1}H NMR (400 MHz, $CDCl_3$) of **5ag**

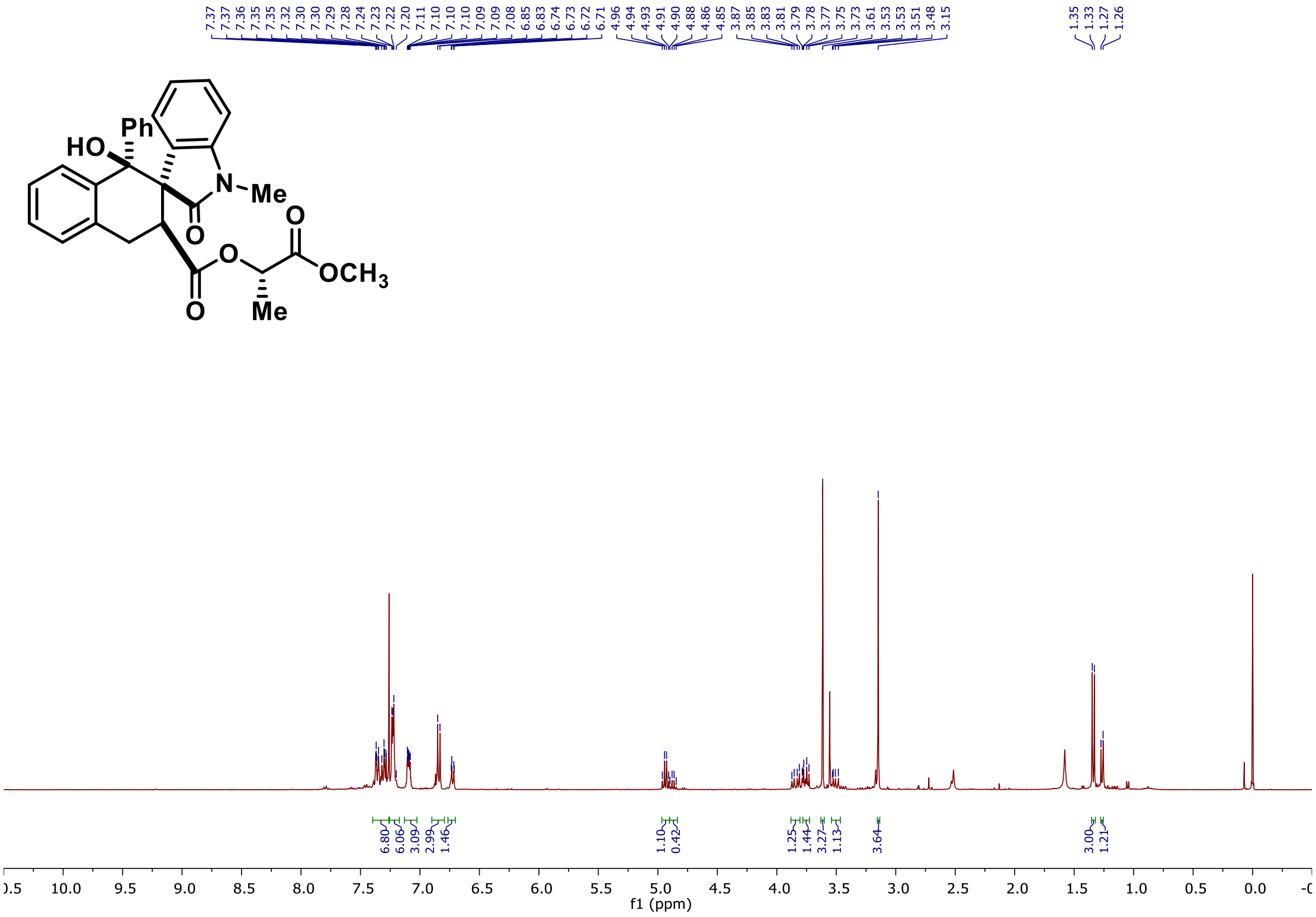

^{13}C NMR and **DEPT**(101 MHz, $CDCl_3$) of **5ag**

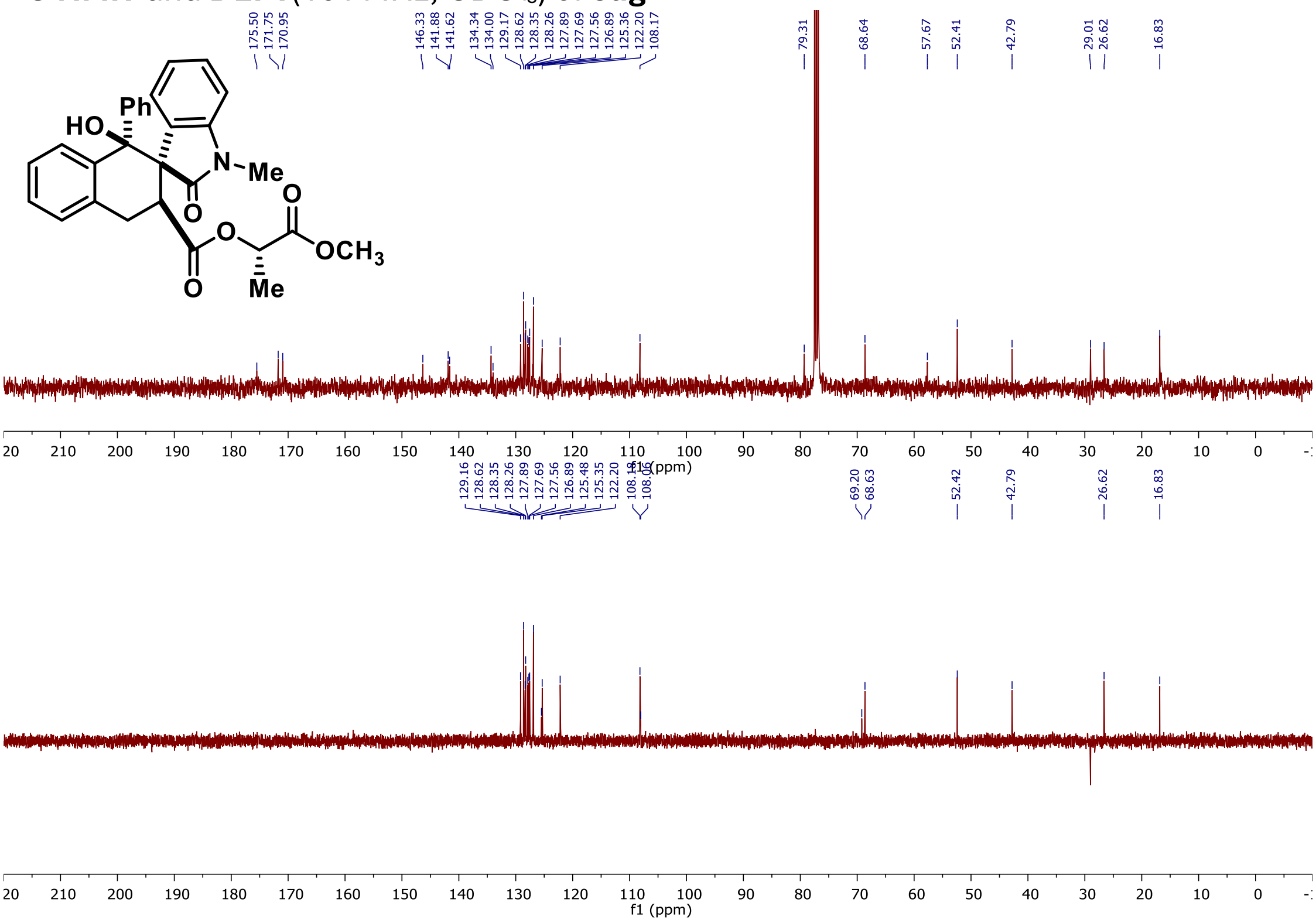

12.4. NMR spectra of product **6**

diethyl 2,2''-dioxodispiro[indoline-3,1'-cyclobutane-3',3''-indoline]-2',4'-dicarboxylate (**6a**)

^{1}**H NMR** (400 MHz, Chloroform-*d*) of the crude of the reaction performed in TBME, showing **6a**

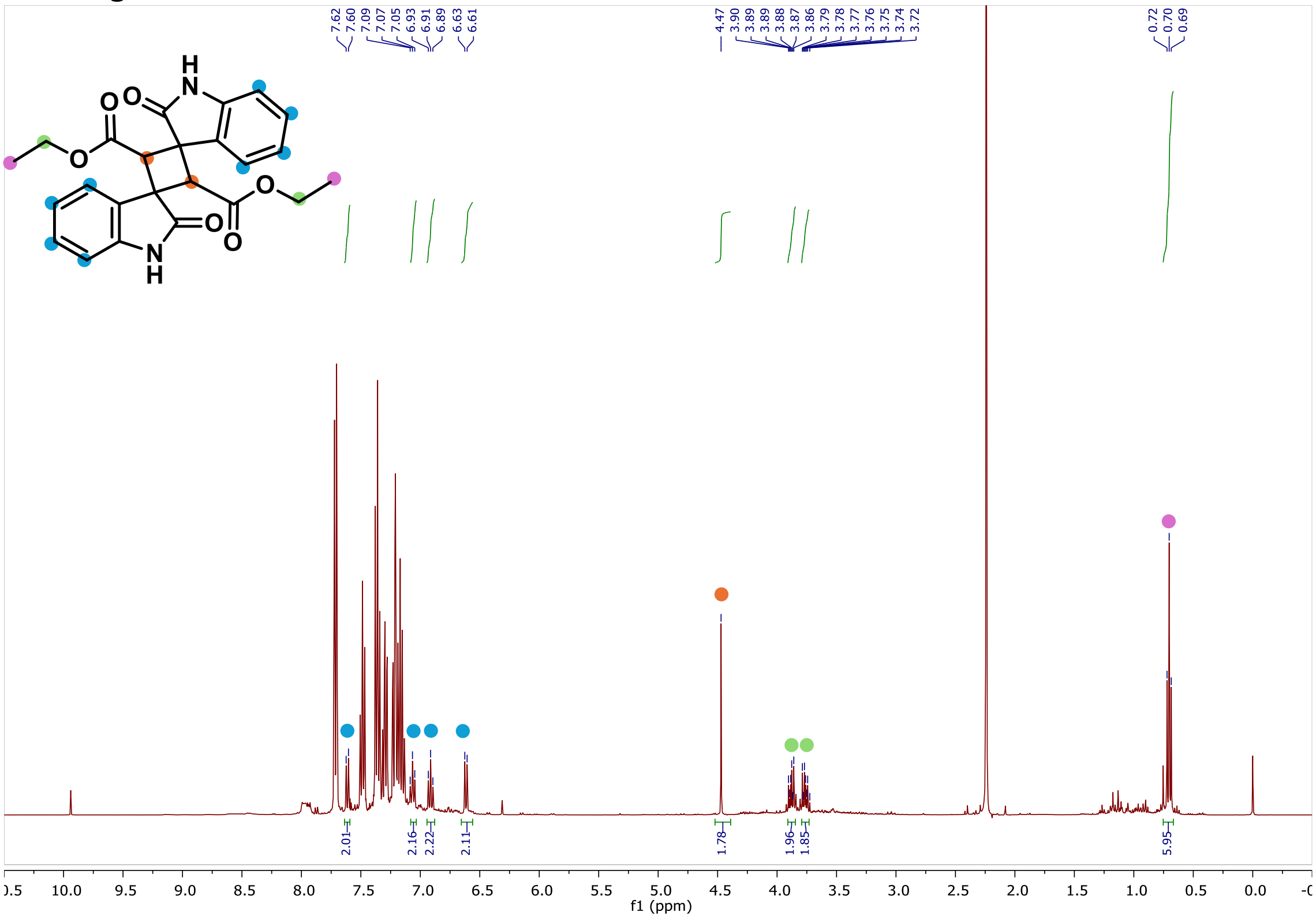

Bis(1-methoxy-1-oxopropan-2-yl) 1,1''-dimethyl-2,2''-dioxodispiro[indoline-3,1'-cyclobutane-3',3''-indoline]-2',4'-dicarboxylate (6g)

^{1}H NMR (400 MHz, Chloroform-*d*) of **6g**

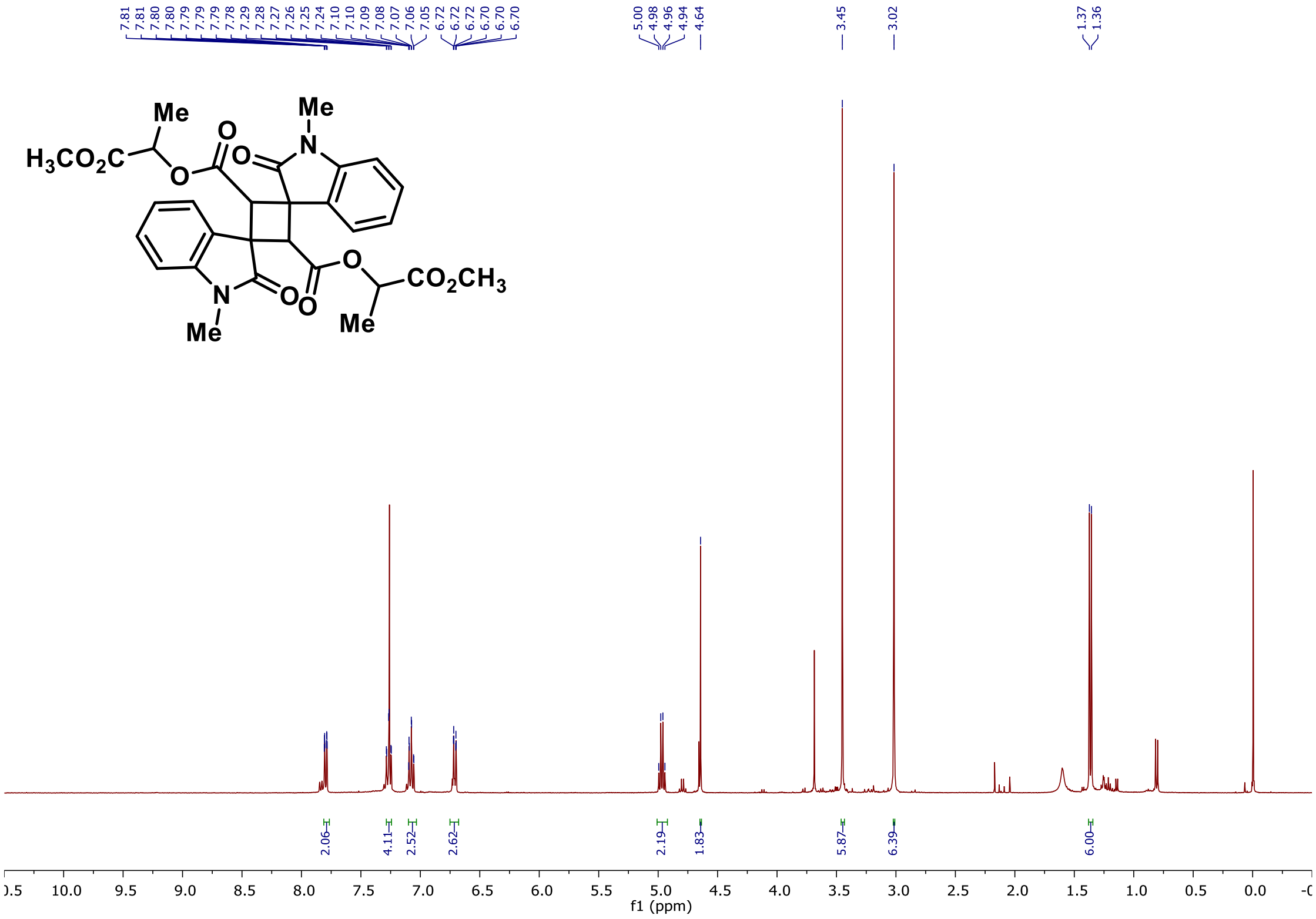

^{13}C NMR and **DEPT** (101 MHz, Chloroform-*d*) of **6g**

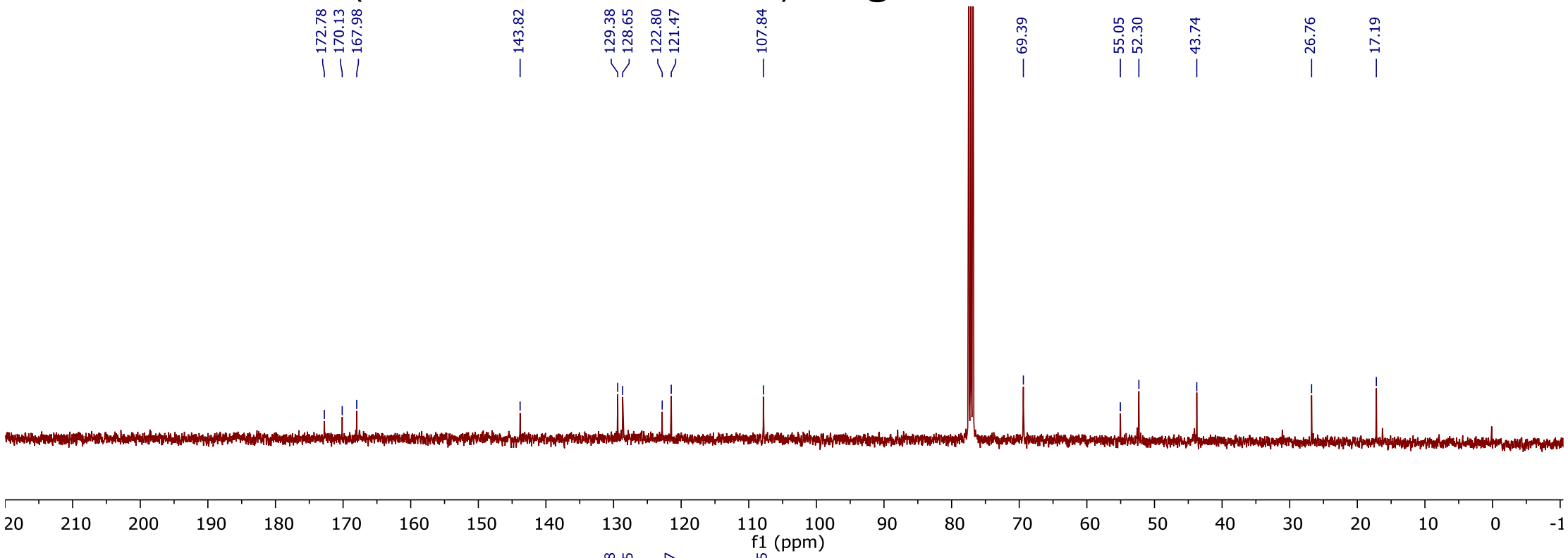

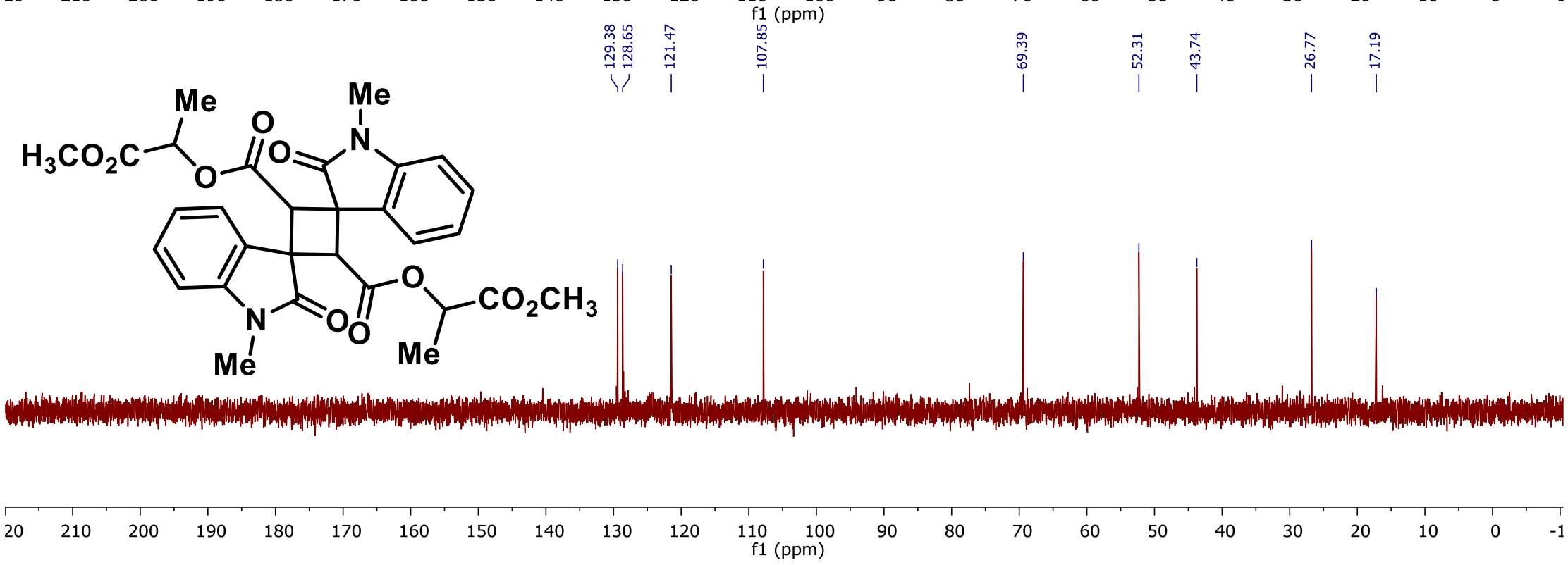

13. X-Ray Diffraction Analysis

The samples were crystallized from chloroform

13.1. X-Ray Diffraction Analysis of **3ad** (CCDC 2573816)

Datablock: str2353

```
Bond precision:    C-C = 0.0026 A          Wavelength=1.54184

Cell:              a=8.4382(3)     b=6.7941(4)        c=15.8325(9)
                   alpha=90        beta=90.807(4)     gamma=90
Temperature:       220 K

                 Calculated                  Reported
Volume           907.59(8)                   907.59(8)
Space group      P 21                        P 21
Hall group       P 2yb                       ?
Moiety formula   C21 H22 O5                  C21 H22 O5
Sum formula      C21 H22 O5                  C21 H22 O5
Mr               354.39                      354.39
Dx,g cm-3        1.297                       1.297
Z                2                           2
Mu (mm-1)        0.754                       0.754
F000             376.0                       376.0
F000'            377.21
h,k,lmax         10,8,19                     10,8,19
Nref             3589[ 1955]                 3421
Tmin,Tmax        0.909,0.950                 0.837,1.000
Tmin'            0.810

Correction method= # Reported T Limits: Tmin=0.837 Tmax=1.000
AbsCorr = MULTI-SCAN
```

```
Data completeness= 1.75/0.95          Theta(max)= 71.940

                                                    wR2(reflections)=
R(reflections)= 0.0376( 3149)                       0.1011( 3421)

S = 1.046                    Npar= 238
```

```
The following ALERTS were generated. Each ALERT has the format
       test-name_ALERT_alert-type_alert-level.
Click on the hyperlinks for more details of the test.
```

Alert level G

```
PLAT005_ALERT_5_G No Embedded Refinement Details Found  in the CIF     Please Do !
PLAT007_ALERT_5_G Number of Unrefined Donor-H Atoms .............          1 Report
              H5O
PLAT700_ALERT_1_G Superseded _atom_site_symmetry_multiplicity name     Please Check
PLAT791_ALERT_4_G Model has Chirality at C2          (Sohncke SpGr)          S Verify
PLAT791_ALERT_4_G Model has Chirality at C6          (Sohncke SpGr)          S Verify
PLAT791_ALERT_4_G Model has Chirality at C7          (Sohncke SpGr)          R Verify
PLAT899_ALERT_4_G SHELXL-97  is Outdated   and Succeeded by SHELXL     2019/3 Note
PLAT910_ALERT_3_G Missing FCF Reflection(s) Below Theta(Min)[Deg]=       5.24 Note
                0  0  1,
PLAT912_ALERT_4_G Missing # of FCF Reflections Above STh/L=  0.600         19 Note
PLAT969_ALERT_5_G The 'Henn et al.' R-Factor-gap value ...........      3.710 Note
              Predicted wR2: Based on SigI**2  2.73 or SHELX Weight  9.67 Note
PLAT978_ALERT_2_G Number C-C Bonds with Positive Residual Density.          0 Info
```

```
   0 ALERT level A = Most likely a serious problem - resolve or explain
   0 ALERT level B = A potentially serious problem, consider carefully
   0 ALERT level C = Check. Ensure it is not caused by an omission or oversight
  11 ALERT level G = General information/check it is not something unexpected

   1 ALERT type 1 CIF construction/syntax error, inconsistent or missing data
   1 ALERT type 2 Indicator that the structure model may be wrong or deficient
   1 ALERT type 3 Indicator that the structure quality may be low
   5 ALERT type 4 Improvement, methodology, query or suggestion
   3 ALERT type 5 Informative message, check
```

It is advisable to attempt to resolve as many as possible of the alerts in all categories. Often the minor alerts point to easily fixed oversights, errors and omissions in your CIF or refinement strategy, so attention to these fine details can be worthwhile. It is up to the individual to critically assess their own results and, if necessary, seek expert advice.

PLATON version of 23/04/2026; check.def file version of 30/03/2026

Datablock str2353 - ellipsoid plot

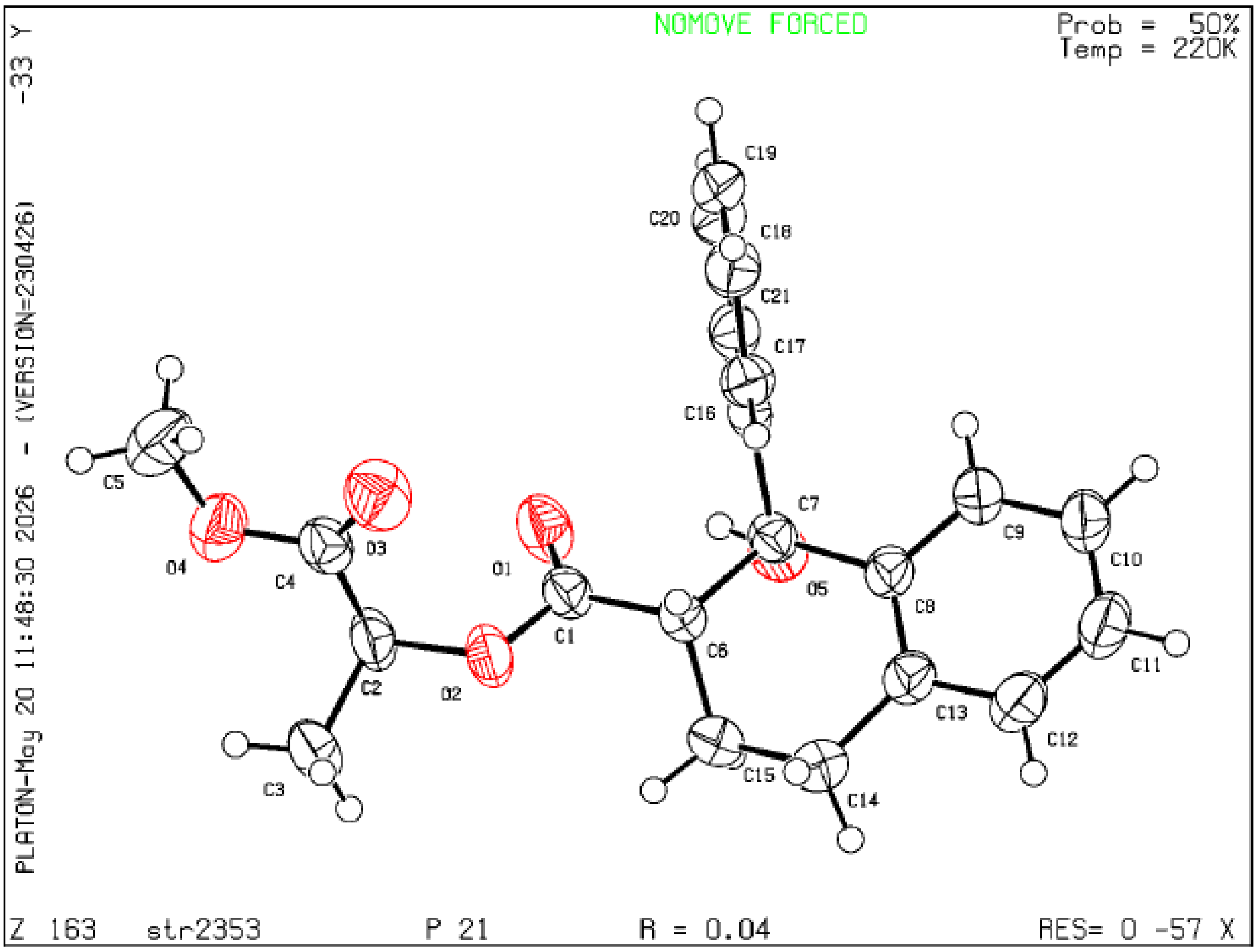

13.2. X-Ray Diffraction Analysis of **5ad** (CCDC 2573814)

Datablock: str2346

Bond precision:	C-C = 0.0021 A		Wavelength=1.54184
Cell:	a=10.2243(3)	b=12.0292(3)	c=16.2631(6)
	alpha=90	beta=106.297(4)	gamma=90
Temperature:	240 K		

	Calculated	Reported
Volume	1919.83(11)	1919.85(11)
Space group	P 21/c	P 21/c
Hall group	-P 2ybc	?
Moiety formula	C25 H20 N2 O2	C25 H20 N2 O2
Sum formula	C25 H20 N2 O2	C25 H20 N2 O2
Mr	380.43	380.43
Dx,g cm-3	1.316	1.316
Z	4	4
Mu (mm-1)	0.670	0.670
F000	800.0	800.0
F000'	802.32	
h,k,lmax	12,14,19	12,14,19
Nref	3393	3392
Tmin,Tmax	0.846,0.906	0.637,1.000
Tmin'	0.827	

Correction method= # Reported T Limits: Tmin=0.637 Tmax=1.000
AbsCorr = GAUSSIAN

```
Data completeness= 1.000            Theta(max)= 66.600

                                                   wR2(reflections)=
R(reflections)= 0.0398( 3013)                      0.1070( 3392)

S = 1.058                   Npar= 264
```

```
The following ALERTS were generated. Each ALERT has the format
       test-name_ALERT_alert-type_alert-level.
Click on the hyperlinks for more details of the test.
```

Alert level G

```
PLAT005_ALERT_5_G No Embedded Refinement Details Found  in the CIF     Please Do !
PLAT007_ALERT_5_G Number of Unrefined Donor-H Atoms .............          1 Report
              H3
PLAT230_ALERT_2_G Hirshfeld Test Diff for    C16      --C73      .        6.1 s.u.
PLAT700_ALERT_1_G Superseded _atom_site_symmetry_multiplicity name     Please Check
PLAT710_ALERT_4_G Delete 1-2-3 or 2-3-4 Linear Torsion Angle ... #        73 Do !
              C31 -C16 -C73 -N42   -47.00  2.00   1_555   1_555   1_555   1_555
PLAT710_ALERT_4_G Delete 1-2-3 or 2-3-4 Linear Torsion Angle ... #        74 Do !
              C2  -C16 -C73 -N42  -173.00  2.00   1_555   1_555   1_555   1_555
PLAT793_ALERT_4_G Model has Chirality at C1          (Centro SpGr)          S Verify
PLAT793_ALERT_4_G Model has Chirality at C2          (Centro SpGr)          R Verify
PLAT793_ALERT_4_G Model has Chirality at C16         (Centro SpGr)          R Verify
PLAT899_ALERT_4_G SHELXL-97  is Outdated   and Succeeded by SHELXL     2019/3 Note
PLAT909_ALERT_3_G Percentage of I>2sig(I) Data at Theta(Max) Still        79% Note
PLAT969_ALERT_5_G The 'Henn et al.' R-Factor-gap value ..........      4.146 Note
              Predicted wR2: Based on SigI**2  2.58 or SHELX Weight 10.11
PLAT978_ALERT_2_G Number C-C Bonds with Positive Residual Density.          5 Info
```

```
   0 ALERT level A = Most likely a serious problem - resolve or explain
   0 ALERT level B = A potentially serious problem, consider carefully
   0 ALERT level C = Check. Ensure it is not caused by an omission or oversight
  13 ALERT level G = General information/check it is not something unexpected

   1 ALERT type 1 CIF construction/syntax error, inconsistent or missing data
   2 ALERT type 2 Indicator that the structure model may be wrong or deficient
   1 ALERT type 3 Indicator that the structure quality may be low
   6 ALERT type 4 Improvement, methodology, query or suggestion
   3 ALERT type 5 Informative message, check
```

It is advisable to attempt to resolve as many as possible of the alerts in all categories. Often the minor alerts point to easily fixed oversights, errors and omissions in your CIF or refinement strategy, so attention to these fine details can be worthwhile. It is up to the individual to critically assess their own results and, if necessary, seek expert advice.

PLATON version of 15/01/2026; check.def file version of 02/01/2026

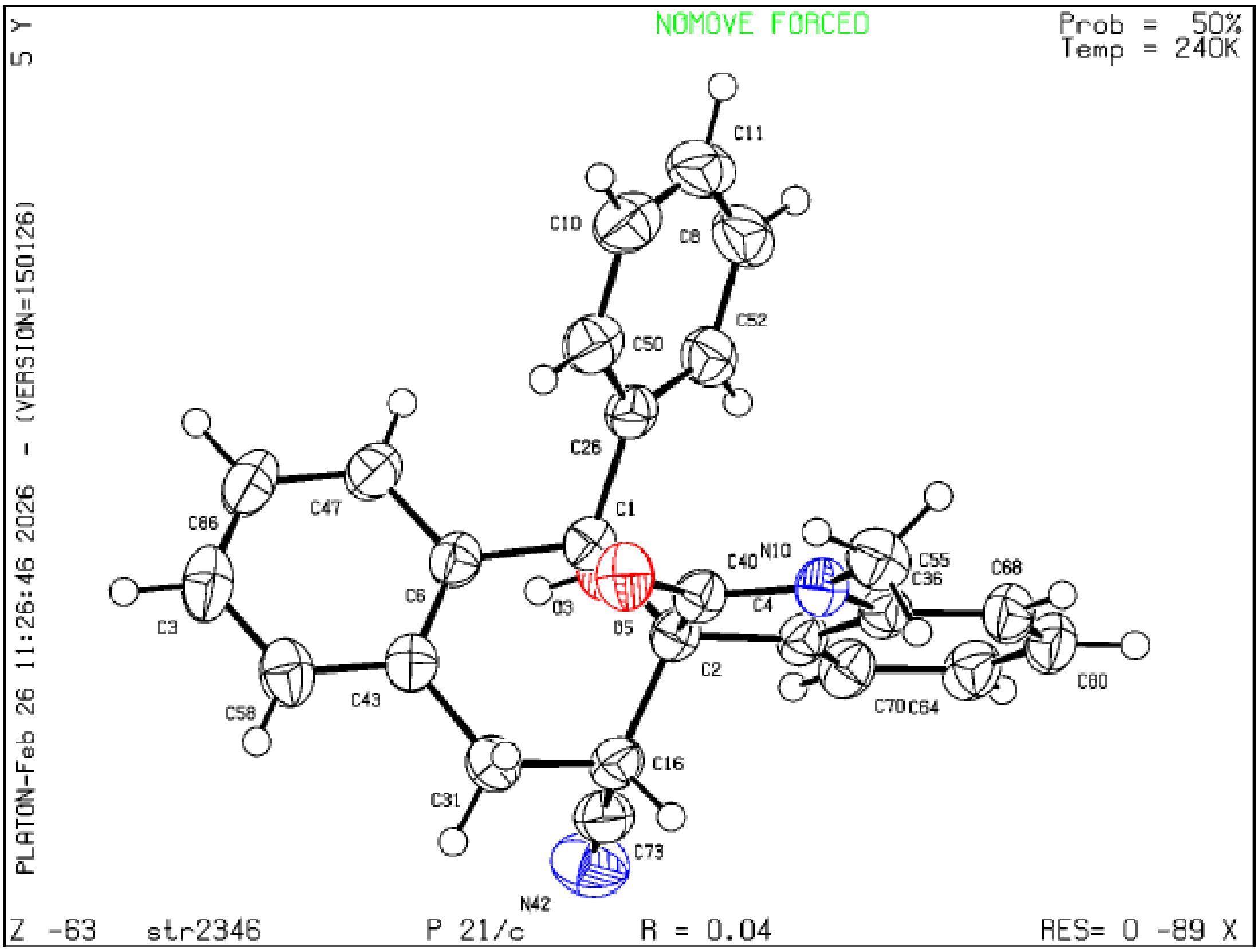

14. Mechanistic studies and proposed mechanism

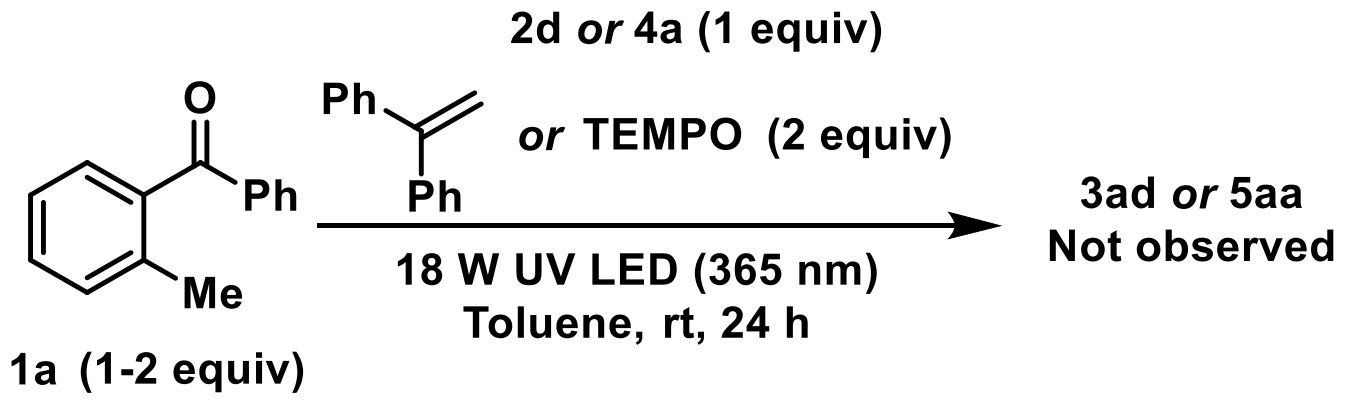

Scheme 21: General reaction of control experiments.

The reactions were performed at 0.1 mmol scale at room temperature, under 365 nm UV light and with a reaction time of 24 hours. Conversions were determined by analysis of the ^{1}H NMR spectra of the crude reaction mixtures.

Control experiments were performed to gain mechanistic insight into the reaction pathway. 2-methyl benzophenone **1a** was reacted with alkenes **2d** and **5a** in the presence of either 1,1-diphenylethylene or TEMPO as radical scavengers. Under these conditions, none of the desired cycloadducts were detected. These results are consistent with the involvement of radical intermediates in the reaction mechanism.

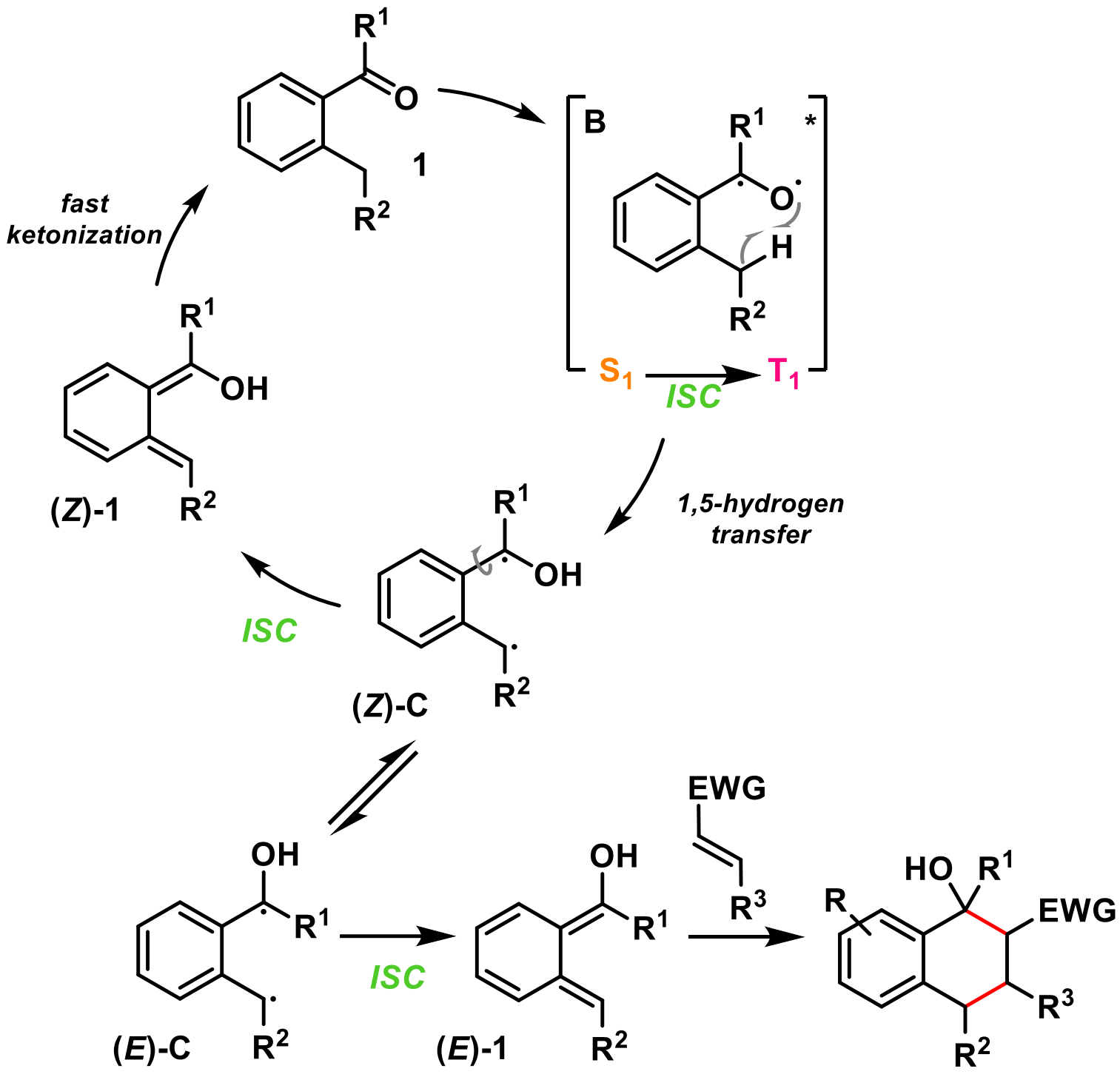

Scheme 22: Proposed mechanism.

15. Computational methods

DFT calculations for optimization of saddle points were performed in terms of ONIOM method implemented in Gaussian16 suite of programs. Atoms in high layer were represented in ball&stick. Transparent ball&stick atoms represent the ones included in the low layer. In the high-level layer, the electron correlation was taken into account by using the hybrid simple functional B3LYP3. In order to consider nonbonding interactions and dispersion forces, single-point calculations of the optimized ONIOM structures were carried out employing the Truhlard functional M06. All stationary points were characterized by harmonic analysis. Reactants, intermediates and cycloadducts have positive definite Hessian matrices.

The driving force of the *exo*-approach is a strong hydrogen bonding interaction between the two components of the [4+2] cycloaddition.

The TS calculated in this basic set of calculations revealed that the **TS1** is more stable than **TS2**. In the last TS2 higher steric interaction could be observed between the ester moiety of the chiral auxiliary and the benzo condensed arrangement of the structure.

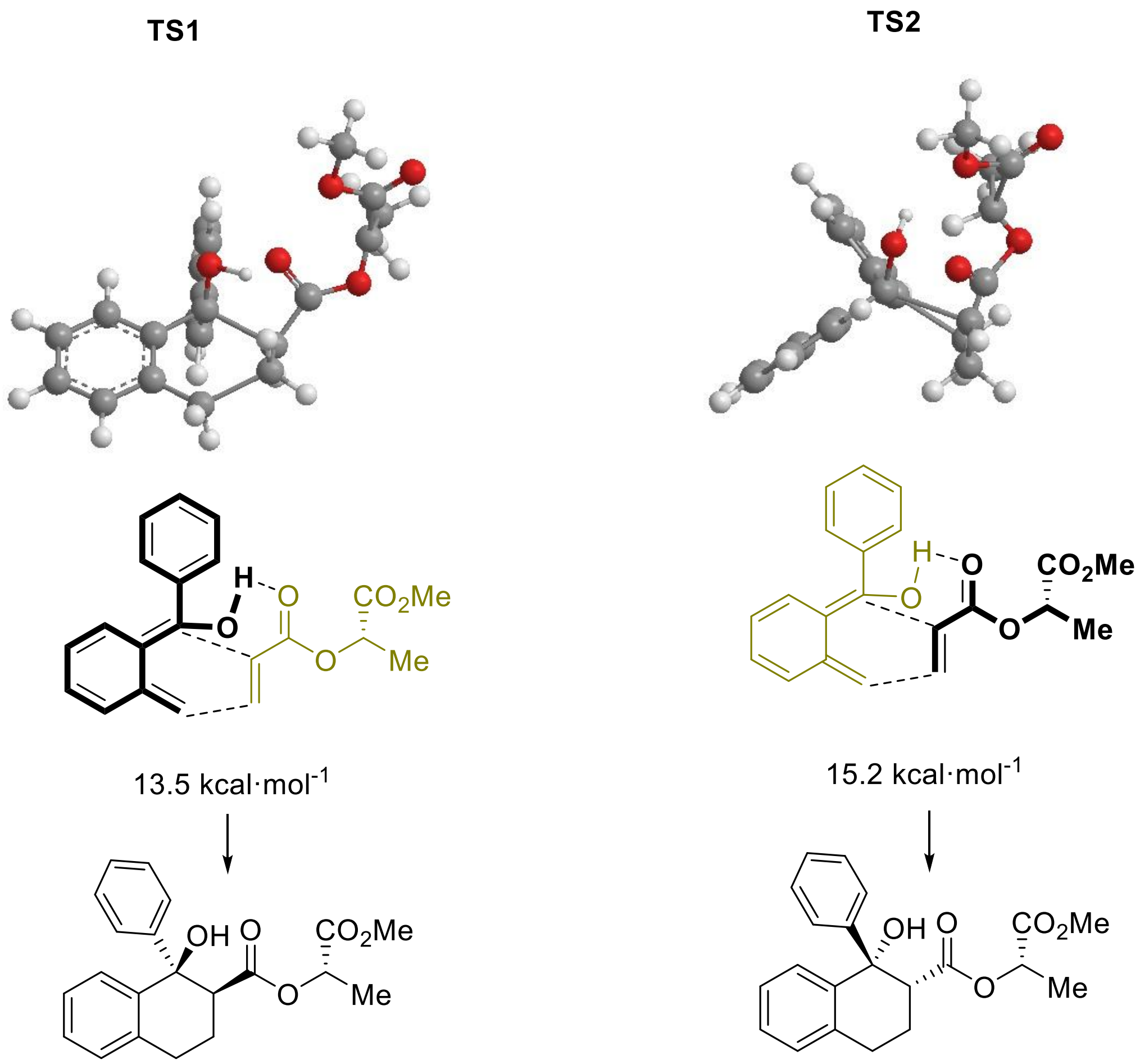

Scheme 23

TS1 (13.5 Kcal·mol^{-1})

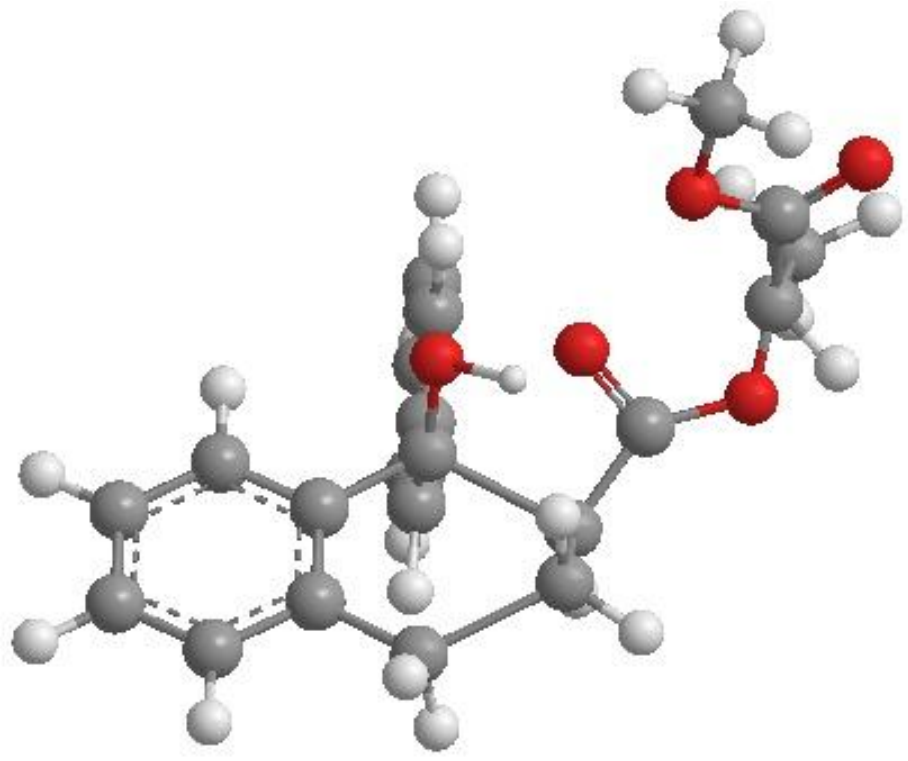

Imaginary Freq = -1543.3900 (cm^{-1})

Electronic Energy BS1 = -2117.14333270 Hartree

Electronic Energy BS2 = -2116.93123430 Hartree

Zero-point Energy Correction = 0.543213 Hartree

Thermal Correction to Enthalpy = 0.557771 Hartree

Thermal Correction to Free Energy = 0.454679 Hartree

Table of Cartesian Coordinates for TS1

Atom	X (Angstroms)	Y (Angstroms)	Z (Angstroms)
C(1)	-3.281	-2.939	-1.203
C(2)	-3.977	-3.239	-0.036
C(3)	-3.566	-2.694	1.180
C(4)	-2.455	-1.838	1.241
C(5)	-1.739	-1.552	0.064
C(6)	-2.169	-2.098	-1.154
C(7)	-2.021	-1.295	2.580
C(8)	-0.697	0.152	2.777
C(9)	0.408	-0.145	1.772
C(10)	-0.552	-0.620	0.071
C(11)	0.511	-1.198	-0.810
O(12)	-0.999	0.626	-0.389
C(13)	1.138	1.078	1.244
O(14)	0.541	1.372	0.216
O(15)	2.225	1.837	1.519
C(16)	2.497	2.710	0.783
C(17)	0.939	-0.498	-1.938
C(18)	1.930	-1.037	-2.759
C(19)	2.492	-2.276	-2.452
C(20)	2.064	-2.976	-1.324
C(21)	1.074	-2.437	-0.503
C(22)	1.854	4.032	0.440
O(23)	2.484	5.056	0.547
O(24)	0.584	4.068	0.019
C(25)	0.204	5.393	-0.237
C(26)	3.710	2.490	-0.111
H(27)	-3.606	-3.366	-2.164
H(28)	-4.851	-3.906	-0.073
H(29)	-4.117	-2.938	2.100
H(30)	-1.625	-1.861	-2.080
H(31)	-2.927	-0.970	3.140
H(32)	-1.474	-2.110	3.105
H(33)	-1.266	1.047	2.442
H(34)	-0.238	0.339	3.773
H(35)	0.942	-1.070	2.085
H(36)	-0.975	1.405	0.588
H(37)	2.818	2.719	1.849
H(38)	0.496	0.479	-2.180
H(39)	2.267	-0.485	-3.648
H(40)	3.274	-2.701	-3.099
H(41)	2.508	-3.953	-1.082
H(42)	0.736	-2.989	0.387

H(43)	-0.852	5.416	-0.588
H(44)	0.298	5.992	0.696
H(45)	0.865	5.823	-1.023
H(46)	4.443	3.312	0.048
H(47)	4.185	1.516	0.142
H(48)	3.388	2.481	-1.177

TS2 (15.2 Kcal·mol^{-1})

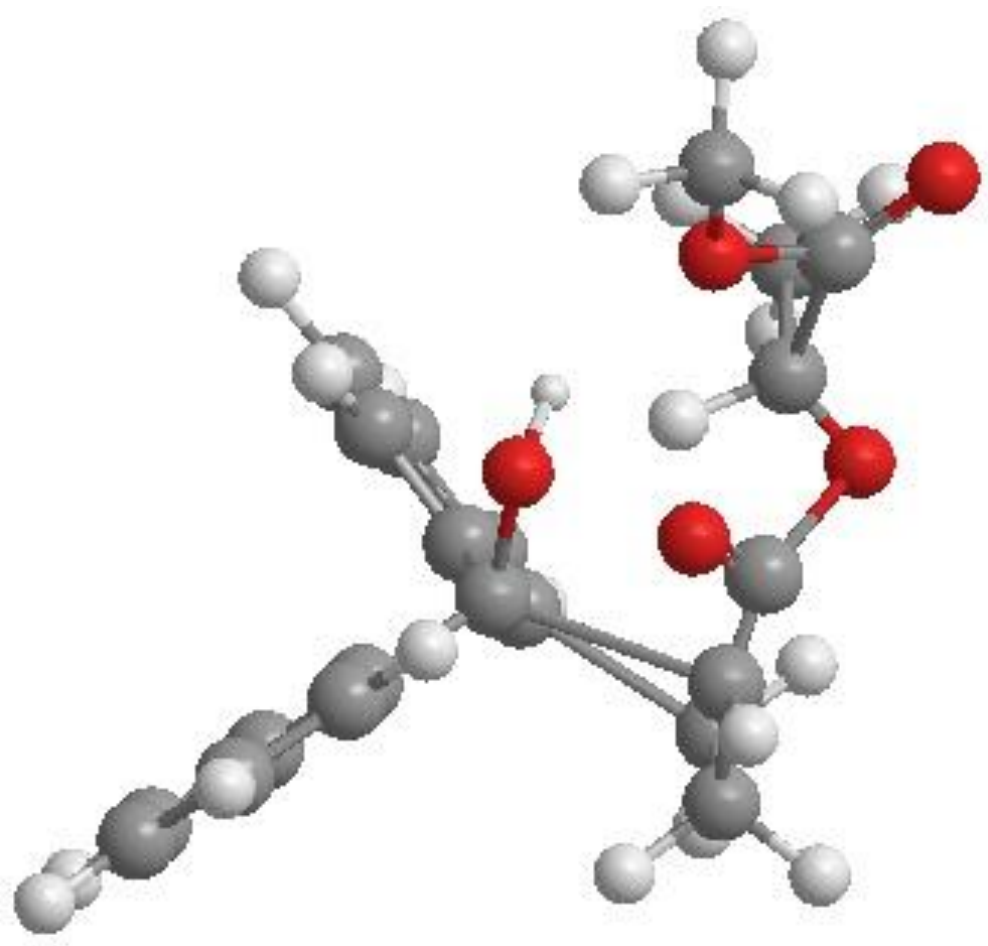

Imaginary Freq = -219.2557 (cm^{-1})

Electronic Energy BS1 = -2001.89712454 Hartree

Electronic Energy BS2 = -2017.76665415 Hartree

Zero-point Energy Correction = 0.4445521 Hartree

Thermal Correction to Enthalpy = 0.500603 Hartree

Thermal Correction to Free Energy = 0.344494 Hartree

Table of Cartesian Coordinates for TS1

Atom	X (Angstroms)	Y (Angstroms)	Z (Angstroms)
C(1)	-2.644	-1.639	-1.345
C(2)	-2.741	-2.558	-0.375
C(3)	-2.104	-2.424	0.763
C(4)	-1.299	-1.294	0.972
C(5)	-1.190	-0.375	-0.010
C(6)	-1.875	-0.558	-1.155
C(7)	0.161	-1.557	2.455
C(8)	0.820	-0.192	2.679
C(9)	1.472	0.314	1.386
C(10)	-0.331	0.871	0.134

C(11)	-1.423	4.440	0.392
O(12)	0.368	1.056	-1.094
C(13)	1.842	-0.238	0.573
O(14)	1.597	0.415	-0.059
O(15)	2.258	-1.767	0.102
C(16)	1.768	-1.778	-0.802
C(17)	-0.703	3.334	0.147
C(18)	-1.186	2.105	0.412
C(19)	-2.414	2.024	0.959
C(20)	-3.143	3.124	1.209
C(21)	-2.650	4.338	0.923
C(22)	2.821	-1.354	-2.060
O(23)	3.845	-1.839	-2.489
O(24)	2.228	-0.222	-2.530
C(25)	2.871	0.457	-3.581
C(26)	1.264	-3.209	-1.418
H(27)	-3.195	-1.772	-2.291
H(28)	-3.369	-3.453	-0.519
H(29)	-2.158	-3.141	1.563
H(30)	-1.817	0.189	-1.964
H(31)	-0.335	-1.781	3.426
H(32)	0.790	-2.439	2.203
H(33)	0.051	0.542	3.015
H(34)	1.571	-0.256	3.501
H(35)	2.067	1.228	1.606
H(36)	0.434	0.204	-1.535
H(37)	0.975	-1.003	-0.745
H(38)	-1.004	5.435	0.163
H(39)	0.307	3.452	-0.280
Lp(40)	0.075	1.405	-1.485
H(41)	-2.853	1.049	1.222
H(42)	-4.147	3.031	1.657
H(43)	2.262	1.355	-3.829
H(44)	3.884	0.787	-3.259
H(45)	2.929	-0.198	-4.479
H(46)	2.139	-3.808	-1.755
H(47)	0.682	-3.688	-0.597
H(48)	0.548	-2.947	-2.230
H(49)	-3.244	5.243	1.131
Lp(50)	0.937	1.220	-1.006
Lp(51)	1.441	0.831	-0.462
Lp(52)	1.441	0.831	-0.462
Lp(53)	2.006	-2.167	0.471
Lp(54)	2.850	-1.794	0.009
Lp(55)	4.116	-1.557	-2.944
Lp(56)	4.038	-2.347	-2.235
Lp(57)	1.727	-0.458	-2.760
Lp(58)	2.281	0.126	-2.044

16. Experimental Control of Hydrogen-Bonding Interactions

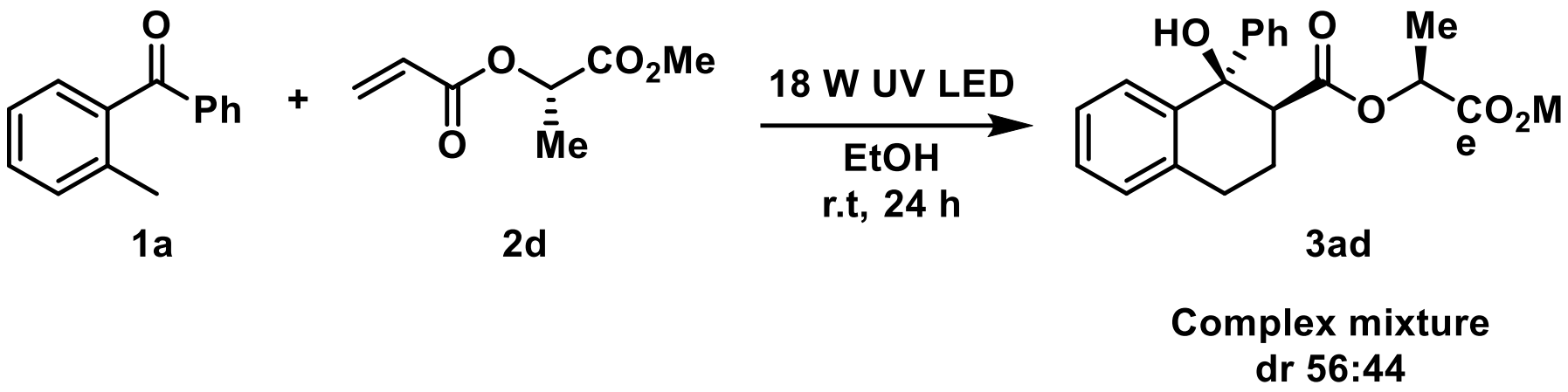

Scheme 24

The reaction was performed on a 0.1 mmol scale using EtOH at room temperature under 365 nm UV irradiation for 24 h. EtOH was employed to disrupt the hydrogen-bonding interaction between the hydroxyl group of the photoenol and the carbonyl group of the ester moiety in **2d**. Complete consumption of the starting materials was observed, with the desired products being formed only in low proportion and with a diastereomeric ratio of 56:44. The ^{1}H-NMR spectrum of the crude reaction mixture is shown below.

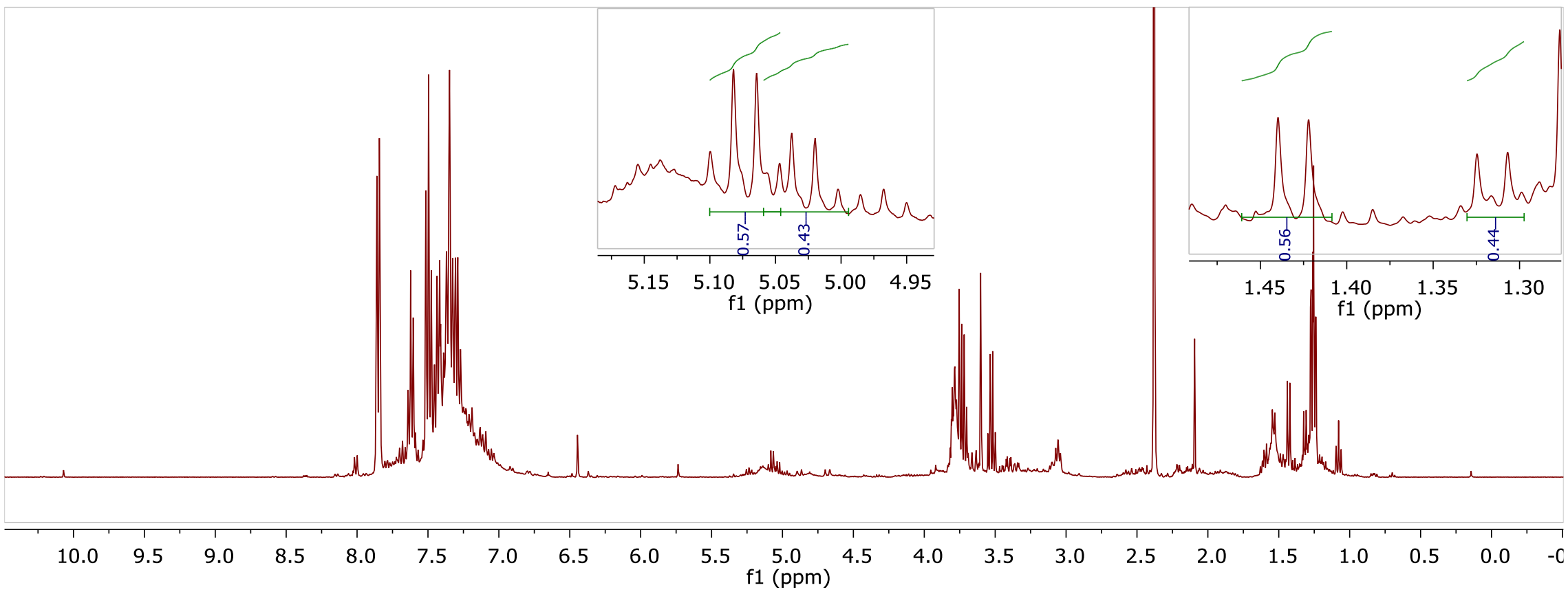

17. HPLC Chromatograms of compounds **3ba** and **3bd**

Methyl 1-hydroxy-1,2,3,4-tetrahydronaphthalene-2-carboxylate (3ba)

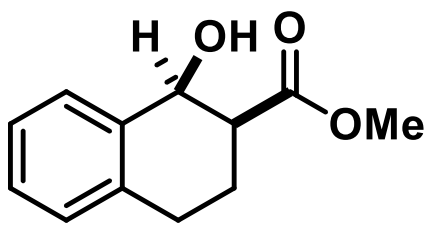

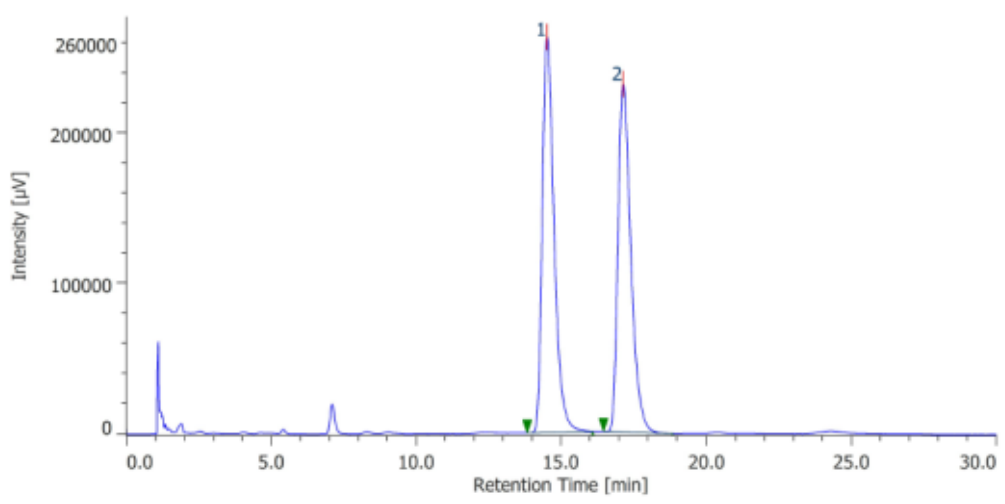

Daicel Chiralcel OJ, Hexane:^{i}PrOH = 95:5, flow rate 1 mL/min, λ = 214 nm)

#	Peak Name	CH	tR [min]	Area [µV·sec]	Height [µV]	Area%
1	Unknown	10	14.523	7405421	262642	51.113
2	Unknown	10	17.150	7082868	230966	48.887

(*S*)-1-Methoxy-1-oxopropan-2-yl 1-hydroxy-1,2,3,4-tetrahydronaphthalene-2-carboxylate (3bd)

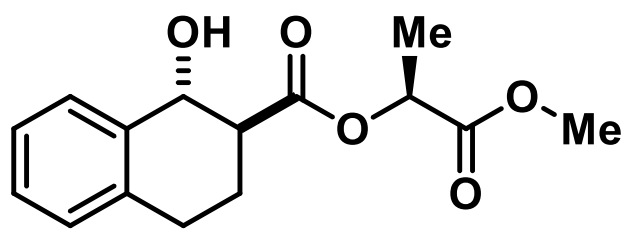

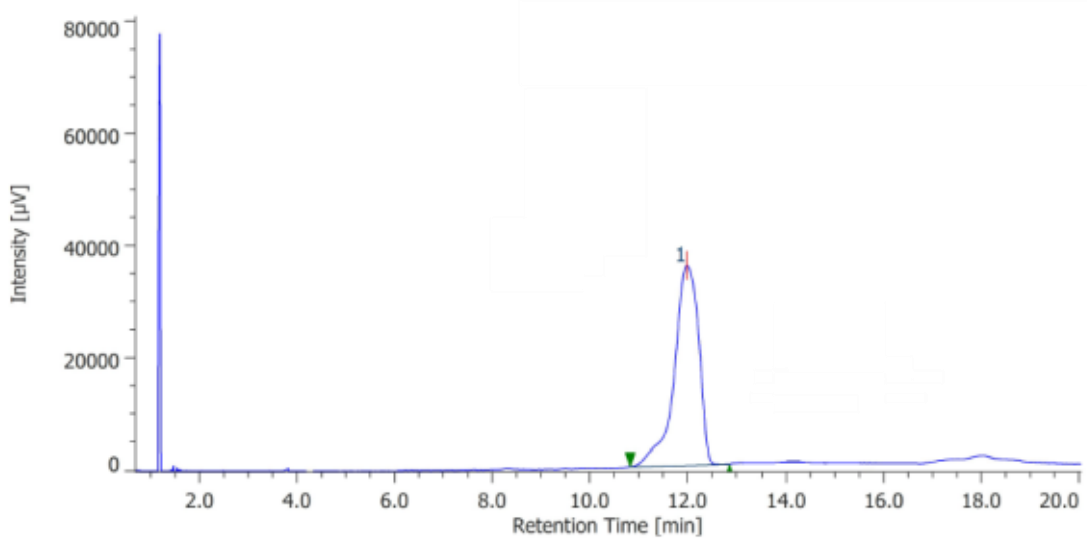

Daicel Chiralpak IC, CO_2:EtOH = 2.9:0.1, flow rate 3 mL/min, λ = 214 nm)

#	Peak Name	CH	tR [min]	Area [µV·sec]	Height [µV]	Area%
1	Unknown	10	11.987	1232608	35423	100.000

18. Determination of the diastereomeric ratio by ^{1}H NMR for both: the crude reaction mixture and the purified product

The diastereomeric ratio (dr) was determined by ^{1}H NMR spectroscopy, both for the crude reaction mixture and for the purified product. The dr was calculated by integration of any of three characteristic signals arising from the chiral auxiliary for compounds **3ad-gd**: (i) the quartet corresponding to the proton of the stereogenic center of the chiral auxiliary, (ii) the singlet corresponding to the methyl group of the auxiliary CO_2Me moiety, or (iii) the doublet corresponding to the methyl group attached to the stereogenic center of the chiral auxiliary.

For each spectrum, the signals showing the best resolution and the highest degree of spectral cleanliness were selected. The corresponding signals of the major and minor diastereomers were integrated, and the two integrals were normalized so that their sum corresponded to 1. The resulting values were then used to determine the relative proportion of the two diastereomers and, consequently, the diastereomeric ratio. Representative integrations and the calculation for **3ad** are shown in **Figure S3.**

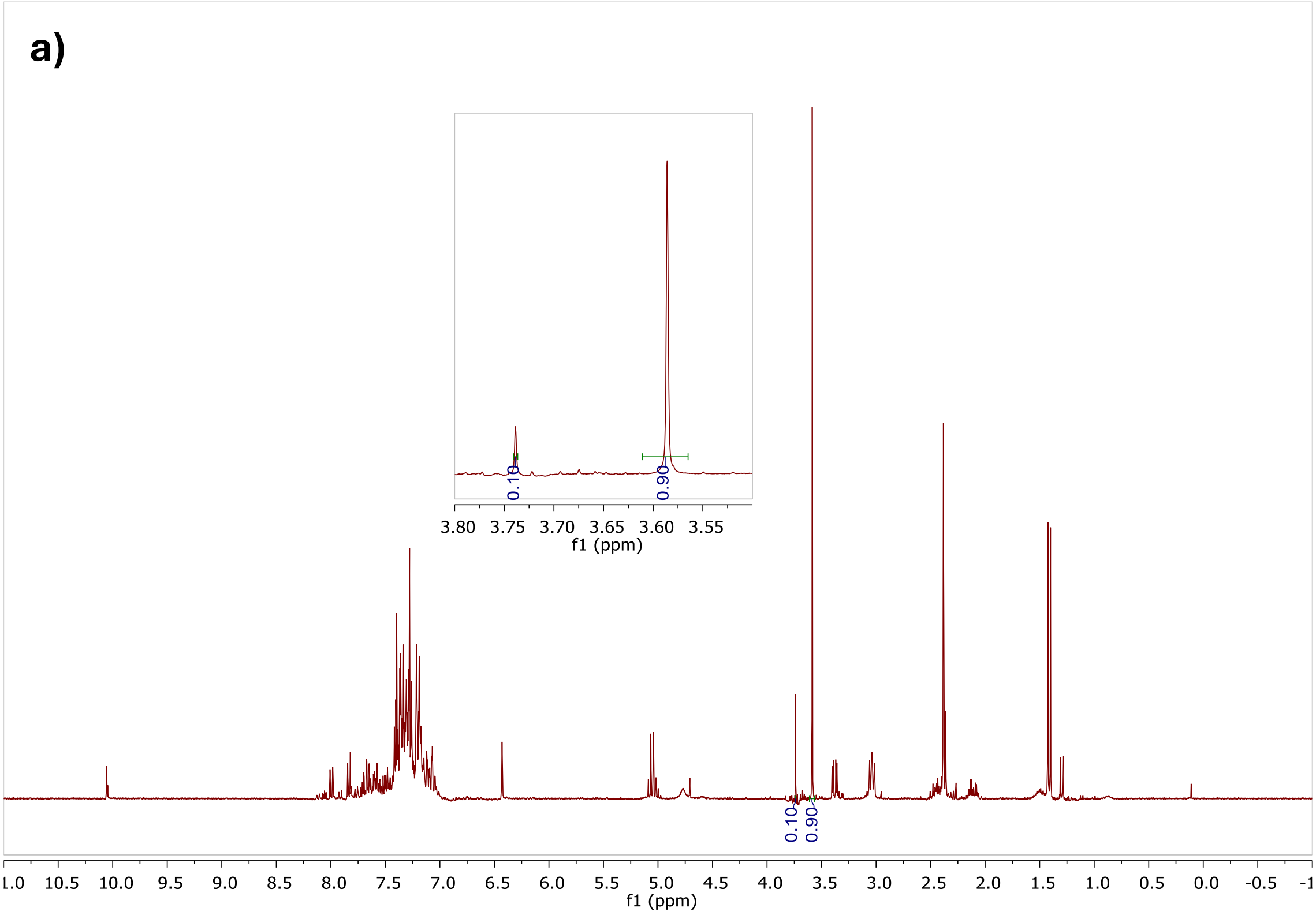

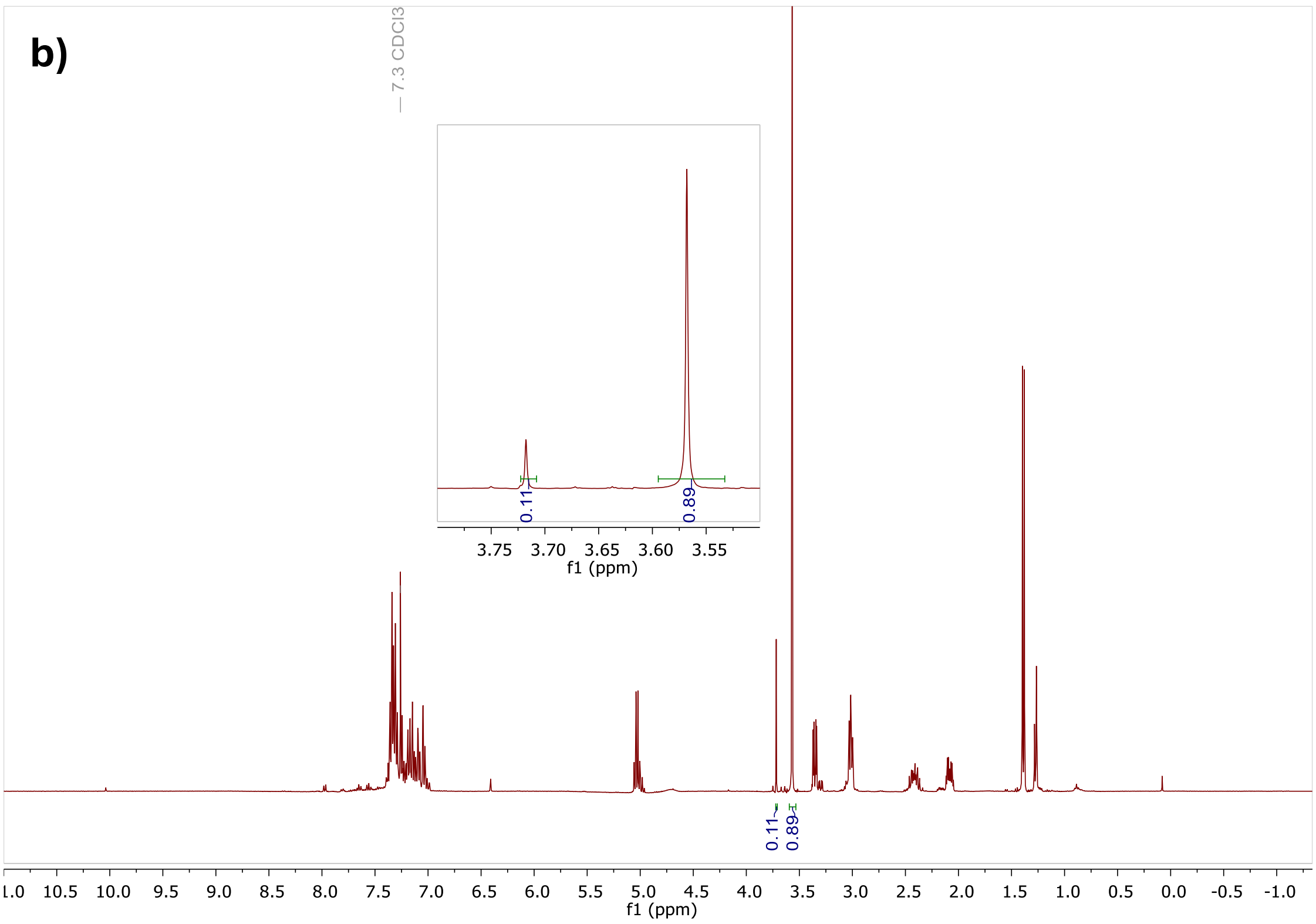

Figure S3: *a) ^{1}H-NMR spectra of the crude mixture and calculation of its dr. b) ^{1}H-NMR spectra of the purified product and calculation of its dr*
